# Genetic Variation in the Main Cultivar Collection of *Castanea henryi* Revealed by Genome Resequencing

**DOI:** 10.3390/cimb48020173

**Published:** 2026-02-03

**Authors:** Yifan Wang, Xueting Yuan, Jinhui Yang, Xibing Jiang, Shipin Chen, Hui Chen, Yu Li

**Affiliations:** 1 College of Forestry, Fujian Agriculture and Forestry University, Fuzhou 350002, China; 15334835566@163.com (Y.W.); yxt15809913171@163.com (X.Y.); 13466030524@163.com (J.Y.); fjcsp@126.com (S.C.); huichenfafu@163.com (H.C.); 2 Research Institute of Subtropical Forestry, Chinese Academy of Forestry, Hangzhou 311400, China; jxb912@126.com

**Keywords:** *Castanea henryi*, genome resequencing, single-nucleotide polymorphism, cultivar, genetic variation

## Abstract

*Castanea henryi* is an important economic tree species in China. Its nutrient-rich nuts play a key role in raising farmers’ income in mountainous areas, promoting forestry industry development, and maintaining ecological balance, thereby providing significant economic and ecological value. To systematically elucidate the genetic characteristics of major *C. henryi* cultivars in China, this study conducted phenotypic trait measurements on 42 cultivars collected from Taining and Jian’ou in Fujian Province. Combined with whole-genome resequencing technology and using the *C. henryi* genome as a reference, systematic analyses were carried out. The results indicated that the Jian’ou group (HJO) generally exhibited superior performance in key fruit phenotypic traits compared to the Taining group (HTNC), with greater phenotypic diversity observed within the HJO group. Clustering analysis of phenotypic traits further revealed a cross-geographic convergent clustering pattern among the 42 *C. henryi* cultivars. Further analysis revealed that the overall genetic diversity of the 42 *C. henryi* cultivars was relatively low (observed heterozygosity: HJO = 0.0275, HTNC = 0.0194). Notably, parameters such as heterozygosity, minor allele frequency, nucleotide polymorphism, and polymorphic information content were slightly higher in the Jian’ou group compared to the Taining group. Divergent selection signal analysis (*Fst* top 5%) identified 3129 genomic regions under divergent selection. Genes within these regions showed homology to 1205 *Arabidopsis thaliana* genes, reflecting adaptive divergence driven by differential historical selection pressures between the two groups. Population genetic structure analysis indicated that the two regional groups exhibit high genetic similarity and low differentiation. This study reveals low genetic diversity and high genetic background homogeneity among *C. henryi* cultivars, findings that could inform the design of future breeding strategies.

## 1. Introduction

At present, seven species of *Castanea* plants within the Fagaceae family are recognized globally, among which *Castanea henryi* is endemic to China. Compared to its relatives, *C. mollissima* and *C. seguinii*, *C. henryi* stands out for its nutrient-rich nuts, which are abundant in starch, sugar, protein, vitamins, and other substances that are essential for human health. Its distinctive sticky flavor has earned it the distinguished moniker ‘the crop of the mountains’ [1,2,3]. As a vital tree species in southern China, *C. henryi* embodies the functional attributes of a woody food plant and the dual purpose of producing fruit and timber [4]. Its cultivation has become a cornerstone of agricultural development in underdeveloped regions of southern Zhejiang and northern Fujian, driving regional economic growth, optimizing industrial structures, and fostering ecological synergy. Given its economic and ecological significance, the *C. henryi* industry has thus emerged as a pivotal force in promoting sustainable development across these areas [5]. *C. henryi* is primarily distributed in the Qinling Mountains and areas south of the Huaihe River, spanning 14 provinces [6]. Among these, Fujian Province, one of China’s major *C. henryi* production areas, boasts a long history of cultivation and rich genetic diversity. Its industry has expanded from a local to a national scale and has even been internationally exported. The rich genetic resources in this region are a key factor driving the development of the *C. henryi* industry, and lay the foundation for breeding research on cultivated varieties. The extensive genetic exchange among these cultivars has facilitated the development of numerous popular varieties [7].

In China, the systematic breeding of *C. henryi* began in the late 1980s through collaboration between technicians and local farmers to identify superior genotypes from wild and cultivated populations. Elite individuals were selected via rigorous phenotypic and genetic evaluation and were clonally propagated for large-scale distribution. These genetically distinct clones formed discrete cultivated populations characterized by high adaptability, enhanced stress resistance, and superior nut quality [8,9]. Chestnuts grow in forests or in the wild, so that a rich variation has been accumulated throughout the history of domestication. The selection of natural variations is a rapid method for Chestnut tree breeding. As the cornerstone of the regional industry, these varieties have driven economic development and serve as vital germplasm for continuous genetic improvement, illustrating the species’ long-term breeding trajectory [10].

Genetic diversity, referring to the variation in genetic material within and among populations, serves as a cornerstone of biological diversity. It plays a pivotal role in elucidating evolutionary processes, assessing the adaptive potential of extant organisms, and predicting their future evolutionary trajectories [11]. Martín M. et al. used seven microsatellite loci to analyze 16 natural populations distributed across the range of chestnuts in Spain. Their findings revealed regions of significant genetic diversity, indicating that Spanish chestnut populations have a high degree of genetic variability, largely corresponding to their geographic distribution [12]. This discovery provided invaluable foundational data for a subsequent in-depth landscape genetics study. Similarly, Pereira-Lorenzo conducted a nationwide census, identification, and geolocation of chestnut trees in Switzerland, culminating in the genetic analysis of 962 samples. Utilizing 24 microsatellite markers for genotyping, they elucidated the genetic diversity and structure of Swiss chestnut populations [13]. Concurrently, Gu et al. investigated the genetic diversity of 25 primary *C. henryi* cultivars in Taining County, Fujian Province, employing 10 pairs of SSR primer combinations [14]. Their work serves as a critical reference for the enhancement of the *C. henryi* germplasm in China. The molecular markers identified in these studies not only offer a crucial reference for the exploration of genetic diversity within chestnut germplasm cultivars but also establish a scientific foundation for their selection and conservation.

Genome resequencing technology is a key tool in the field of molecular biology, enabling comprehensive genome-wide resequencing of species with known genome sequences, thereby accurately identifying single-nucleotide polymorphisms (SNPs) [15]. SNPs are widely distributed throughout the genome with a relatively uniform distribution, exhibiting high stability and genetic conservation, and typically manifest as easily detectable and analyzable biallelic variations. These characteristics make SNPs ideal molecular markers for exploring genetic diversity. The large amount of SNP data obtained from genome resequencing enables an in-depth analysis of genetic variation among different populations [16,17] and individuals within species [18,19,20], thereby achieving a precise quantification of genetic diversity. This, in turn, helps us to understand the evolutionary trajectory and adaptive mechanisms of species and provides a basis for formulating scientifically sound strategies for the conservation and utilization of genetic resources.

In this study, we performed whole-genome resequencing on 42 *C. henryi* cultivars and screened high-quality SNP sites using the *C. henryi* reference genome previously assembled by our team. Meanwhile, the key fruit traits were measured. Our objective is to characterize the genetic diversity and population structure among different *C. henryi* cultivars, thereby providing important evidence for subsequent genetic improvement and the selection of superior trees.

## 2. Materials and Methods

### 2.1. Plant Materials

The plant materials utilized in this study comprised 42 cultivated varieties of *C. henryi* collected from northern Fujian Province, China. All cultivars are local landraces whose origin and historical cultivation are strictly confined to their respective sampling regions. Based on their geographical origins, the accessions were categorized into two groups: Jian’ou (HJO) and Taining (HTNC). For each cultivar, a single superior phenotype was selected and clonally propagated by being grafted onto one-year-old seedling rootstocks derived from wild *C. henryi* seeds. The resulting clonal germplasm was established in an experimental field in Dongyou Town, Jian’ou City (118°44′ E, 27°04′ N), Fujian Province, in 2021. This region is characterized by a subtropical monsoon climate, with an average annual sunshine duration of 1813 h, a mean annual temperature of 18.7 °C, and annual precipitation ranging from 1600 to 1800 mm. The mean annual evaporation is 1458 mm, the average relative humidity is 81%, and the frost-free period is approximately 277 days. The field trial was arranged in a completely randomized block design with three independent blocks serving as biological replicates. Within each block, each cultivar was represented by ten grafted trees planted at a spacing of 4 × 4 m. Technical replicates were implemented at the sampling level by randomly collecting three fruits from each selected representative tree per block for analysis. Detailed information on the plant materials is provided in Supplementary File S1.

### 2.2. Phenotypic Trait Measurement

In October 2025, with cultivar serving as the unified unit of analysis, one representative tree was selected from each experimental block for every cultivar. Three fruits were randomly harvested from each selected tree, resulting in a total of nine fruits per cultivar. All fruits were promptly transported to the laboratory on the day of collection and measured for key fruit traits, including size index, bur weight, bur thickness, bur spines length, nut weight, nut height, nut length, and nut width. The size index was calculated as (nut length + nut width)/2. Prior to statistical analysis, all phenotypic variables were standardized using Z-score normalization. Box plots were generated using Origin 2024 (https://www.originlab.com, accessed on 15 October 2025) to visualize the phenotypic data. In R 4.4.1 (https://www.r-project.org, accessed on 15 October 2025), permutational multivariate analysis of variance (PERMANOVA) was performed to assess differences between groups. Hierarchical clustering analysis was conducted based on Euclidean distances calculated from the standardized data, using the Ward.D2 method.

### 2.3. DNA Extraction and Sequencing

Young leaves were carefully selected from healthy and disease-free plants. They were then transported to the laboratory in a sealed container wrapped in a damp cloth to maintain their freshness. Upon arrival, the leaves were promptly wrapped in tin foil and subjected to rapid freezing in liquid nitrogen. Finally, the samples were securely stored in an 80 °C refrigerator.

DNA extraction was performed using a modified CTAB method [21]. The quality of the extracted DNA was assessed through 0.8% agarose gel electrophoresis, while its concentration was quantified using a NanoDrop ND-1000 Nucleic Acid Protein Detector (NanoDrop Technologies Inc., Wilmington, Delaware, USA). For detailed protocols, see Supplementary File S2. Genomic DNA from 42 *C. henryi* samples was randomly fragmented. Desired-length fragments (350 bp, to accommodate PE150 sequencing) were recovered via electrophoresis. A paired-end sequencing library was constructed by ligating sequencing adapters, PCR amplification, and purifying the adapter-ligated fragments. Genome sequencing was performed on each sample using the Illumina NovaSeq PE150 platform.

### 2.4. SNP Detection and Annotation

The sequencing data quality was initially assessed using FastQC (v0.11.5) [22]. Subsequently, adapter sequences and low-quality bases were trimmed from the raw reads using Trimmomatic (v0.36) [23] to generate high-quality clean reads (ILLUMINACLIP:adapters.fa:2:30:10 LEADING:3 TRAILING:3 SLIDINGWINDOW:4:15 MINLEN:20). These clean reads were then aligned to our previously assembled scaffold-level reference genome using BWA-MEM (v0.7.15-r1140) [24,25]. The reference genome sequence and annotation files were obtained from the National Genomics Data Center (NGDC, https://ngdc.cncb.ac.cn, accessed on 19 November 2025) under project accession number PRJCA021809. The resulting alignment files (BAM format) were processed by sorting and indexing with SAMtools (v1.17) [26], followed by duplicate marking using the MarkDuplicates tool from Picard (https://broadinstitute.github.io/picard/, accessed on 20 November 2025). Variant calling was performed for each sample using the HaplotypeCaller module in GATK (v4.2.4.1) [27], with the outputs saved in GVCF format. Individual GVCF files were consolidated, and joint genotyping was conducted to identify SNP variants. The raw SNP set was subjected to quality filtration using the GATK VariantFiltration module, applying the following recommended hard filters: QD < 2.0, FS > 60.0, MQ < 40.0, MQRankSum < −12.5, ReadPosRankSum < −8.0, and SOR > 3.0. The filtered SNPs (Supplementary File S3) were functionally annotated based on the reference genome annotation using ANNOVAR (v2020-06-28) [28]. Finally, the physical distribution of SNPs across the 12 chromosomes was visualized using the ’CMplot’ package in R (v4.4.1). The frequency of SNPs was calculated as the number of SNPs divided by the total length of the corresponding region.

### 2.5. Genetic Diversity Analysis

High-quality SNP data, which were obtained after rigorous quality control, were analyzed for genetic diversity using Plink software (v1.90b6.21) [29]. Key polymorphism parameters, including observed heterozygosity (*Ho*), expected heterozygosity (*He*), polymorphic information content (PIC), and minor allele frequency (MAF) at the loci, were calculated to assess genetic variation. Using VCFtools (v0.1.16) [30], the genomes were partitioned into sliding windows with a size of 500,000 bp and a step size of 50,000 bp (parameters: -*Fst*-window-size 500,000 -*Fst*-window-step 50,000). Based on the window-based calculations, the fixation index (*Fst*) values between subgroups and the total nucleotide diversity (*π*) values within each subgroup were derived. To evaluate the loss of genetic polymorphism in the Taining group relative to the Jian’ou group, the relative nucleotide diversity (ROD) values between the two groups were computed from their respective *π* values. A common window approach was employed, selecting the top 5% of *Fst* values, and the results were integrated and summarized using R scripts.

### 2.6. Divergent Selection Signal Analysis

To investigate signatures of divergent selection between the two groups, candidate genes were identified from genomic windows ranked in the top 5% by *Fst* value. Due to the lack of a genome annotation for *C. henryi*, we established homologous relationships by aligning the protein sequences of these candidate genes against the *Arabidopsis thaliana* proteome (https://www.arabidopsis.org/download_files/Proteins/TAIR10_protein_lists/TAIR10_pep_20101214.fasta.gz, accessed on 14 January 2026). Gene Ontology (GO) and KEGG pathway enrichment analyses were then performed using the ShinyGo platform (http://bioinformatics.sdstate.edu/go82/, accessed on 14 January 2026) to elucidate the functional roles and key biological pathways associated with these genes.

### 2.7. Genetic Structure Analysis

To generate high-quality SNP files for elucidating population structure, we filtered out low-quality SNPs by excluding those with a missingness rate > 2% (--geno 0.02) and a minor allele frequency (MAF) < 3% (--maf 0.03). To further reduce redundancy and potential bias caused by linkage disequilibrium, we performed LD-based pruning using Plink (parameters: --indep-pairwise 50 10 0.2) to obtain a set of independent SNPs for downstream analyses. For principal component analysis (PCA), we employed the smartpca tool from the EIGENSOFT package [31]. Prior to the analysis, we filtered out loci with more than two alleles to ensure the accuracy of the results. To further investigate the population structure, we utilized Plink to generate a ped file, which is a standard input format for many genetic analysis tools. Subsequently, we applied the ADMIXTURE software (v1.3.0) [32,33] to model the genetic structure by presetting K values ranging from 1 to 8 and performing cross-validation. The optimal number of clusters was determined based on the minimum error rate. The results were visualized using R software. Subsequently, we sorted and converted the filtered files into Phylip format, which is commonly used for phylogenetic analysis. Using the neighbor-joining (NJ) method [34] with the Phylip package [35], we constructed a phylogenetic tree, which was then refined and visualized using ITOL platform [36].

## 3. Results

### 3.1. Fruit Traits

**Figure 1:**
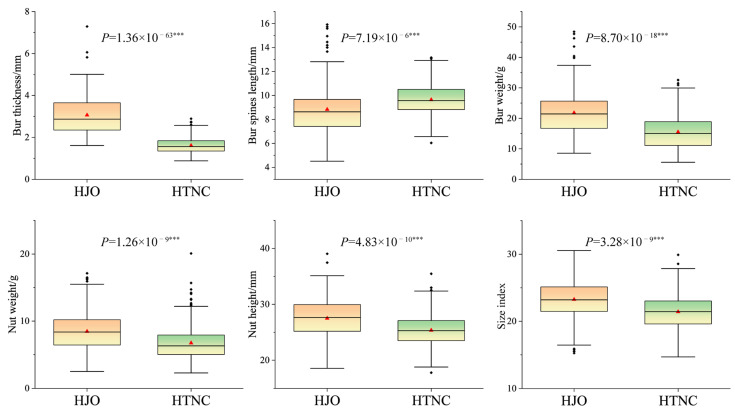
Box plots of fruit traits in 42 *C. henryi* cultivars. The red triangles and solid lines inside the box represent the mean and median values of the traits, respectively, while the black rhombus outside represents outliers. Note: In the figure, the red triangles represent the mean values, the black solid line indicates the median, *** denotes statistically significant differences at a high confidence level (*p* < 0.001), red color indicates *C. henryi* cultivars from the Jian‘ou region, and green color represents cultivars from the Taining region.

The observed values of fruit traits in *C. henryi* cultivars are presented in Figure 1, which clearly illustrates the statistical characteristics of different groups in terms of phenotypic traits, including measures of central tendency (mean, median), dispersion (extremes, interquartile range), and the distribution of outliers. Based on the distribution characteristics of each trait, the HJO group generally exhibited superior phenotypic values compared to the HTNC group. Specifically, the mean and median values for the four fruit traits—bur weight, nut weight, nut height, and size index—were all higher in the HJO group than in the HTNC group. In addition, the HJO group displayed broader interquartile ranges across traits, whereas the HTNC group showed overall narrower interquartile ranges, indicating greater phenotypic variability within the HJO group. Univariate PERMANOVA results revealed that all fruit traits exhibited highly significant differences at the population level (*p* < 0.01). Furthermore, when all traits were combined for multivariate PERMANOVA analysis, the results also indicated that phenotypic differences between groups reached a highly significant level (Supplementary File S4 and Supplementary File S5).

### 3.2. Cluster Analysis

**Figure 2:**
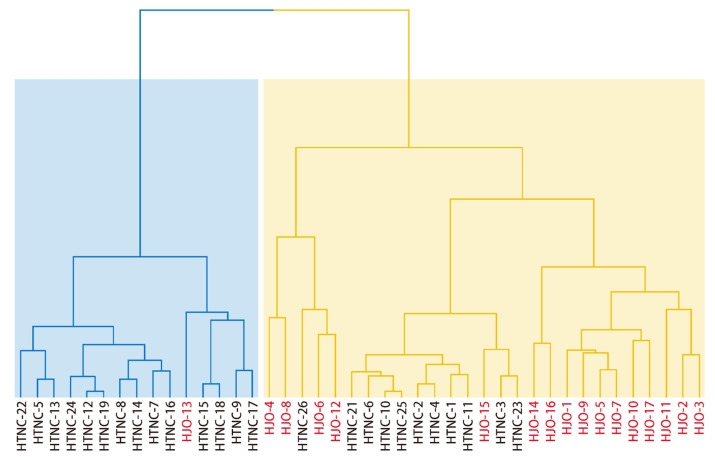
Hierarchical clustering analysis based on Euclidean distance of fruit traits in 42 *C. henryi* cultivars. Cultivars from the Jian’ou region are colored red, and those from the Taining region are colored black. Note: The yellow and blue clusters in the figure represent the two major groups of the 42 *C. henryi* cultivars, as determined by hierarchical clustering based on Euclidean distance.

Based on Euclidean distance, cluster analysis divided the 42 *C. henryi* cultivars into two major groups (Figure 2). The blue group consisted of 15 cultivars, of which 14 (93.3%) originated from the Taining region, with only one Jian’ou cultivar (HJO-13) included. In the cluster analysis, HJO-13 fell into the same branch as the 14 Taining cultivars, indicating a high degree of similarity in the overall characteristics of the measured traits. The yellow group consisted of 27 cultivars, 16 (59.3%) of which were from the Jian’ou region and the remaining 11 from the Taining region. Within the yellow group, several subgroups can be further delineated, among which 11 Jian’ou cultivars form an independent branch, accounting for 68.8% of the Jian’ou cultivars within this group. Overall, these phenotypic clustering results reveal a cross-regional distribution pattern among cultivars from the two regions.

### 3.3. Sequencing Data

The sequencing results are presented in Table 1, with detailed data available in Supplementary File S6. Using the Illumina NovaSeq PE150 platform, we conducted resequencing on 42 *C. henryi* cultivars, generating a total of 2,416,665,746 raw reads, with an average of 57,539,661 raw reads and 8,623,687,972 raw bases per sample. After quality control procedures, which involved the removal of reads containing adapters and low-quality reads, the filtered data averaged 57,026,133 clean reads and 8,403,379,873 clean bases. Furthermore, the proportions of bases meeting the Q20 and Q30 quality standards reached 98.33% and 94.35%, respectively, and the GC content was 36.62%. The alignment results showed that the proportion of filtered reads to the original reads reached 99.08%, and all of the filtered reads met the stringent criteria for subsequent genomic analyses.

### 3.4. Variant Detection and Annotation

**Figure 3:**
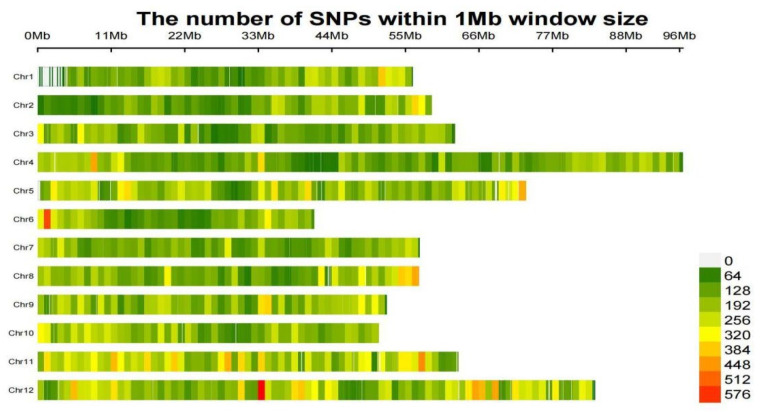
Distribution density of single-nucleotide polymorphisms on chromosomes.

A total of 42 *C. henryi* cultivars were subjected to SNP variation detection, ultimately yielding 6,932,631 SNP loci. According to the annotation results (Table 2 and Supplementary File S7), the majority of SNP loci are situated within intergenic regions, accounting for 3,371,949 sites and representing the largest proportion of the annotated results. SNP loci within intronic regions ranked second in abundance. The SNP density distribution plot (Figure 3) showed a relatively uniform distribution of SNPs across all chromosomes, with the exception of a few regional areas with a higher density. In most regions, the density ranged from 64 to 256 SNPs per 1 Mb interval. Notably, we observed pronounced peaks at specific genomic regions, namely around 1 Mb on chromosome 6 and 33 Mb on chromosome 12, where the SNP density exceeded 576 per 1 Mb. The detailed distribution of SNPs on each chromosome is provided in Supplementary File S8.

### 3.5. Genetic Diversity

The statistical results of key parameters are summarized in Table 3. The analysis revealed that the HJO group exhibited slightly higher genetic diversity than the HTNC group across several indices, including observed heterozygosity (*Ho*), expected heterozygosity (*He*), minor allele frequency (MAF), nucleotide polymorphism (*π*), and polymorphic information content (PIC). The analysis revealed low genetic differentiation between groups, with an *Fst* value of 0.0407 and an ROD value of 0.7297.

### 3.6. Divergent Selection Signal

**Figure 4:**
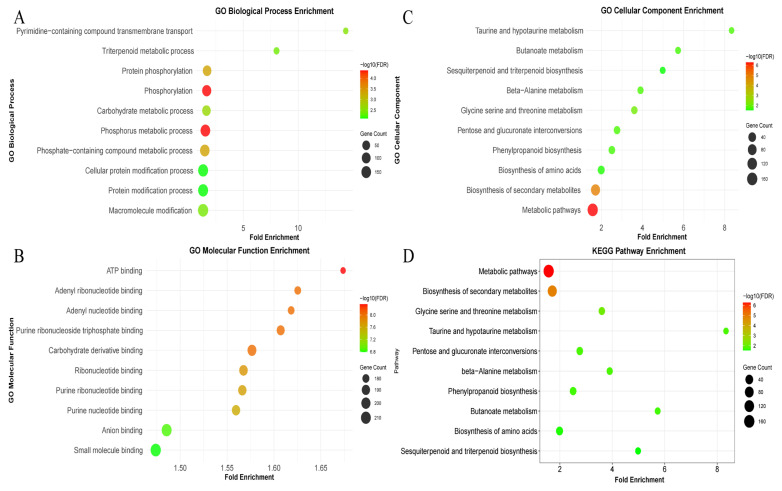
Enriched GO terms and KEGG pathways associated with divergent selection: (**A**): Top 10 enriched GO terms in the Biological Process (BP) ontology. (**B**): Top 10 enriched GO terms in the Molecular Function (MF) ontology. (**C**): Top 10 enriched GO terms in the Cellular Component (CC) ontology. (**D**): Top 10 enriched KEGG pathways for genes under divergent selection.

The analysis revealed that the top 5% *Fst* windows comprised 3129 genomic windows distributed across all 12 chromosomes, encompassing a total of 3363 genes in the reference genome. These genes exhibited homologous relationships with 1205 genes in *Arabidopsis thaliana* (Supplementary File S9). GO enrichment analysis of the 1205 homologous genes (Supplementary File S10) identified 226 terms in the Biological Process (BP) category, of which 75 were significant (FDR < 0.05). In the Molecular Function (MF) category, 235 terms were enriched, including 107 significant terms, while 48 terms were observed in the Cellular Component (CC) category, with 14 being significant. Analysis of the top ten enriched terms (Figure 4) revealed that the significant BP terms were primarily associated with phosphorus metabolic process, phosphorylation, and triterpenoid metabolic process. The predominant MF terms were related to ATP binding, adenyl nucleotide binding, and protein serine kinase activity. The significant CC terms were mainly involved in the plasma membrane, cell periphery, and cytosol. KEGG pathway enrichment analysis of the candidate genes (Supplementary File S11) identified annotations to 14 pathways, with 10 pathways showing significant enrichment (FDR < 0.05). The significantly enriched pathways primarily included “Metabolic pathways,” “Biosynthesis of secondary metabolites,” “Glycine, serine and threonine metabolism,” and “Sesquiterpenoid and triterpenoid biosynthesis.” Notably, the “Sesquiterpenoid and triterpenoid biosynthesis” pathway is consistent with the “triterpenoid metabolic process” identified in the GO enrichment analysis.

### 3.7. Genetic Structure

**Figure 5:**
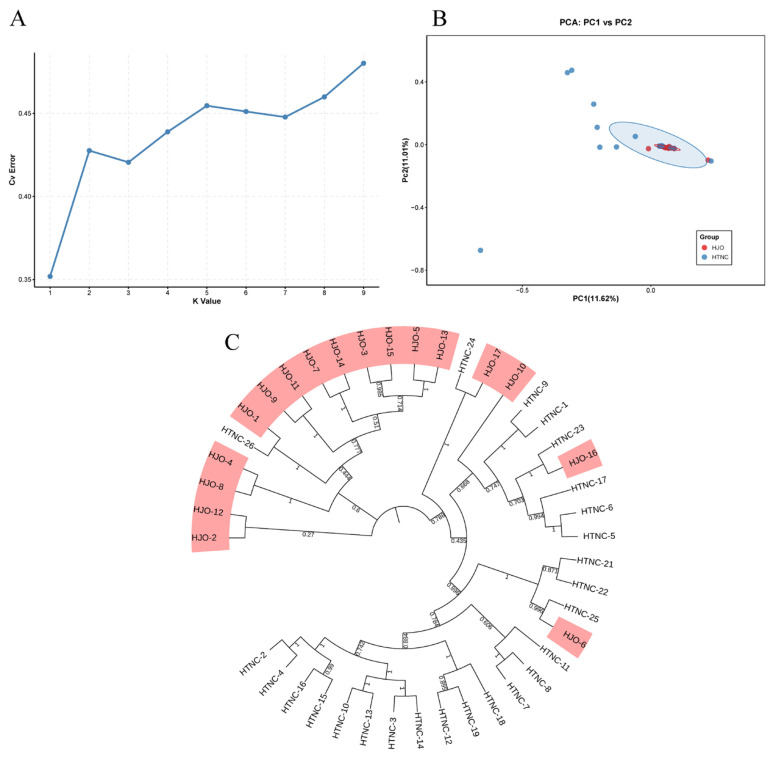
Genetic structure analysis of 42 *C. henryi* cultivars. (**A**): Cross-validation error rates for different K values (1–9). (**B**): Scatter plot of the first two principal components (PC1 vs. PC2). (**C**): phylogenetic trees of the 42 *C. henryi* cultivars. Note: The colored labels in the figure represent cultivated varieties of *C. henryi* from different geographical origins.

Based on the filtered high-quality SNPs, this study conducted a genetic structure analysis on 42 main *C. henryi* cultivars originating from HTNC and HJO. The results were visualized to illustrate the genetic relationships among these cultivars; the detailed results are shown in Figure 5. To explore population structure, the ADMIXTURE software (v1.3.0) was utilized, with the number of clusters (K) ranging from one to nine. The results showed that the cross-validation (CV) error reached its minimum at K = 1. This indicated optimal population division and suggested a high degree of genetic consistency between the HTNC and HJO. As the K-value increased, the CV error showed a gradual rise. Meanwhile, principal component analysis (PCA) revealed that, except for a few outliers, most individuals clustered relatively closely. Specifically, individuals from the HJO group were more concentrated in the genetic space, while those from the HTNC group exhibited a relatively dispersed distribution. This pattern was further supported by the neighbor-joining (NJ) phylogenetic tree, which did not reveal clear geographically based clustering. Instead, the tree consisted of several small, admixed clades.

## 4. Discussion

Phenotypic trait analysis provides an empirical foundation for elucidating genetic architecture and clarifying mechanisms of domestication and differentiation by systematically quantifying variation arising from genotype-by-environment interactions [37]. In this study, the Jian’ou group of *C. henryi* exhibited higher mean and median values for most traits compared to the Taining group, along with broader interquartile ranges. This indicates greater variability within the Jian’ou group and suggests a higher potential for individuals with superior trait expression. Extensive phenotypic variation often corresponds to diverse genetic variation, thereby providing a broad foundation for the selection of target traits associated with high yield, superior quality, and stress resistance. In contrast, the Taining group generally exhibited narrower interquartile ranges across traits, indicating more concentrated phenotypic expression and lower variability. This suggests a relatively high degree of genetic homogenization in this germplasm resource, which may result from long-term directional selection or limited germplasm exchange. Although this genetic homogeneity maintains high stability in phenotypic traits, it may also lead to the loss of the population’s ability to cope with stress [38]. Furthermore, phenotypic cluster analysis revealed that the clustering pattern of *C. henryi* cultivars was not strictly constrained by geographical boundaries, as some cultivars from distinct origins exhibited phenotypic convergence within the same clusters. Therefore, in the breeding of superior *C. henryi* cultivars, efforts should not be confined to local germplasm resources. It is advisable to actively utilize phenotypically convergent cultivars from across geographical origins to identify genetic loci associated with their shared desirable traits. Simultaneously, cultivars that originate from one geographic region but cluster phenotypically with those from another region should be conserved and utilized as unique germplasm resources. This will provide essential material support for broadening the genetic base of chestnut breeding programs.

SNP molecular markers, as the most common form of genetic variation in genomes, are one of the key subjects of resequencing data analysis. Currently, this method is widely applied in areas such as germplasm resource evolution, phylogenetic analysis, population structure analysis, and genetic diversity analysis [39,40]. In this study, a total of 6,932,631 high-quality SNPs were identified from 42 accessions of *C. henryi*. The annotation results revealed that the intergenic region contained the largest number of SNPs, accounting for 48.64% of the total number. In early breeding research, due to technological limitations, breeders found it difficult to conduct in-depth investigations into intergenic regions; therefore, the focus of breeding efforts often centered on genes encoding proteins. In recent years, thanks to the rapid development of technology, the significance of intergenic regions has gradually become apparent. Numerous studies have shown that intergenic regions contain a large number of regulatory elements that are core components of gene regulatory networks and, together with coding regions, participate in processes such as chromatin dynamics, environmental adaptation, and species evolution [41,42]. The findings of Keke X et al. indicate that regulatory elements, such as enhancers located in intergenic regions, can drive transcriptional differences in epidermal cell subtypes through spatial interactions, thereby revealing the developmental trajectories of vascular cells and guard cells in *Arabidopsis thaliana* [43]. A research team from Huazhong Agricultural University discovered that KRN4, which is located in the intergenic region of maize, is in an open chromatin state and possesses enhancer activity. It can recruit the transcription factors UB2 and OBF family proteins to remotely regulate the expression of the target gene UB3, thereby influencing the development of the female spike meristem and leading to variations in spike row number [44]. Therefore, future studies should conduct more in-depth exploratory analyses targeting SNP loci in the intergenic regions of *C. henryi*, elucidating the regulatory elements and protein interaction patterns from the perspective of intergenic regions, and building a deeper understanding of gene expression regulatory networks, thereby providing a solid theoretical foundation for the further development of breeding research.

Genetic diversity serves as the cornerstone of biodiversity, determining a species’ adaptability, evolutionary potential, and ecological functions, and is therefore crucial for species survival and ecosystem stability [45,46]. The reduced *Ho* and *He* levels may result from inbreeding or non-random mating, thus producing more homozygous and fewer heterozygous individuals, or from strong selection pressures favoring specific genotypes and promoting genetic [47] homogeneity. The low MAF could be attributed to a small population size and genetic drift [48], which increase the likelihood of losing rare alleles, coupled with a slow rate of new allele production and insufficient gene influx. Based on resequencing analyses of the *C. henryi* groups from Jian’ou and Taining, this study found that the *Fst* value between the two cultivated groups was 0.0407—significantly lower than the values reported by Dane F. et al. for three other *Castanea* populations [49]. This relatively low *Fst* value indicates a moderate level of genetic differentiation between the Taining and Jian’ou groups and suggests that they retain substantial genetic similarity. Furthermore, the observed heterozygosity (*Ho*) in both groups was lower than the expected heterozygosity (*He*). This suggests possible inbreeding or similar selective pressures in these groups, leading to an increased frequency of homozygous individuals and a relative decrease in heterozygosity. Further comparison showed that the Taining group had slightly lower values than the Jian’ou group across parameters including *Ho*, *He*, MAF, *π*, and PIC, suggesting a decline in its genetic diversity [50]. We hypothesize that Jian’ou represents an older or more diverse cultivation center, where its germplasm resources retain greater ancestral genetic variation, thereby providing a broader basis for selection. Based on the integration of genetic diversity and phenotypic trait analyses, the Jian’ou group is better suited as a core repository of elite germplasm for selecting high-performance individuals as parents in targeted breeding for breakthrough cultivars. In contrast, the Taining group may serve as a stable backbone parent to maintain trait consistency in hybrids or in standardized cultivation. Therefore, to mitigate its genetic diversity loss, hybridization with introduced germplasm could be employed to enrich its genetic background and enhance breeding potential.

This study employed a genome-wide *Fst* scan to identify the top 5% of genomic regions with the strongest genetic differentiation as candidate selective sweeps, thereby elucidating the functional genomic basis underlying the divergence among 42 cultivated varieties of *C. henryi*. Integrated GO and KEGG analyses revealed that the genetic divergence between the HJO and HTNC groups converges on a coordinated gene network regulating core energy metabolism, biosynthesis of secondary defense compounds, and cellular structure development. The most prominent signal originated from secondary metabolism, particularly the triterpenoid and phenylpropanoid biosynthesis pathways. Triterpenoids are crucial phytochemicals involved in plant resistance against insects, pathogens, and environmental stresses [51]. We therefore propose that the observed divergence in these pathways likely reflects divergent historical pressures from pests and pathogens, resulting in varied selection regimes during breeding in the two regions. The analysis also revealed the enrichment of functional terms and pathways such as ATP binding (MF), protein phosphorylation (BP), and glycine/serine metabolism (KEGG), indicating that selective pressures have acted on core regulatory mechanisms of cellular energy and resource allocation. Glycine and serine metabolism functions as a hub connecting photorespiration [52], C1 metabolism [53], and nucleotide synthesis [54]; their differentiation likely reflects genetic differences in the allocation strategies for photosynthetically fixed carbon between the two groups. In combination with phenotypic data, fruits from the HJO group generally exhibited greater weight and size than those from HTNC, suggesting that the HJO group may allocate more resources toward starch and sucrose synthesis to support larger fruit biomass.

Genetic structure analysis serves as a powerful tool for elucidating the mechanisms underlying the formation and maintenance of genetic diversity within populations [55]. In this study, high-quality SNP markers from 42 *C. henryi* accessions were employed to investigate their genetic structure. The analysis indicated that the optimal number of genetic clusters was K = 1. This result does not support the division of the *C. henryi* germplasm into two or more discrete genetic groups, demonstrating a highly similar genetic composition between the Jian’ou and Taining groups, which suggests that these two groups may share a common ancestral origin. Consistent with this result, both PCA and NJ tree analysis revealed no clear large-scale group differentiation corresponding to geographical origins. This genetic structural feature corroborates the phenotypic clustering results, jointly indicating that although a certain geographic pattern exists, the clustering is not strictly confined by geographic boundaries, but rather exhibits cross-geographic clustering characteristics. However, compared to the results of genetic structure analysis, the clustering based on phenotypic traits exhibits relatively distinct group delineations. This observation may be attributed to the fact that the variation and distribution of phenotypic traits result from the combined effects of genetic background, environmental adaptation, and artificial directional selection. Given the subtle environmental differences between the habitats of the two groups, it can be inferred that although genetic differentiation between the two groups is limited at the genomic level, factors such as local environmental conditions, phenotypic plasticity, or adaptive phenotypic divergence may have influenced the expression of related traits, thereby leading to discernible inter-group differences at the phenotypic level [56].

Genetic diversity is crucial for species to adapt to environmental changes. The loss of genetic diversity reduces a species’ ability to cope with new environments, climate change, and emerging diseases [57]. The results of this study indicate that the genetic diversity of the main *C. henryi* cultivars in Fujian Province has already reached a relatively low level, making the development of relevant conservation strategies critical. Such strategies should be based on the genetic characteristics, ecological requirements, and existing risk factors of the *C. henryi* cultivars to ensure their sustainable reproduction and maintain their ecological and economic value [58,59]. To this end, the following systematic measures are proposed for the conservation of *C. henryi* genetic resources. First, establish in situ conservation areas and germplasm resource banks to achieve dual protection by minimizing disturbances and enabling systematic preservation. Second, conduct artificial pollination and directional hybridization to promote gene flow and the introduction of desirable traits. Third, employ molecular marker technology for continuous monitoring of genetic diversity dynamics, thereby guiding scientific conservation efforts. Through these integrated measures, the genetic integrity and sustainable utilization of *C. henryi* can be effectively safeguarded.

At present, the majority of *C. henryi* cultivars originate from the Taining and Jian’ou regions. This study systematically selected 42 cultivated varieties that cover the mainstream germplasm from these two major production areas, thereby providing solid justification for the representativeness of the core breeding materials. However, several limitations of this study warrant attention. First, relying solely on 42 cultivated materials from these two regions may not fully capture the genetic diversity characteristics of broader *C. henryi* populations. Second, the exclusive use of clonally propagated materials may lead to homogenization of genomic backgrounds, potentially affecting the accuracy of the whole population genetic structure analyses. Third, although functional enrichment results (GO and KEGG) were obtained from genes within the top 5% *Fst* windows, the generally low *Fst* values across the genome suggest that these signals likely reflect localized or subtle adaptive divergence rather than strong, genome-wide selective sweeps. Furthermore, the relatively low sequencing depth in this study limits the detection sensitivity for low-frequency variants and rare alleles, which could compromise the completeness of subsequent selective sweep analysis and functional gene mining.

Based on the findings and limitations of this study, future research should focus on deepening efforts in the following five areas: First, it is essential to expand the scope of germplasm collection by systematically incorporating wild populations, landraces, and cultivated materials from diverse geographical origins, thereby establishing a more representative *C. henryi* germplasm resource bank. Second, the integration of high-depth whole-genome sequencing technologies is recommended to enhance the accuracy and comprehensiveness of genetic variation detection. Third, by combining multi-omics data, such as transcriptomics and epigenomics, a deeper exploration of the genetic basis underlying key agronomic traits in *C. henryi* can be achieved. Fourth, conducting cross-regional population comparative studies will help systematically elucidate the domestication history and dissemination pathways of *C. henryi*. Finally, the development of a comprehensive phenotype-genotype association database will provide theoretical support for molecular marker-assisted breeding and gene editing breeding. These efforts will offer a more reliable theoretical foundation for the conservation and genetic improvement of *C. henryi* germplasm resources.

## Figures and Tables

**Table 1:** Sequencing results of 42 cultivated *C. henryi* varieties.

Sample	Mean Depth	Mapped Rate	Duplicate Rate	Total Reads	Q20	Q30	GC
42	10X	97.66%	19.55%	2,395,105,896	98.33%	94.35%	36.62%

**Table 2:** Annotation information of single-nucleotide polymorphisms.

Variant Type of SNPs	SNPs	Frequency (%)
3′UTR region mutation	7045	0.10
5′UTR region mutation	8125	0.12
Upstream mutations in genes	421,847	6.08
Downstream mutations in genes	369,935	5.34
Involves both upstream and downstream regions	34,406	0.50
Intronic region	637,385	9.19
Exon region	147,843	2.13
Intergenic region	3,371,949	48.64
Splice site region	1492	0.02

**Table 3:** Parameters of genetic polymorphism in major cultivated populations of *C. henryi*.

Group	*Ho*	*He*	*π*	MAF	*Fst*	PIC	ROD
HJO	0.0275	0.0777	0.0218	0.0420	0.0407	0.0725	0.7297
HTNC	0.0194	0.0668	0.0186	0.0353		0.0636	

## Data Availability

The genome resequencing data are available in the Genome Sequence Archive (GSA) of the NGDC (National Genomics Data Center) under accession number PRJCA040541. For further inquiries, please contact the corresponding author (Yu Li) directly.

## Supplementary Materials

The following supporting information can be downloaded at: https://www.mdpi.com/article/10.3390/cimb48020173/s1.
