# Supplementary material for: Genetic Variation in the Main Cultivar Collection of Castanea henryi Revealed by Genome Resequencing
Source: Curr Issues Mol Biol. 2026 Feb 3;48(2):173. doi: 10.3390/cimb48020173 (PMC12940070; doi:10.3390/cimb48020173)
Supplement: Supplementary file 1 [file cimb-48-00173-s001.zip › File S12 Figure/File S10 Figure/Origin Figure/Figure.4-A.pdf]

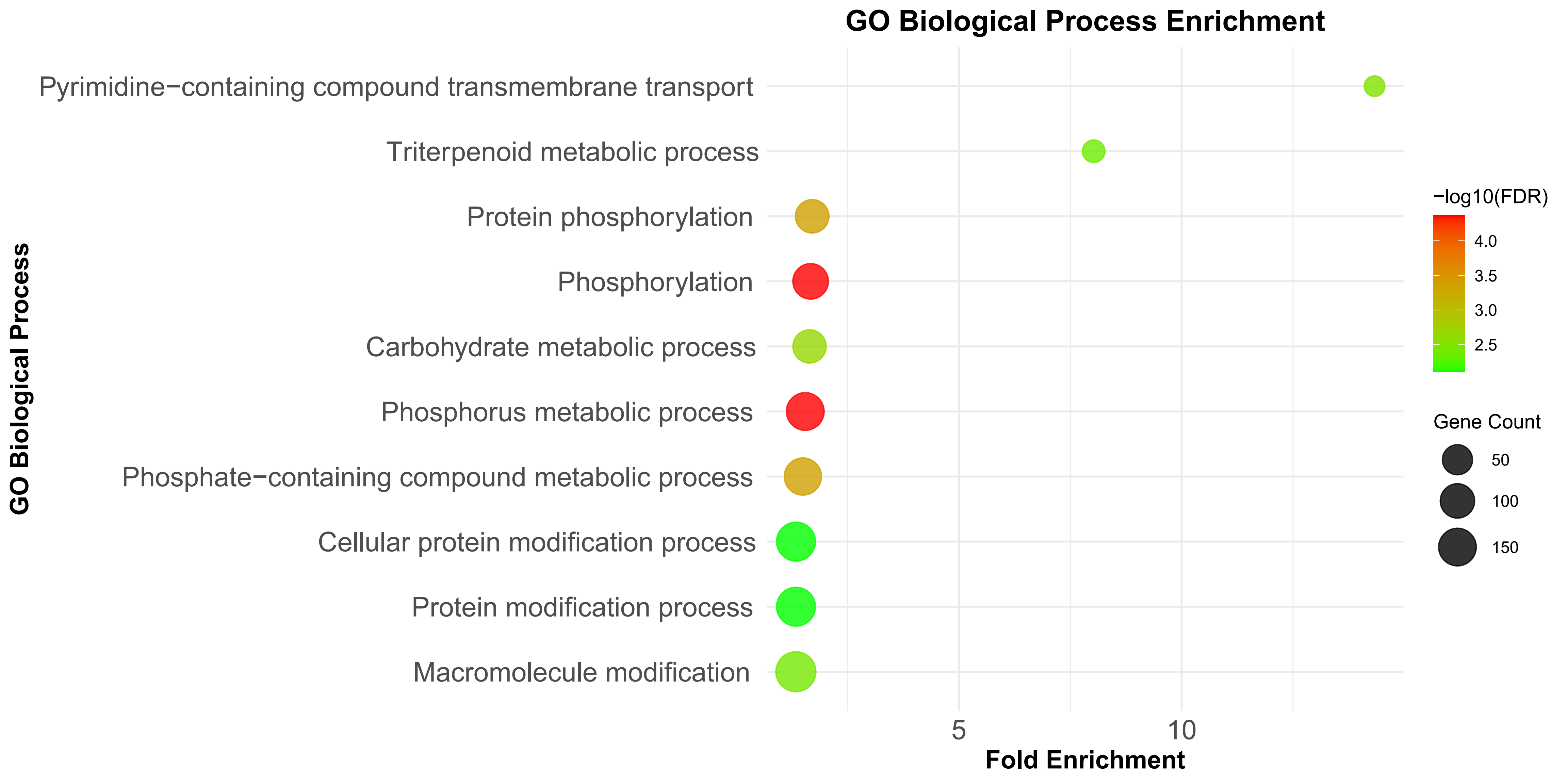
GO Biological Process Enrichment
Pyrimidine−containing compound transmembrane transport
Triterpenoid metabolic process
Protein phosphorylation
Phosphorylation
Carbohydrate metabolic process
Phosphorus metabolic process
Phosphate−containing compound metabolic process
Cellular protein modification process
Protein modification process
Macromolecule modification
GO Biological Process
5
10
Fold Enrichment
−log10(FDR)
4.0
3.5
3.0
2.5
Gene Count
50
100
150
