# Supplementary material for: Genetic Variation in the Main Cultivar Collection of Castanea henryi Revealed by Genome Resequencing
Source: Curr Issues Mol Biol. 2026 Feb 3;48(2):173. doi: 10.3390/cimb48020173 (PMC12940070; doi:10.3390/cimb48020173)
Supplement: Supplementary file 1 [file cimb-48-00173-s001.zip › File S2 Detailed Protocol for DNA Extraction.pdf]

The detailed protocol for DNA extraction is as follows:

1. Approximately 20 mg of dried leaf tissue was weighed using an analytical balance, ground to a fine powder in liquid nitrogen with a mortar and pestle, and transferred into a microcentrifuge tube. Then, 400 μL of Buffer LP1 and 6 μL of RNase A (10 mg/mL) were added, followed by vortexing for 1 min and incubation at room temperature for 10 min to ensure complete lysis.

2. After adding 130 μL of Buffer LP2 and vortexing for 1 min, the mixture was centrifuged at 12,000 rpm for 5 min in a refrigerated centrifuge. The supernatant was carefully transferred to a new tube.

3. A volume of Buffer LP3 equal to 1.5 times the supernatant volume was added and mixed gently by pipetting. About 500 μL of the mixture (loaded in two steps if necessary) was applied to a spin column placed in a collection tube and centrifuged at 12,000 rpm for 1 min. The flow-through was discarded and the spin column was returned to the collection tube.

4. The column was washed twice with 500 μL of Buffer GW2, each time centrifuged at 12,000 rpm for 1 min, and the flow-through was discarded.

5. The column was centrifuged again at 12,000 rpm for 2 min to remove residual wash buffer, then air-dried at room temperature for several minutes.

6. Finally, the spin column was transferred to a clean microcentrifuge tube, 100 μL of Buffer GE was added directly onto the membrane, and after 5 min of incubation at room temperature, DNA was eluted by centrifugation at 12,000 rpm for 1 min.

All buffers (LP1, LP2, LP3, GW2, GE), RNase A, spin columns, and collection tubes were provided by Beijing ComWin Biotech Co., Ltd., China.

DNA concentration was measured using a NanoDrop ND-1000 spectrophotometer (NanoDrop Technologies Inc., USA), and DNA integrity was assessed by 0.8 % agarose gel electrophoresis.
