# Supplementary figures and images for: Genetic Variation in the Main Cultivar Collection of Castanea henryi Revealed by Genome Resequencing
Source: Curr Issues Mol Biol. 2026 Feb 3;48(2):173. doi: 10.3390/cimb48020173 (PMC12940070; doi:10.3390/cimb48020173)

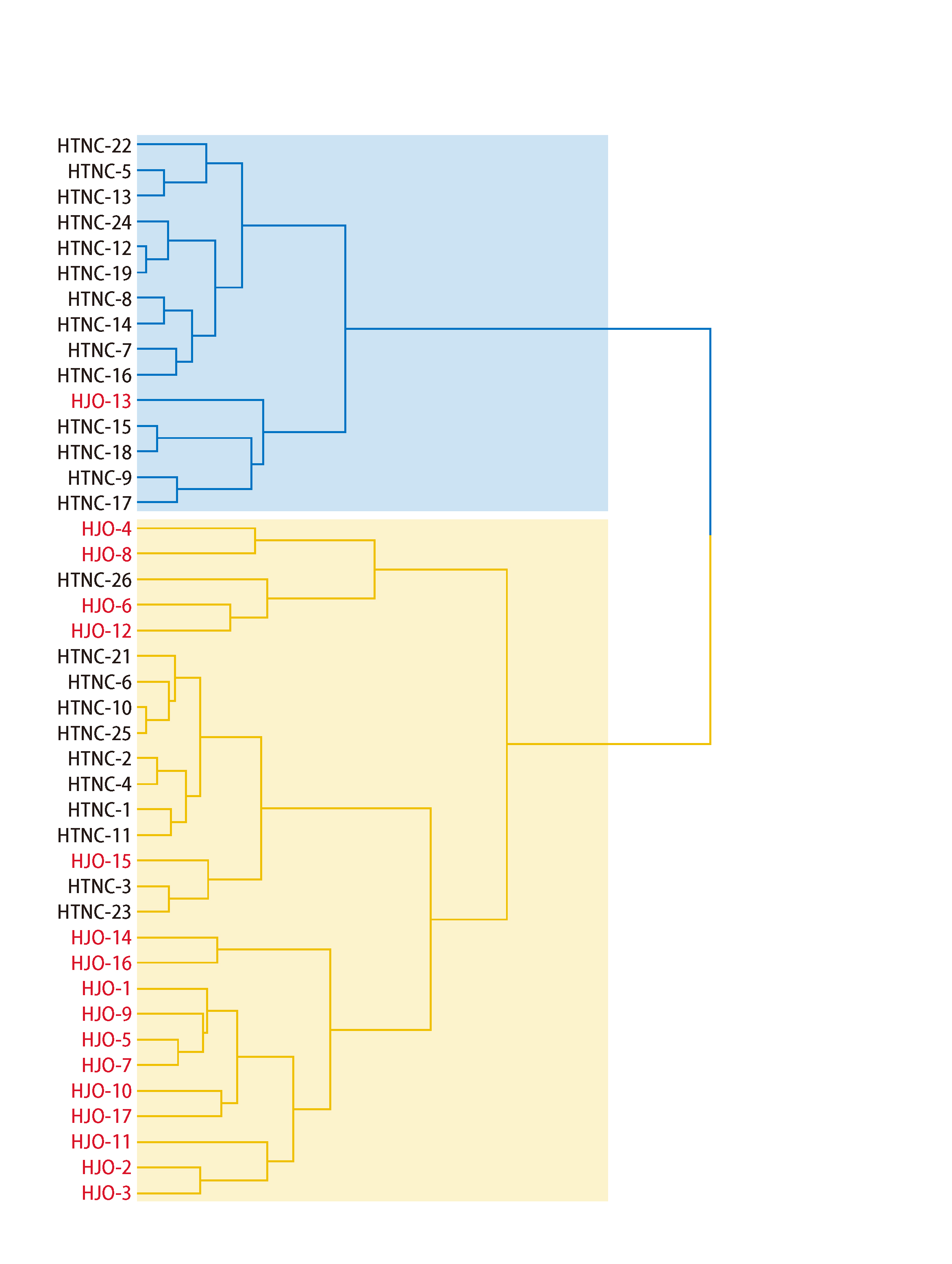

Supplement: Supplementary file 1 [file cimb-48-00173-s001.zip › File S12 Figure/File S10 Figure/Figure.2.jpg]

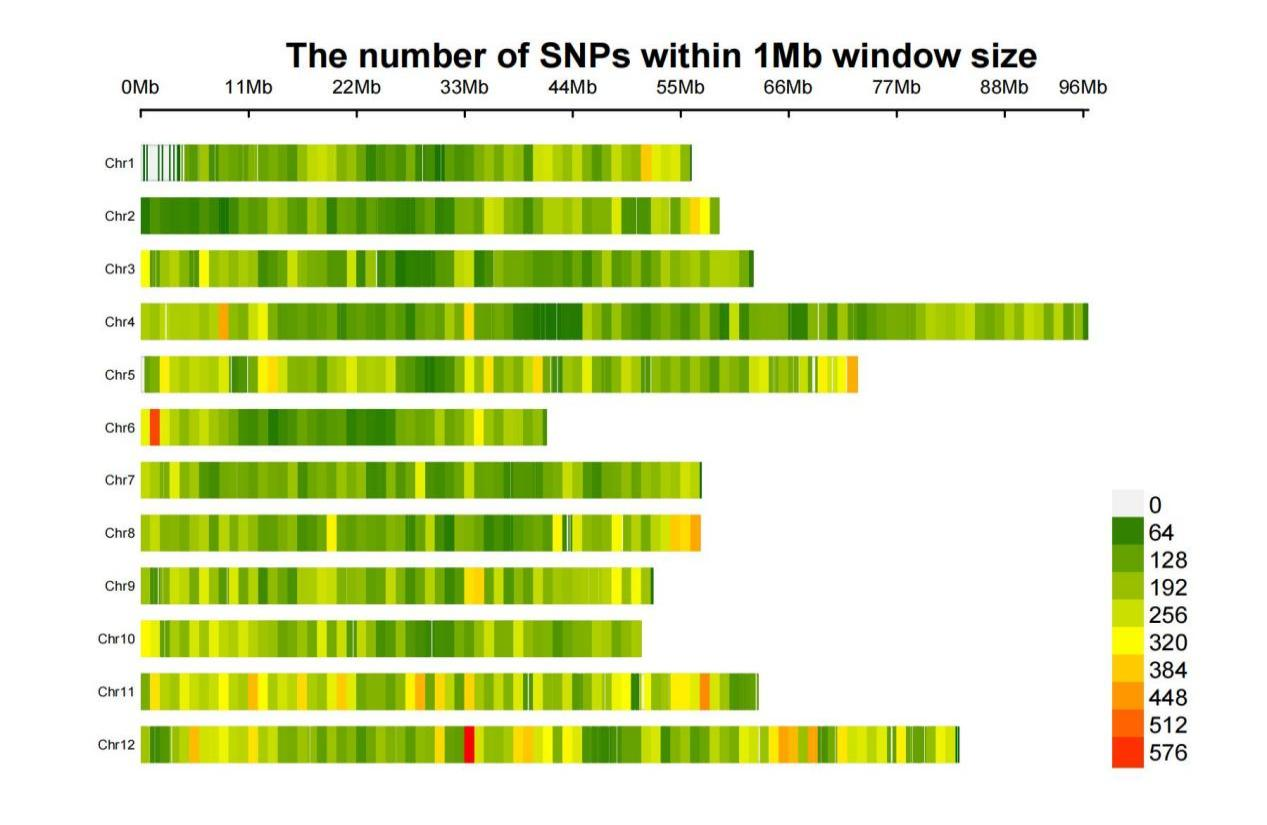

Supplement: Supplementary file 1 [file cimb-48-00173-s001.zip › File S12 Figure/File S10 Figure/Figure.3.png]

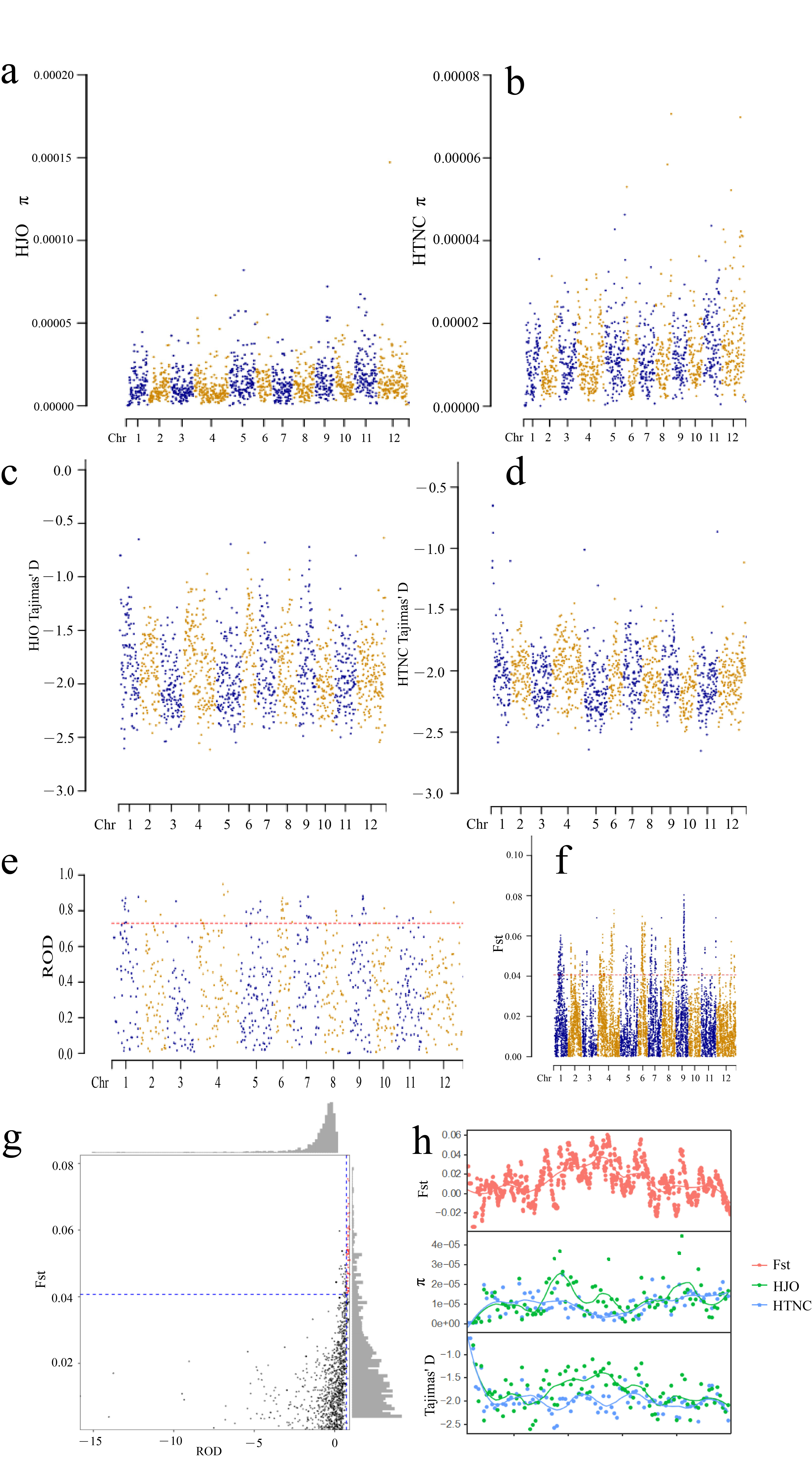

Supplement: Supplementary file 1 [file cimb-48-00173-s001.zip › File S12 Figure/File S10 Figure/Genetic diversity.jpg]

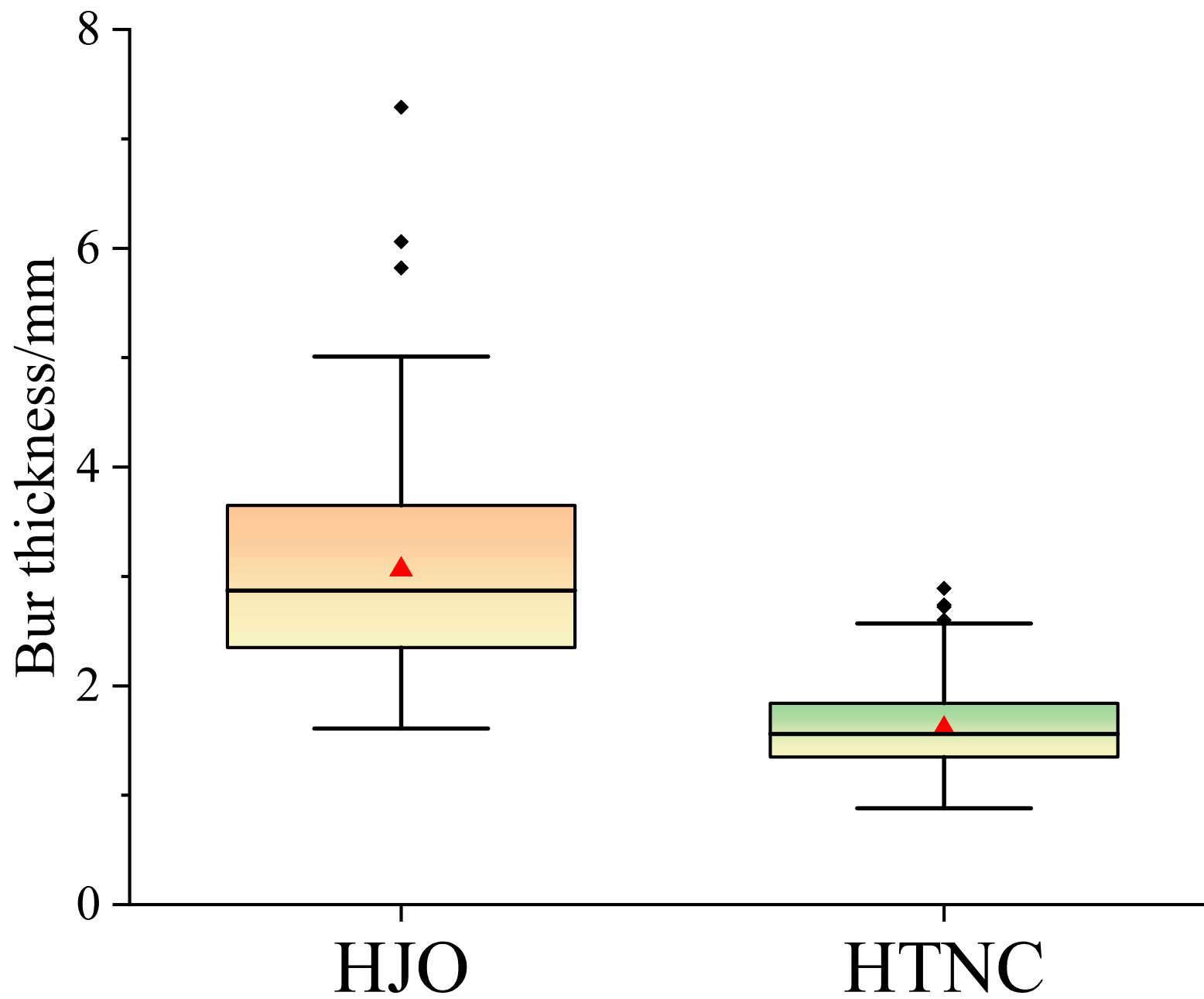

Supplement: Supplementary file 1 [file cimb-48-00173-s001.zip › File S12 Figure/File S10 Figure/Origin Figure/Figure.1-1.pdf]

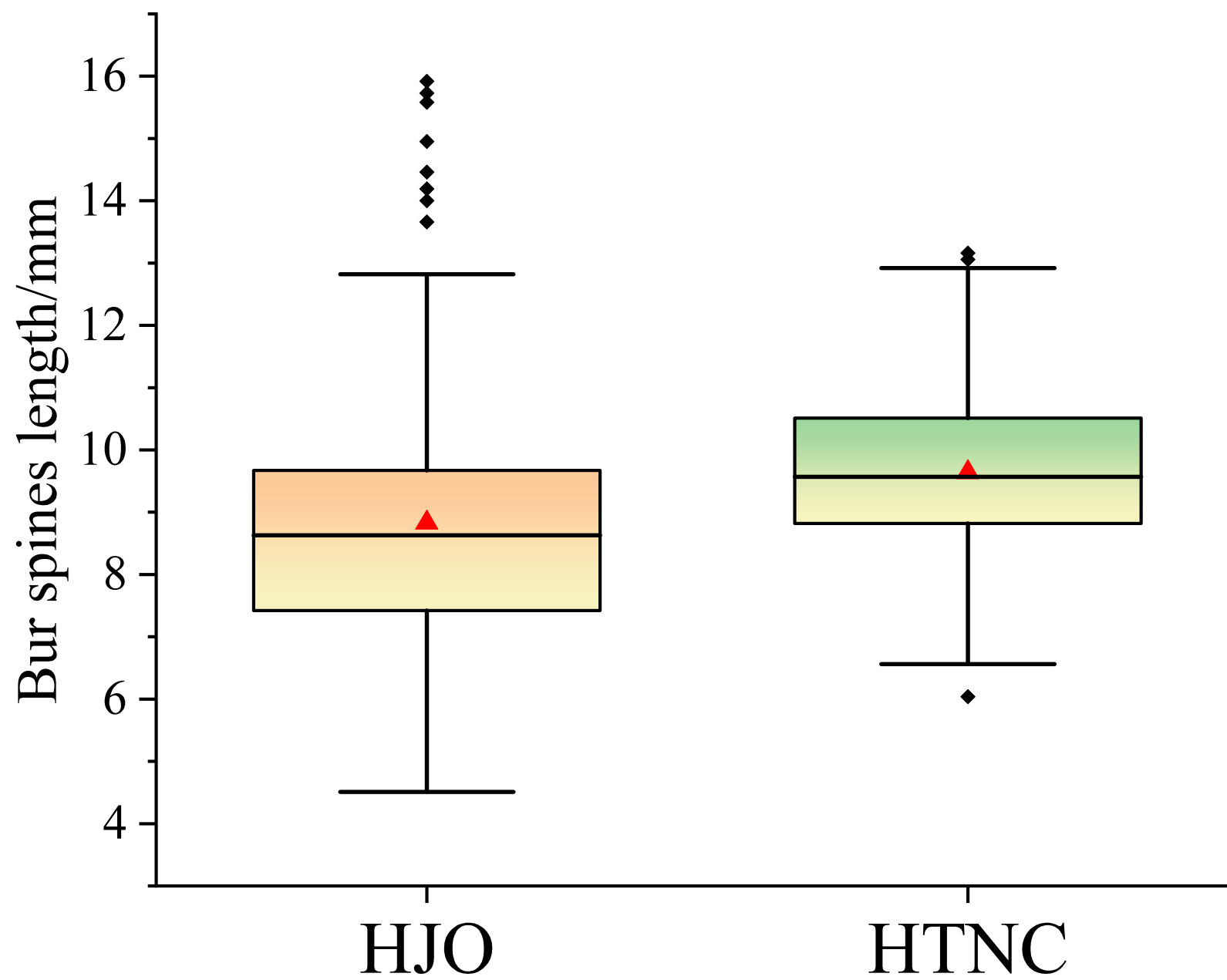

Supplement: Supplementary file 1 [file cimb-48-00173-s001.zip › File S12 Figure/File S10 Figure/Origin Figure/Figure.1-2.pdf]

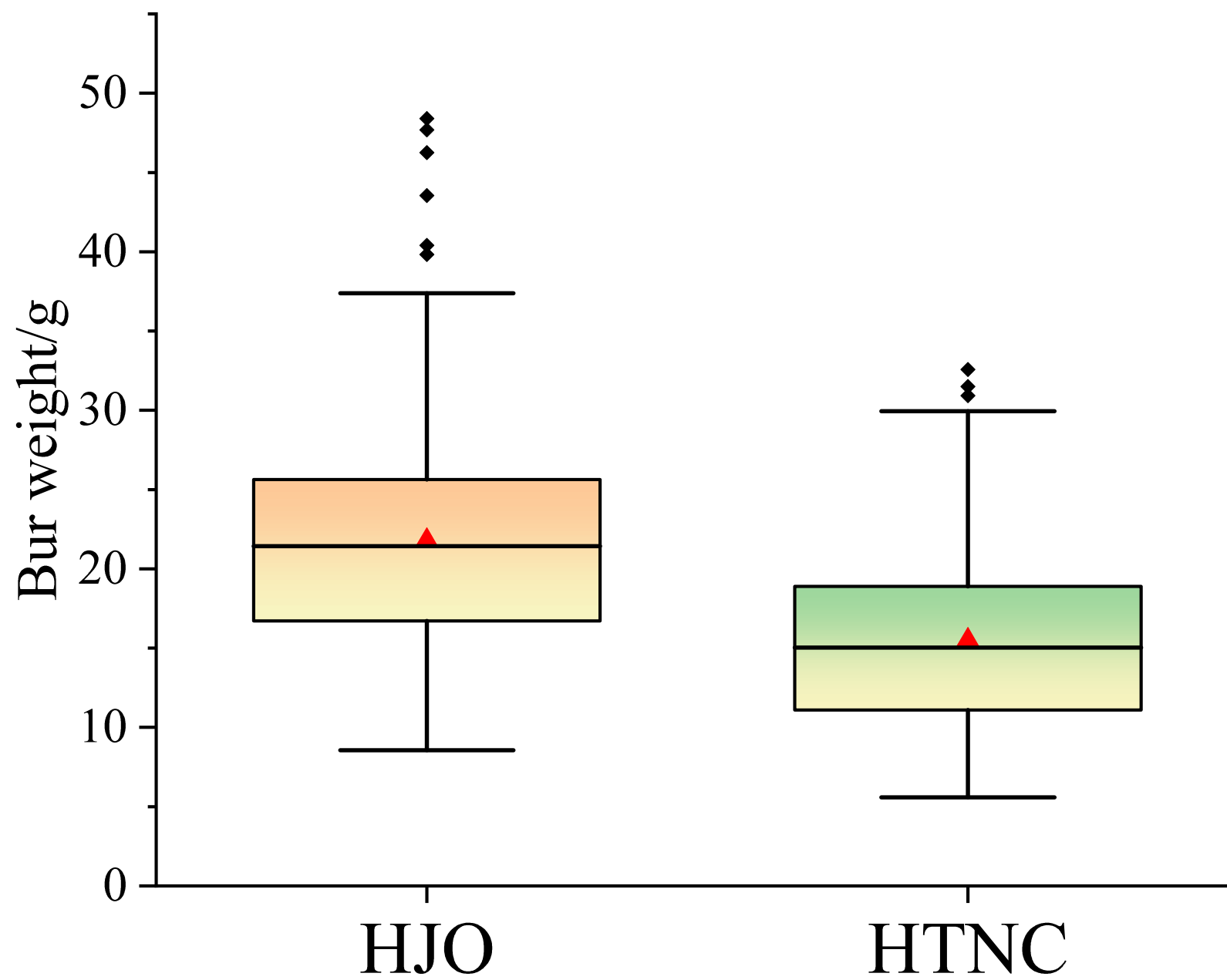

Supplement: Supplementary file 1 [file cimb-48-00173-s001.zip › File S12 Figure/File S10 Figure/Origin Figure/Figure.1-3.pdf]

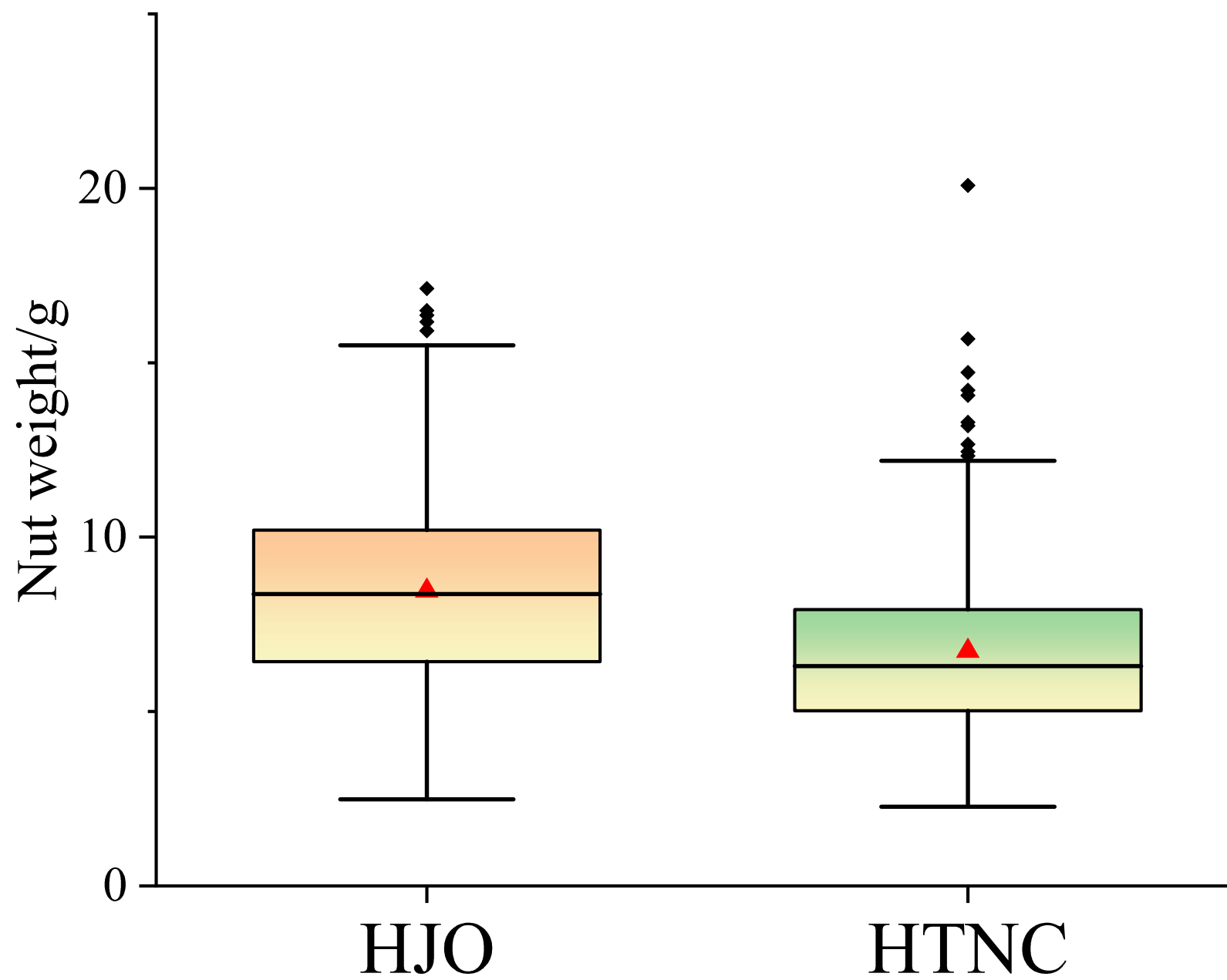

Supplement: Supplementary file 1 [file cimb-48-00173-s001.zip › File S12 Figure/File S10 Figure/Origin Figure/Figure.1-4.pdf]

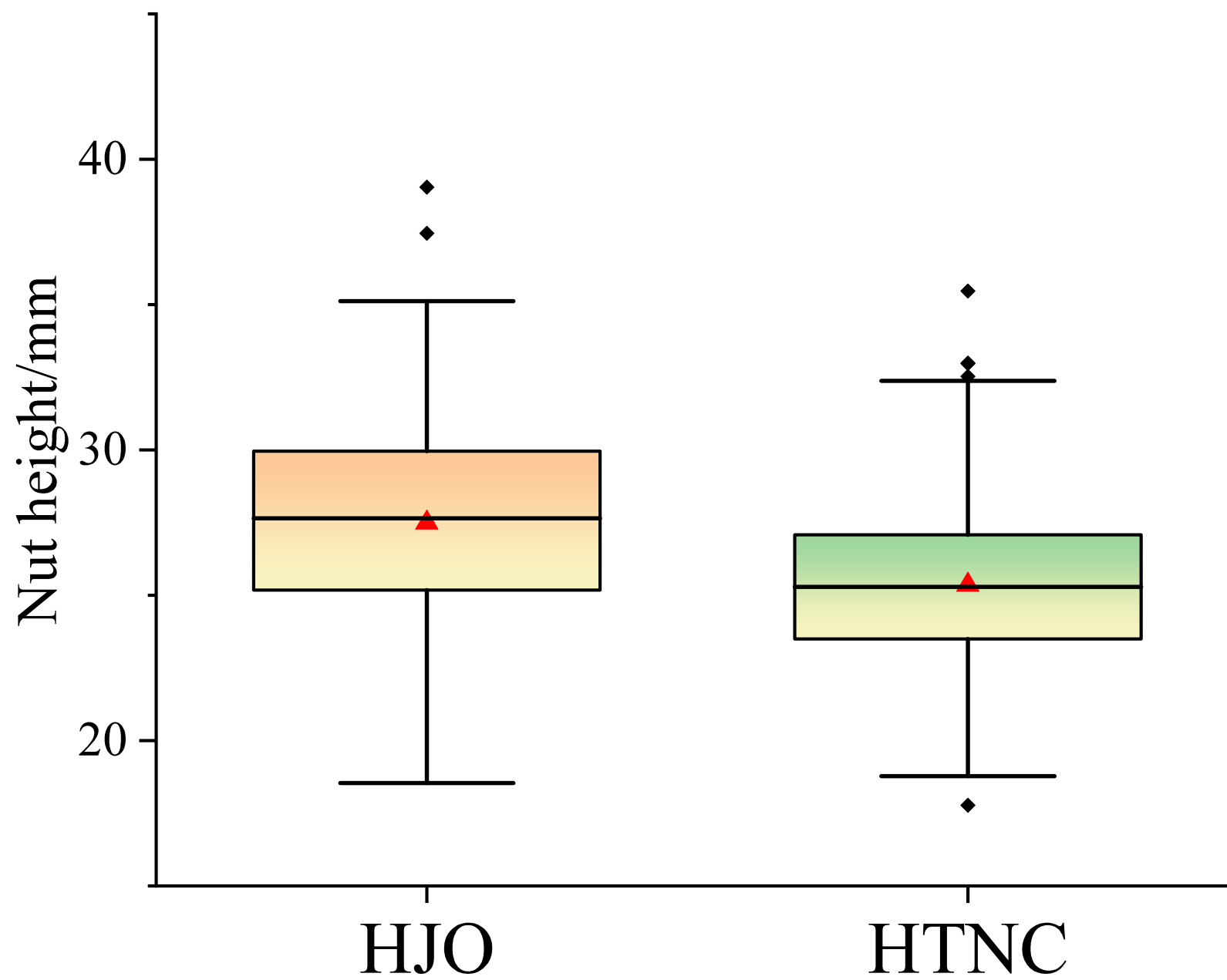

Supplement: Supplementary file 1 [file cimb-48-00173-s001.zip › File S12 Figure/File S10 Figure/Origin Figure/Figure.1-5.pdf]

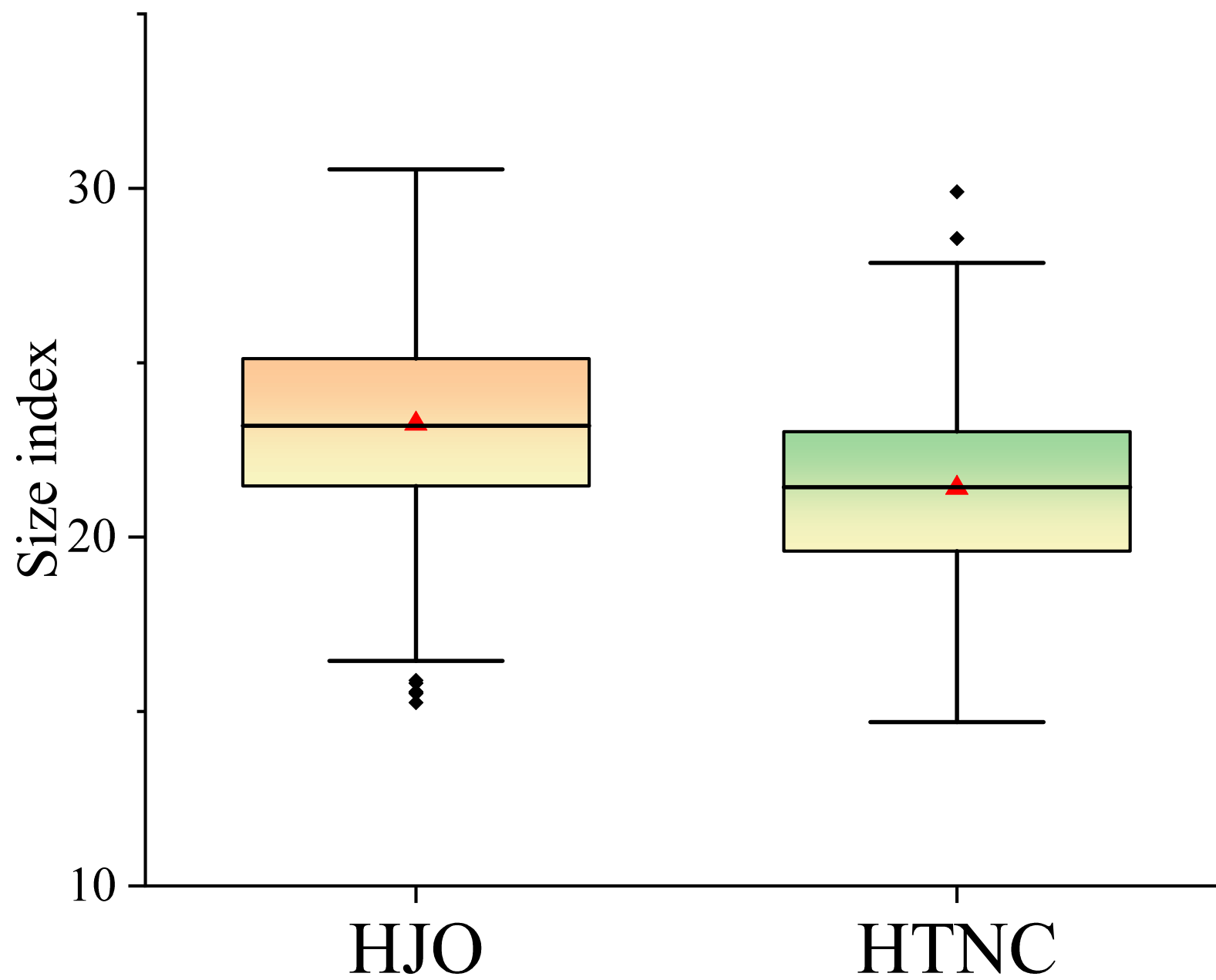

Supplement: Supplementary file 1 [file cimb-48-00173-s001.zip › File S12 Figure/File S10 Figure/Origin Figure/Figure.1-6.pdf]

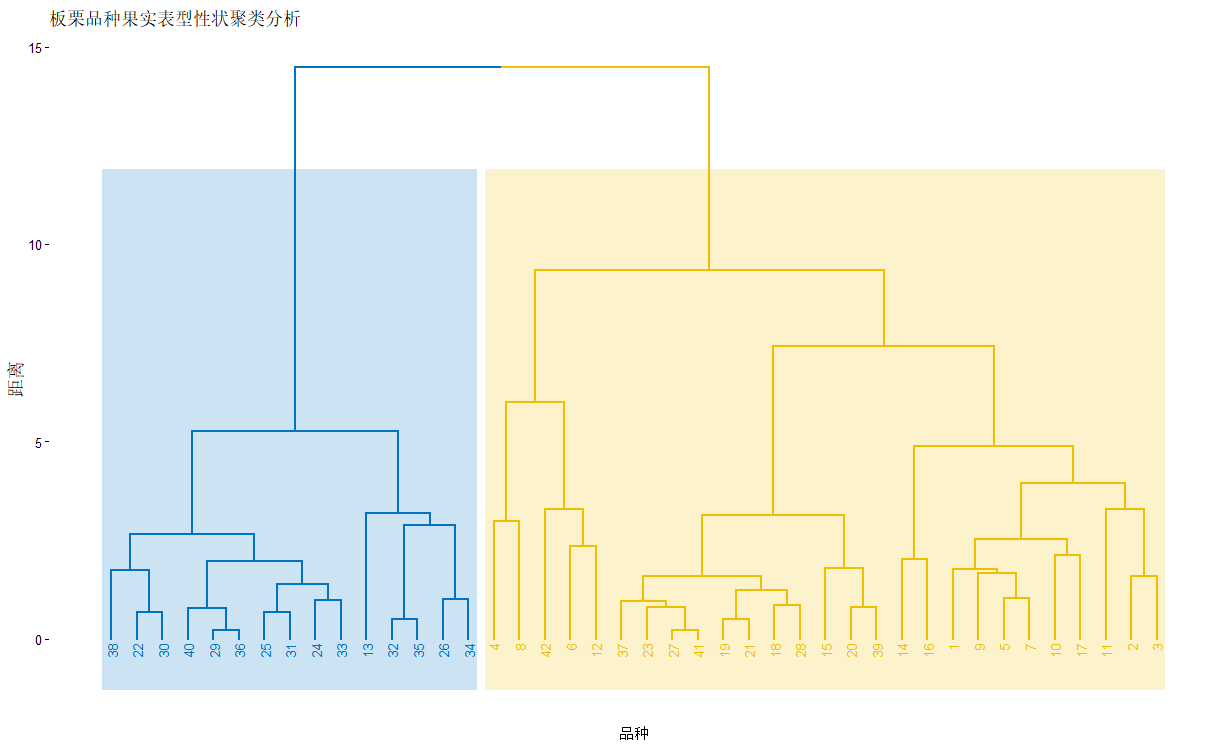

Supplement: Supplementary file 1 [file cimb-48-00173-s001.zip › File S12 Figure/File S10 Figure/Origin Figure/Figure.2.png]

# GO Molecular Function Enrichment

GO Molecular Function

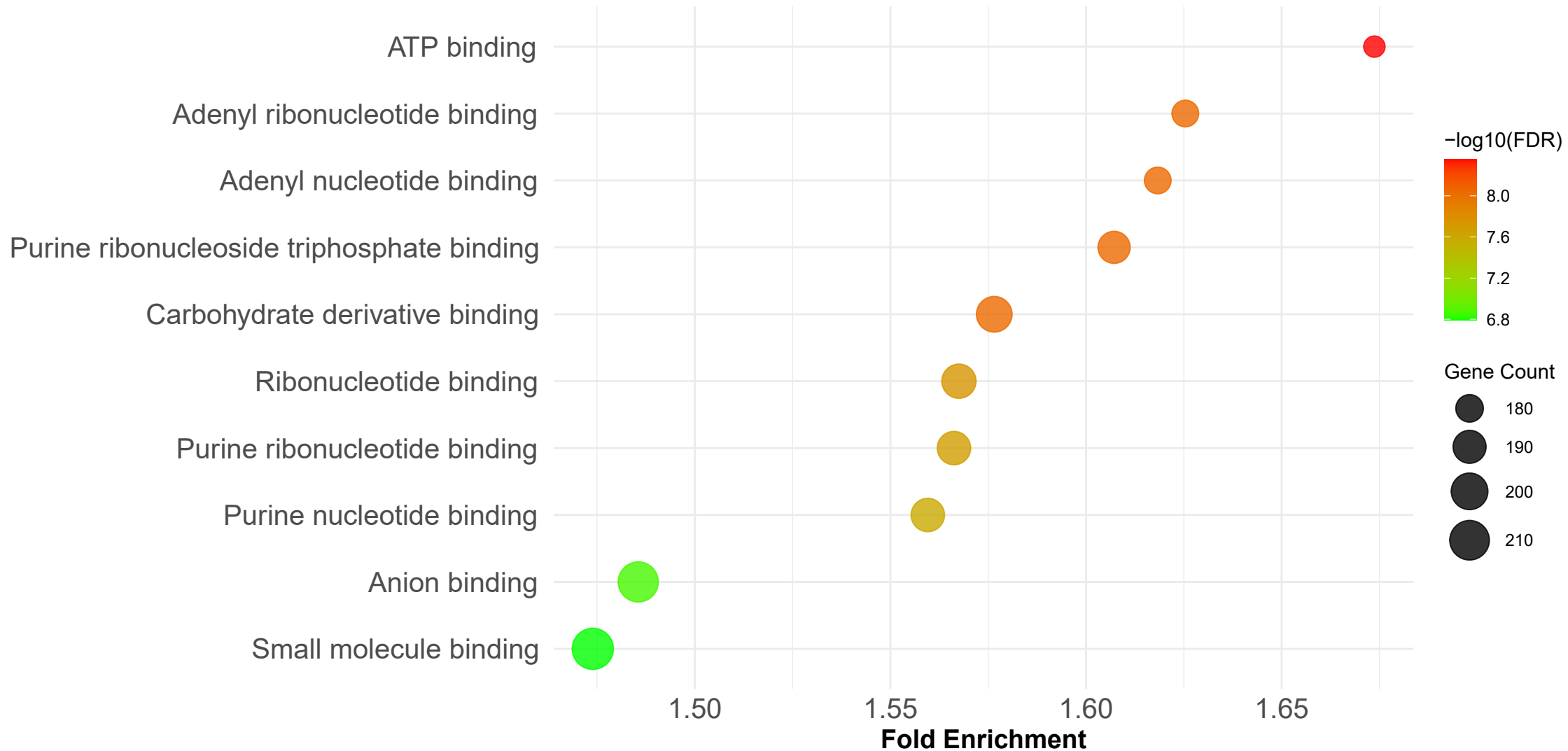

Supplement: Supplementary file 1 [file cimb-48-00173-s001.zip › File S12 Figure/File S10 Figure/Origin Figure/Figure.4-B.pdf]

# GO Cellular Component Enrichment

GO Cellular Component

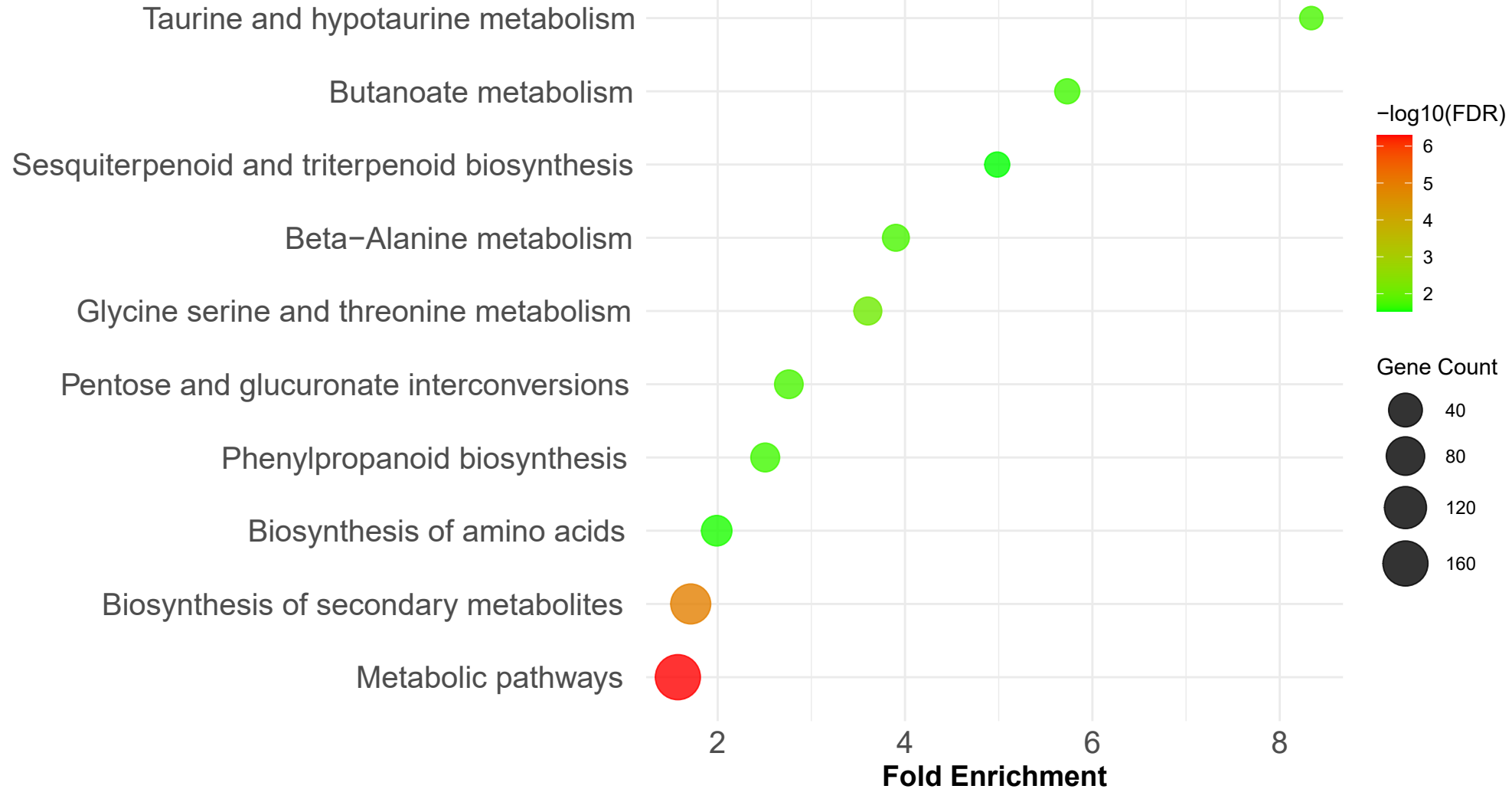

Supplement: Supplementary file 1 [file cimb-48-00173-s001.zip › File S12 Figure/File S10 Figure/Origin Figure/Figure.4-C.pdf]

# KEGG Pathway Enrichment

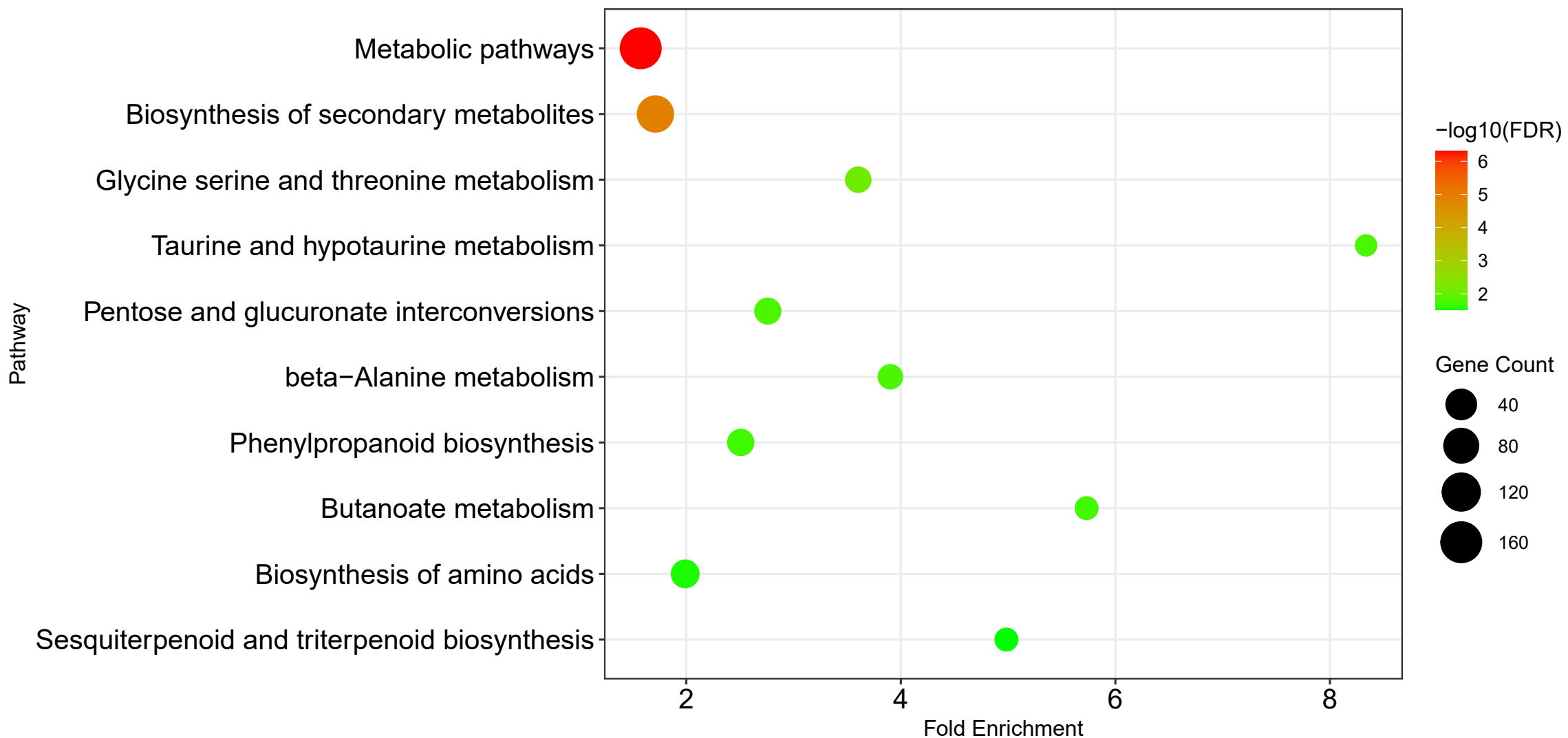

Supplement: Supplementary file 1 [file cimb-48-00173-s001.zip › File S12 Figure/File S10 Figure/Origin Figure/Figure.4-D.pdf]

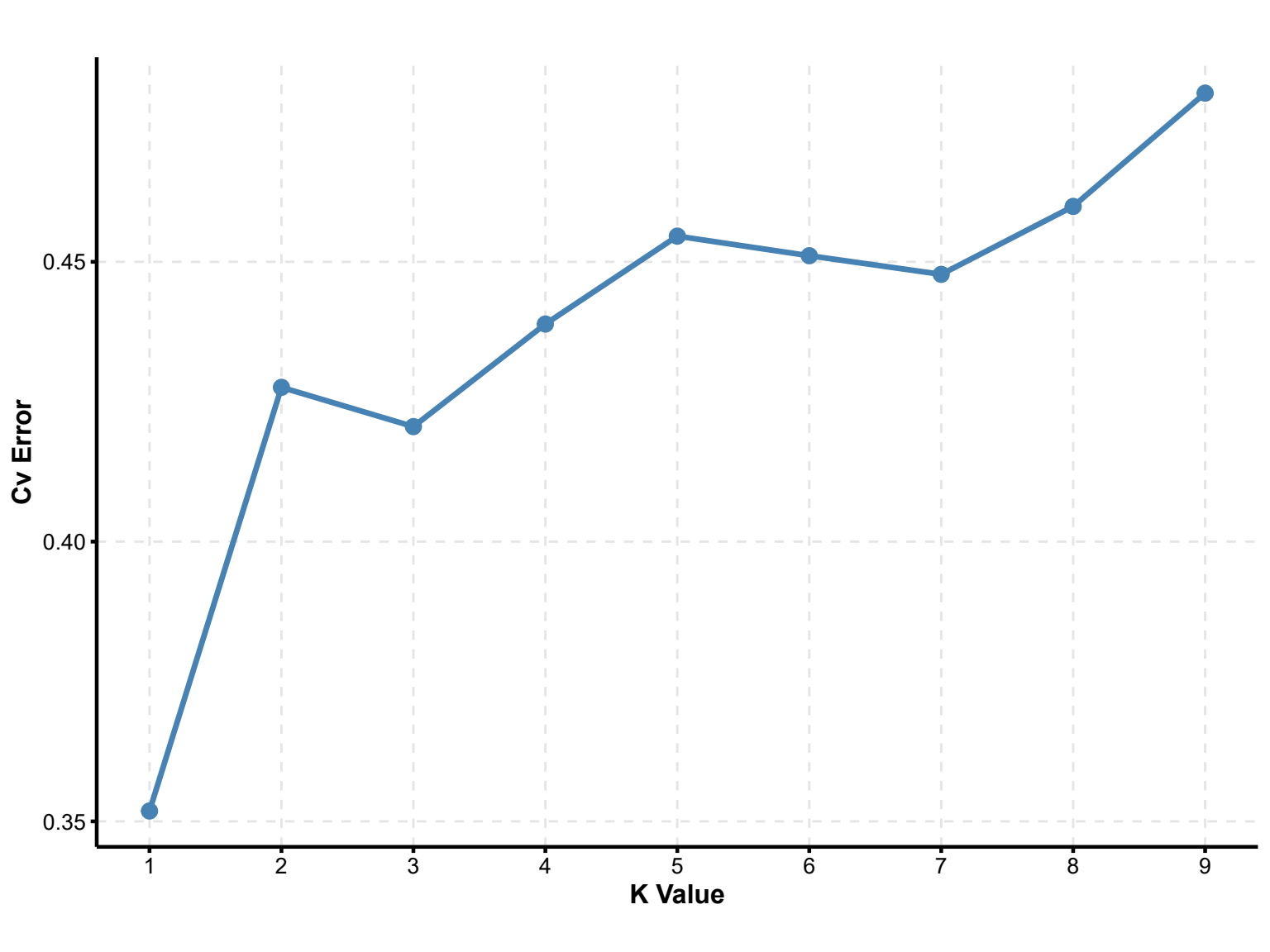

Supplement: Supplementary file 1 [file cimb-48-00173-s001.zip › File S12 Figure/File S10 Figure/Origin Figure/Figure.5-A.pdf]

PCA: PC1 vs PC2

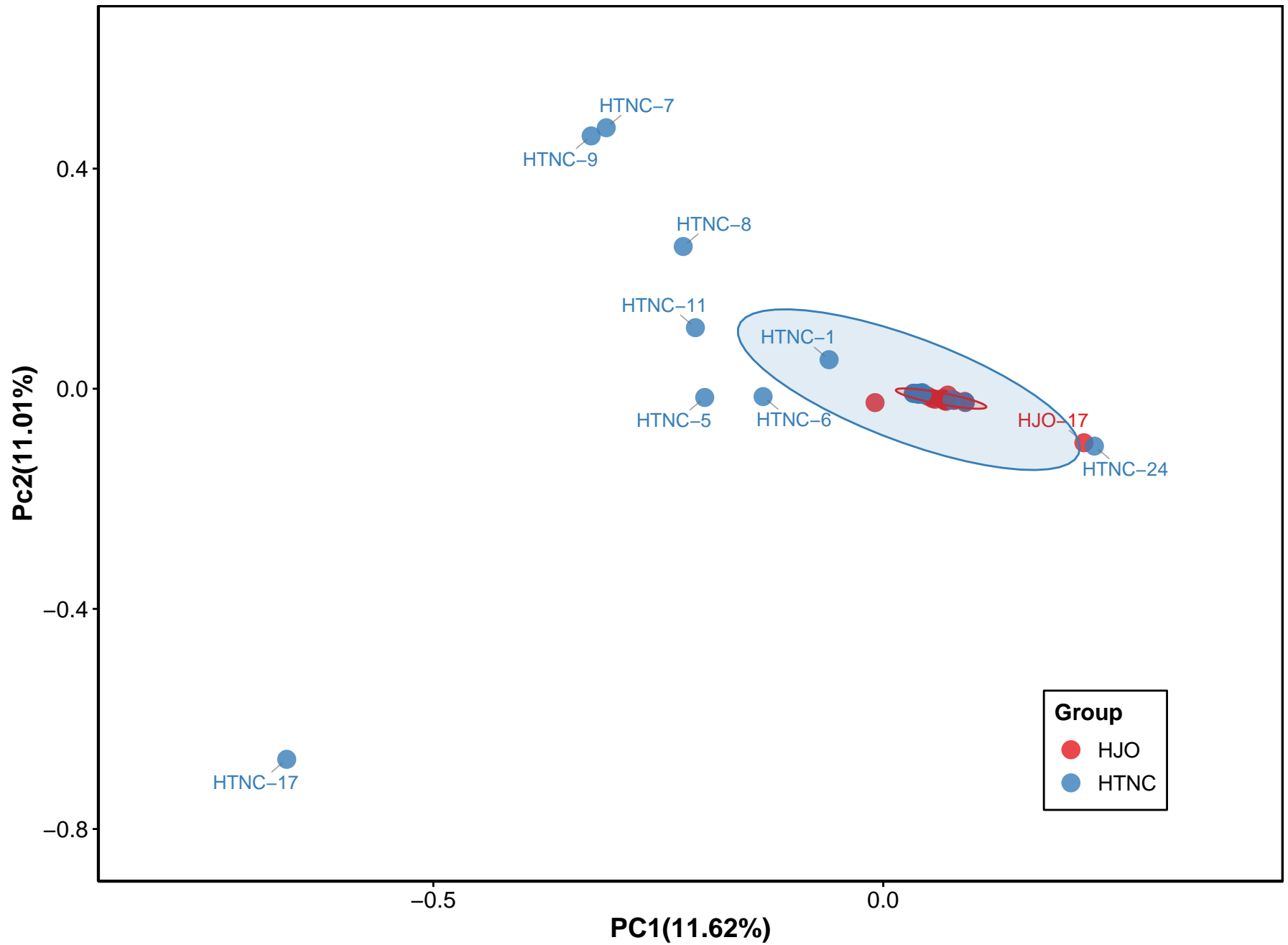

Supplement: Supplementary file 1 [file cimb-48-00173-s001.zip › File S12 Figure/File S10 Figure/Origin Figure/Figure.5-B.pdf]

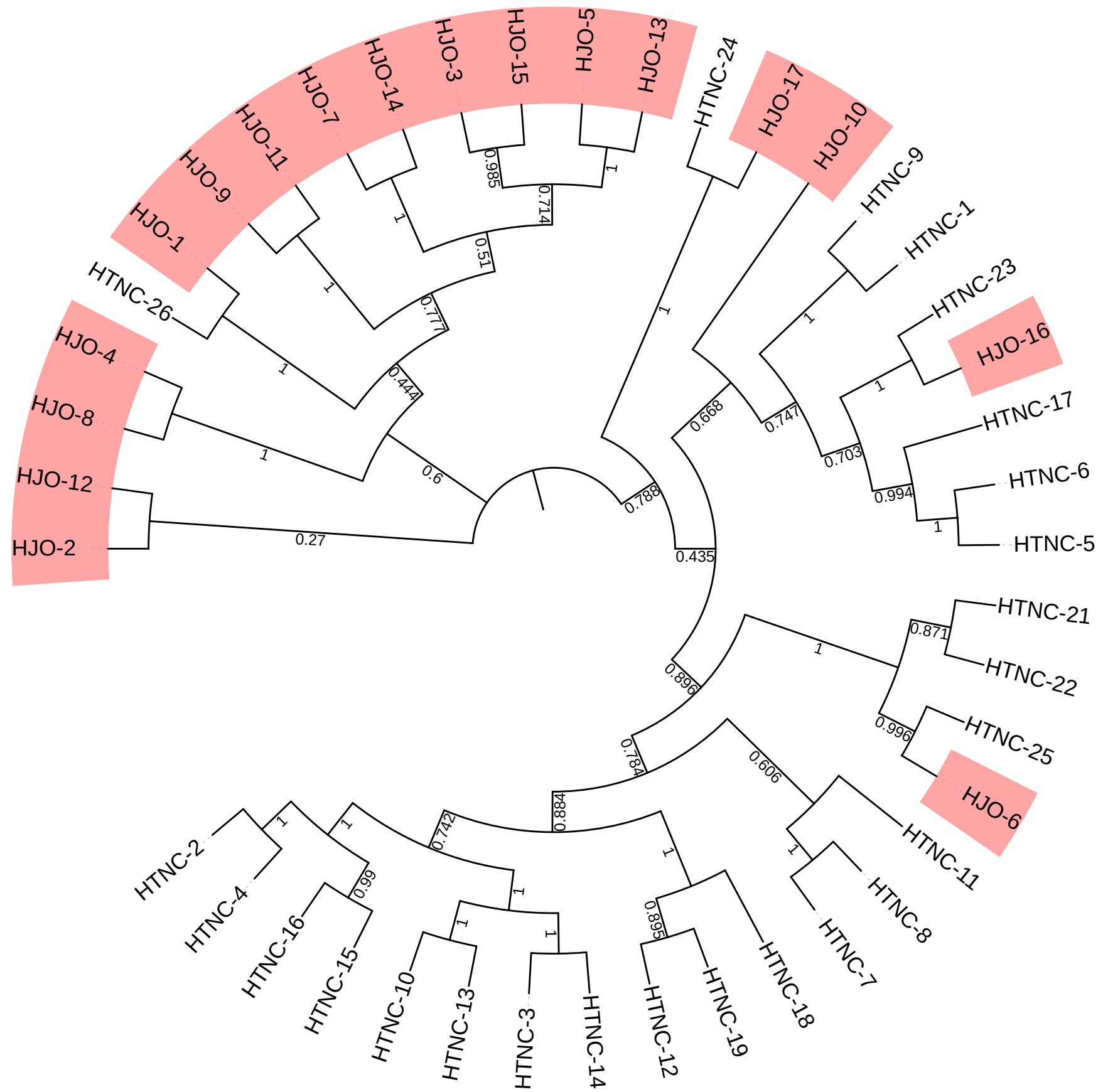

Supplement: Supplementary file 1 [file cimb-48-00173-s001.zip › File S12 Figure/File S10 Figure/Origin Figure/Figure.5-C.pdf]

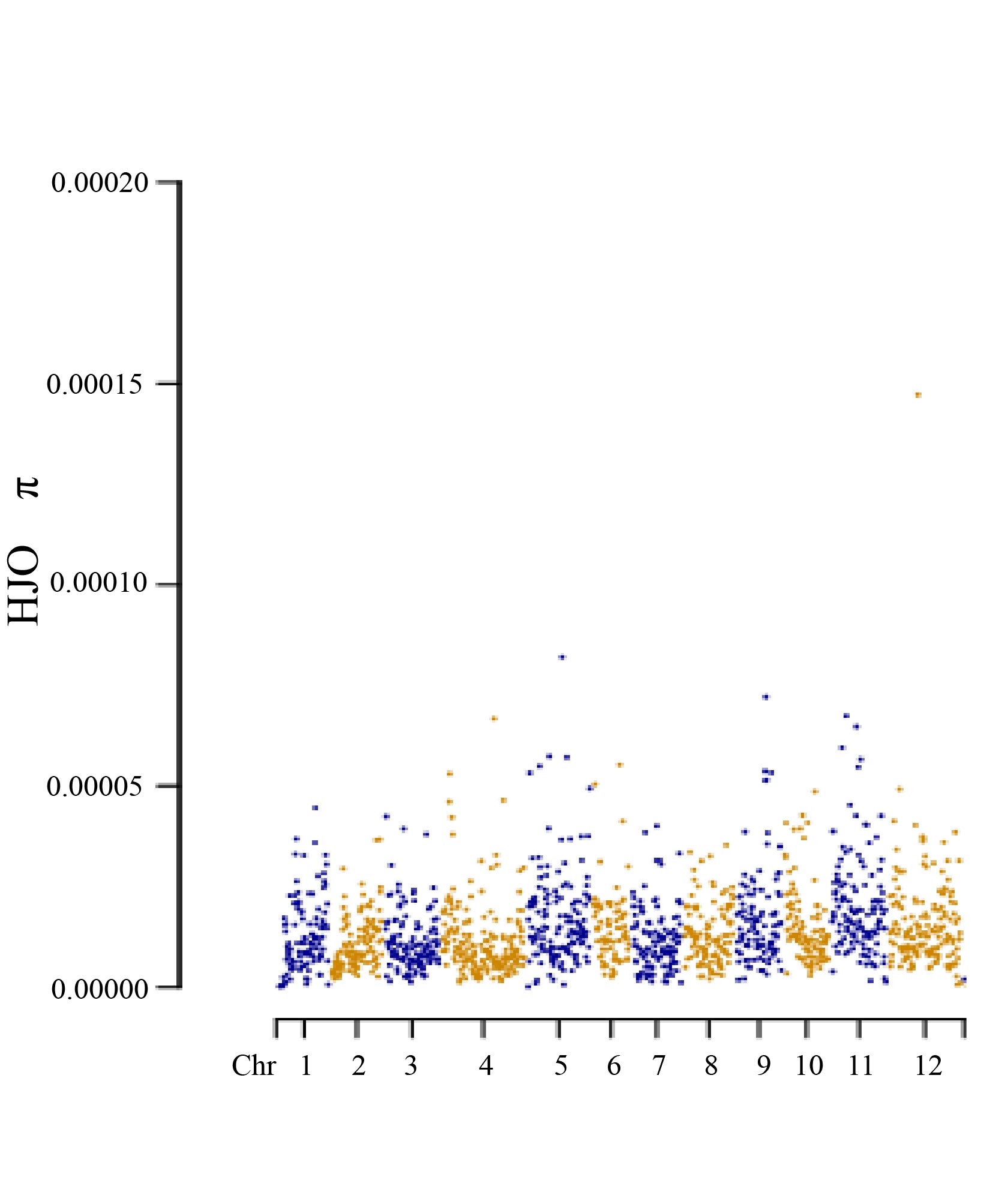

Supplement: Supplementary file 1 [file cimb-48-00173-s001.zip › File S12 Figure/File S10 Figure/Origin Figure/Genetic diversity-a.jpg]

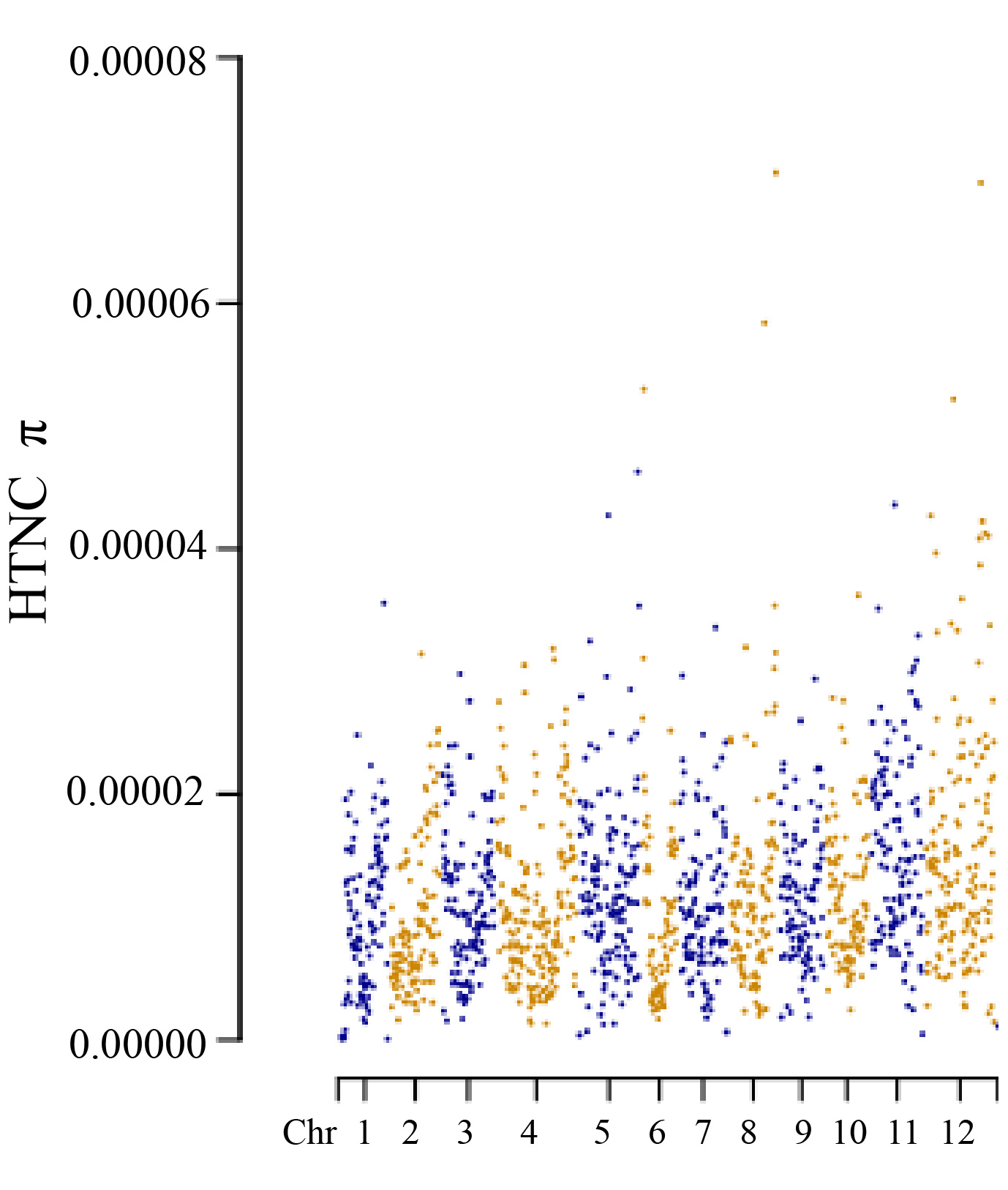

Supplement: Supplementary file 1 [file cimb-48-00173-s001.zip › File S12 Figure/File S10 Figure/Origin Figure/Genetic diversity-b.jpg]

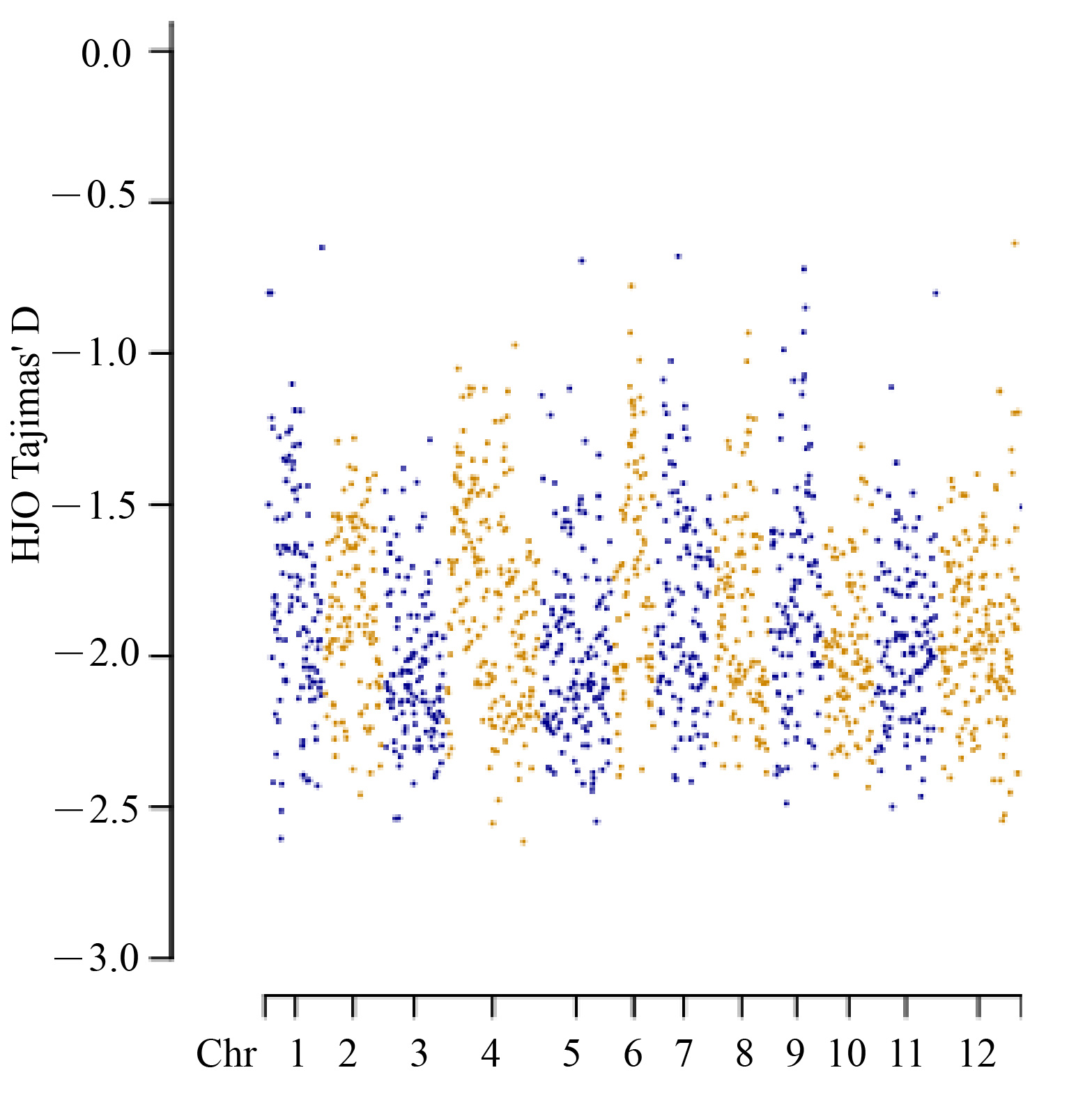

Supplement: Supplementary file 1 [file cimb-48-00173-s001.zip › File S12 Figure/File S10 Figure/Origin Figure/Genetic diversity-c.jpg]

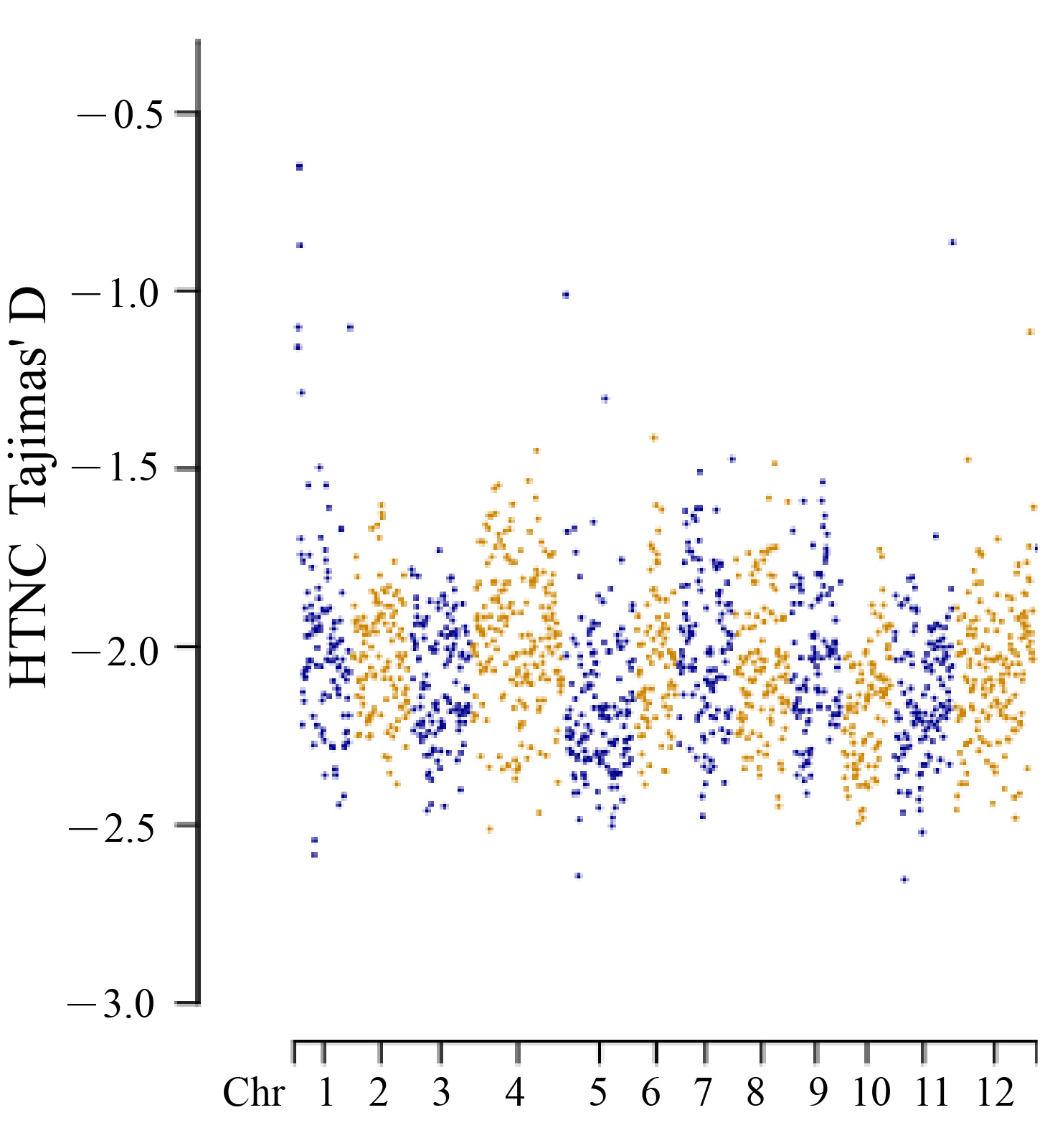

Supplement: Supplementary file 1 [file cimb-48-00173-s001.zip › File S12 Figure/File S10 Figure/Origin Figure/Genetic diversity-d.jpg]

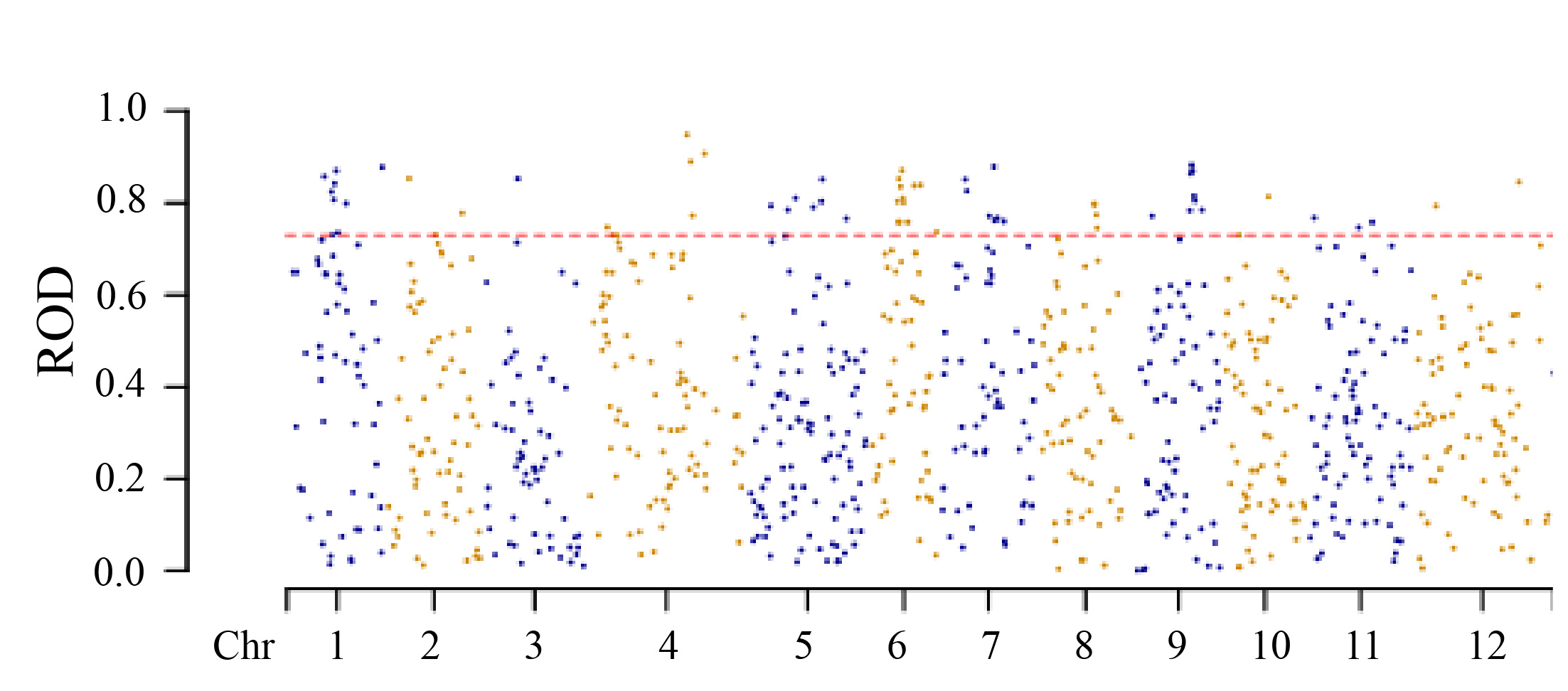

Supplement: Supplementary file 1 [file cimb-48-00173-s001.zip › File S12 Figure/File S10 Figure/Origin Figure/Genetic diversity-e.jpg]

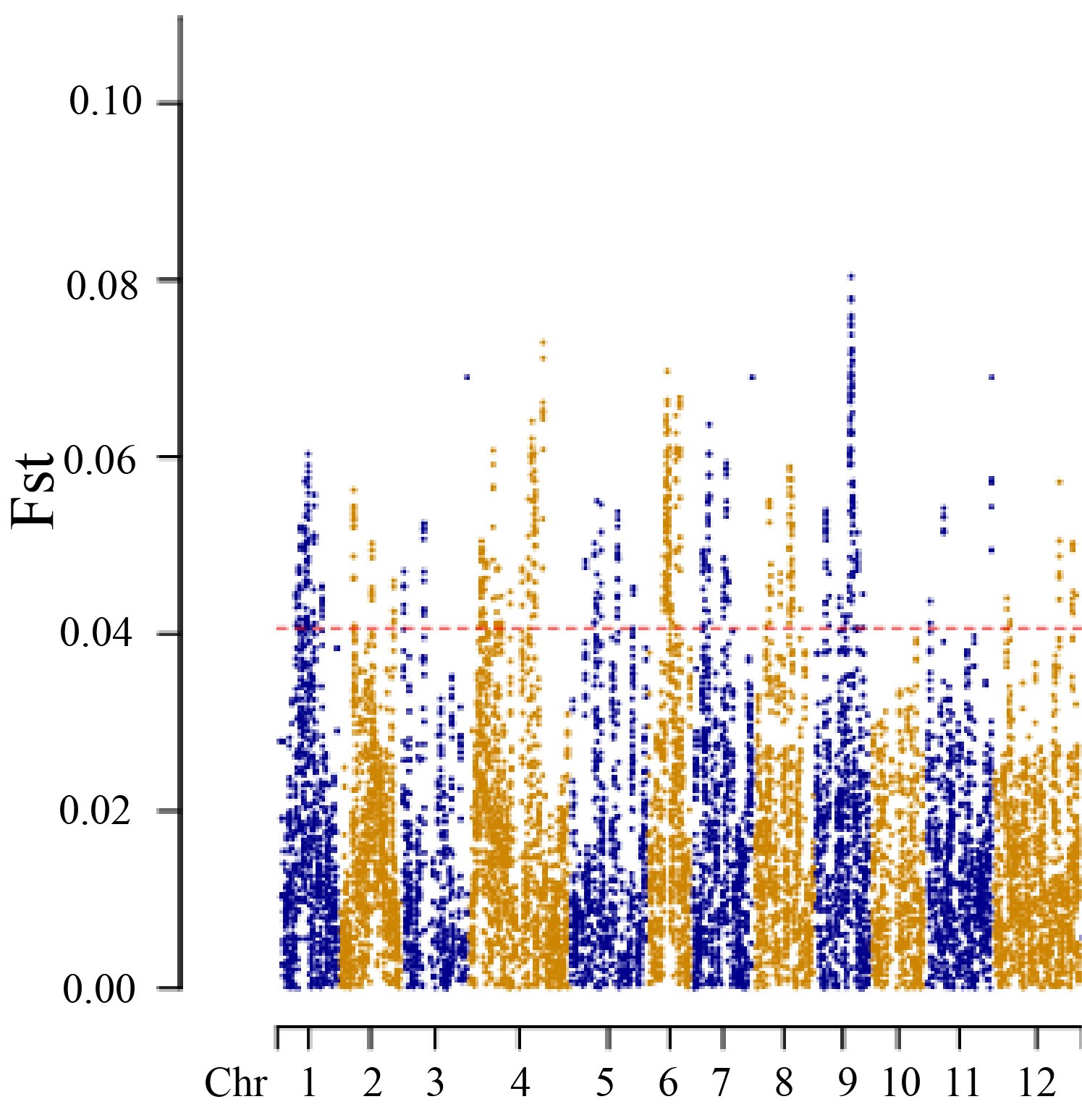

Supplement: Supplementary file 1 [file cimb-48-00173-s001.zip › File S12 Figure/File S10 Figure/Origin Figure/Genetic diversity-f.jpg]

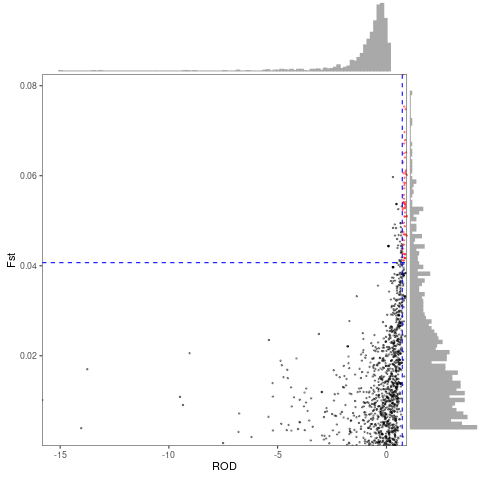

Supplement: Supplementary file 1 [file cimb-48-00173-s001.zip › File S12 Figure/File S10 Figure/Origin Figure/Genetic diversity-g.png]

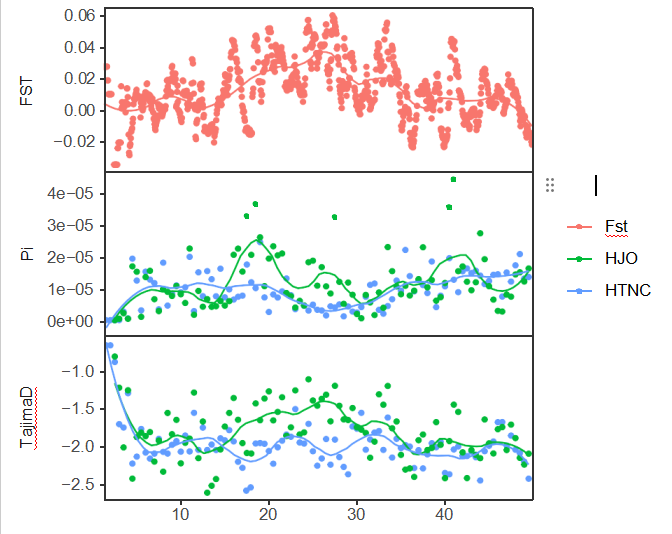

Supplement: Supplementary file 1 [file cimb-48-00173-s001.zip › File S12 Figure/File S10 Figure/Origin Figure/Genetic diversity-h.png]

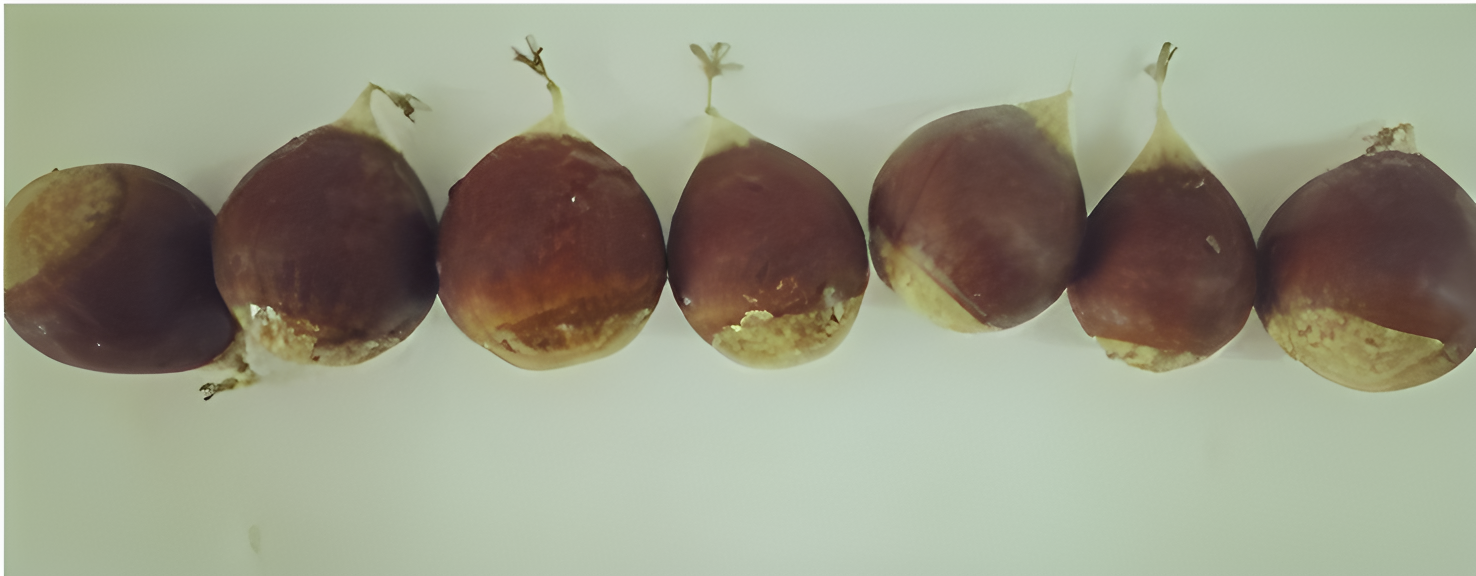

Supplement: Supplementary file 1 [file cimb-48-00173-s001.zip › File S12 Figure/File S10 Figure/Origin Figure/HJO/HJO-1.png]

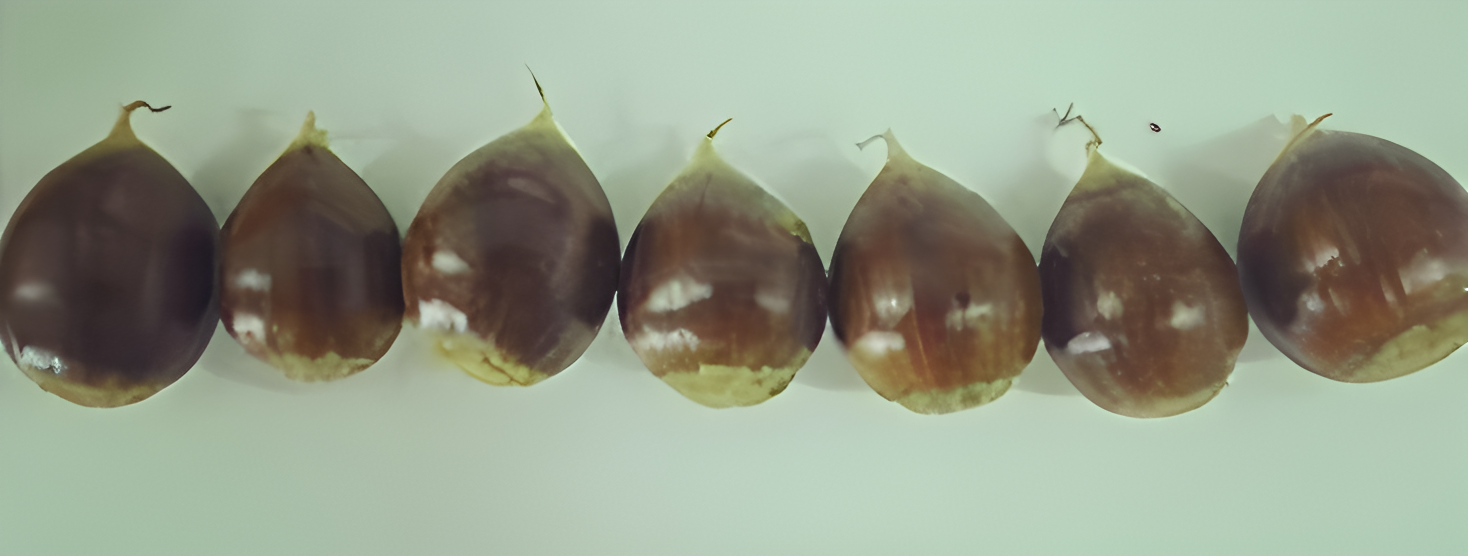

Supplement: Supplementary file 1 [file cimb-48-00173-s001.zip › File S12 Figure/File S10 Figure/Origin Figure/HJO/HJO-10..png]

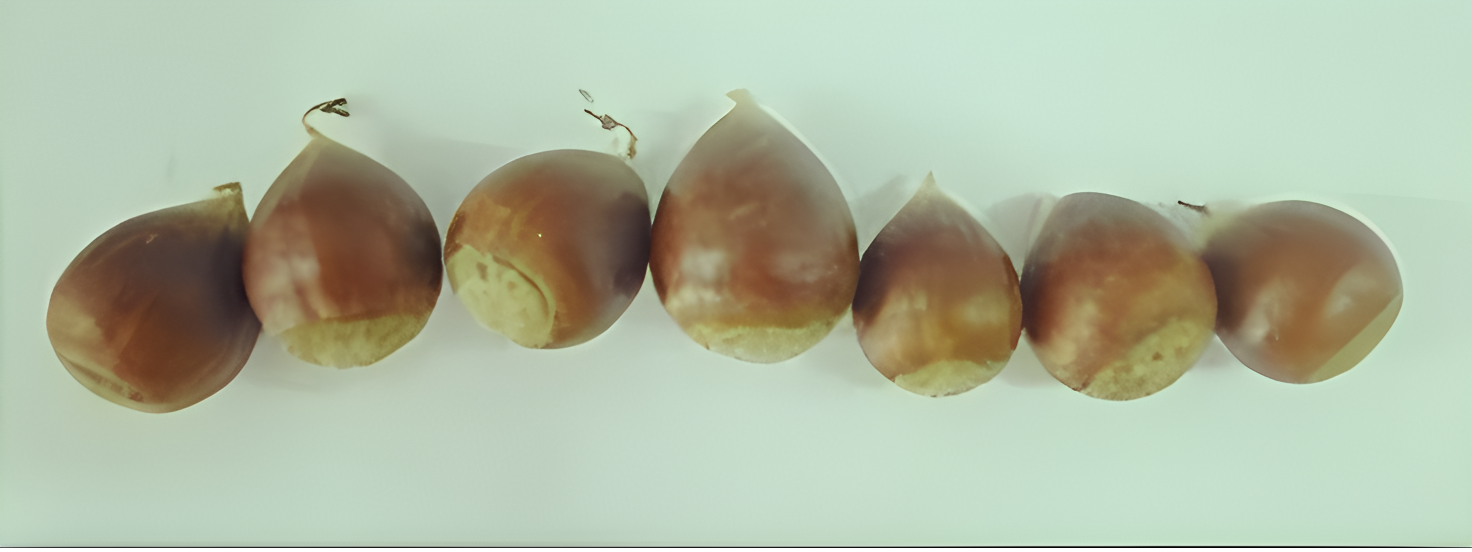

Supplement: Supplementary file 1 [file cimb-48-00173-s001.zip › File S12 Figure/File S10 Figure/Origin Figure/HJO/HJO-11..png]

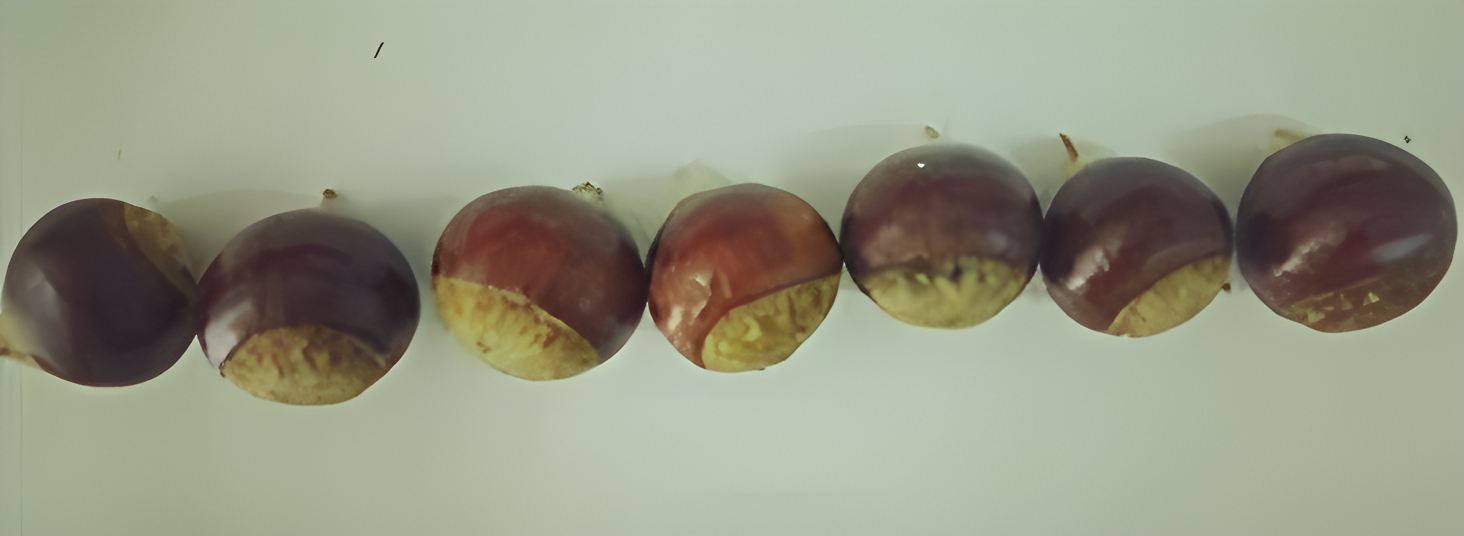

Supplement: Supplementary file 1 [file cimb-48-00173-s001.zip › File S12 Figure/File S10 Figure/Origin Figure/HJO/HJO-12..png]

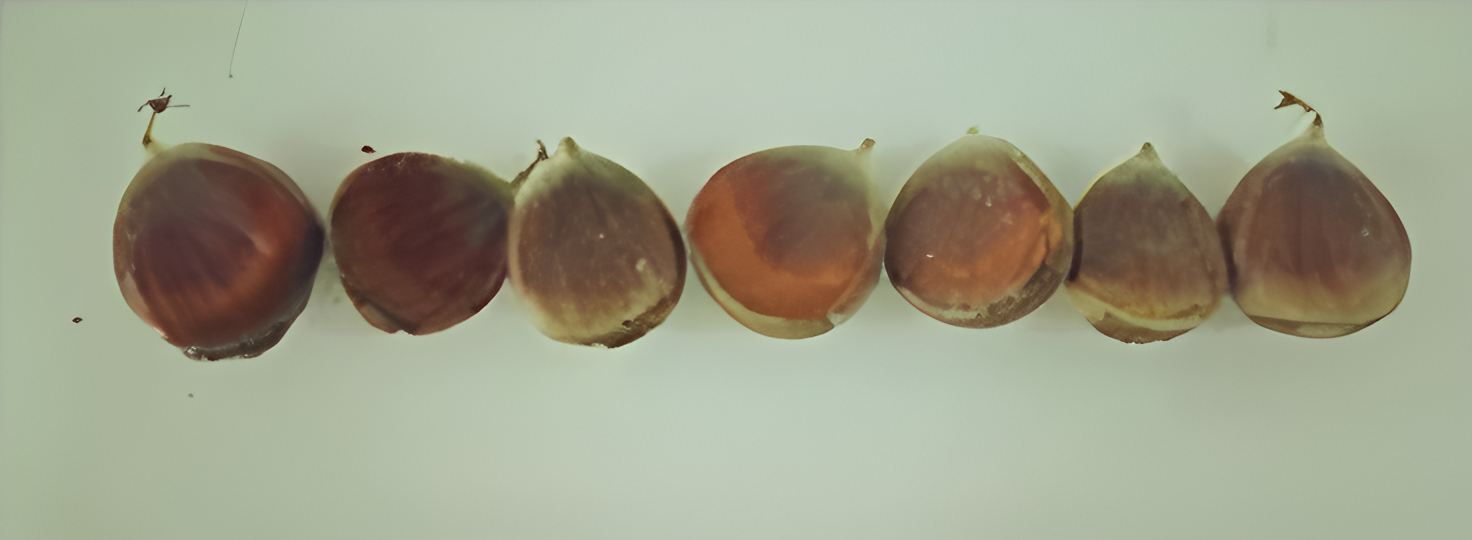

Supplement: Supplementary file 1 [file cimb-48-00173-s001.zip › File S12 Figure/File S10 Figure/Origin Figure/HJO/HJO-13..png]

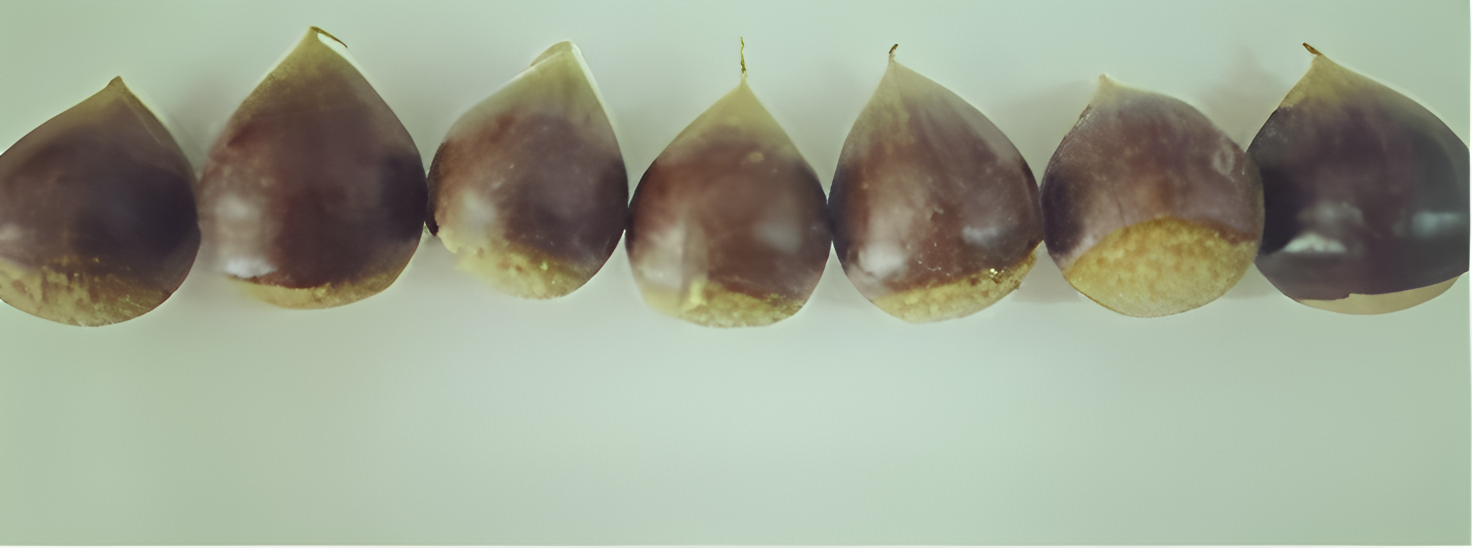

Supplement: Supplementary file 1 [file cimb-48-00173-s001.zip › File S12 Figure/File S10 Figure/Origin Figure/HJO/HJO-14..png]

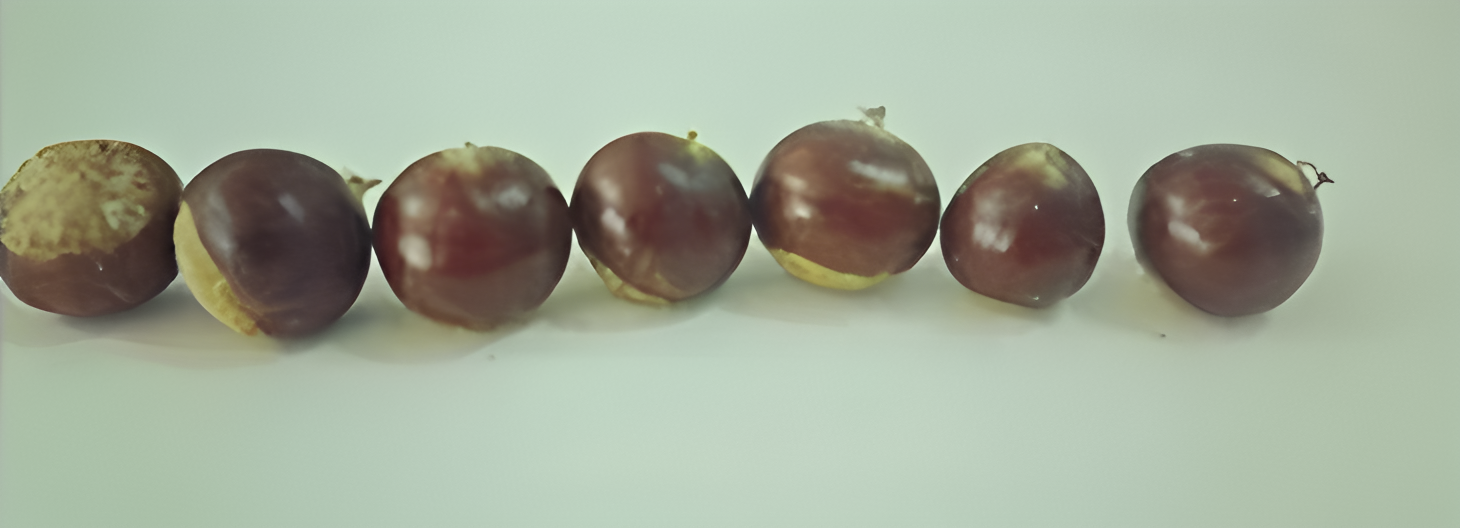

Supplement: Supplementary file 1 [file cimb-48-00173-s001.zip › File S12 Figure/File S10 Figure/Origin Figure/HJO/HJO-15..png]

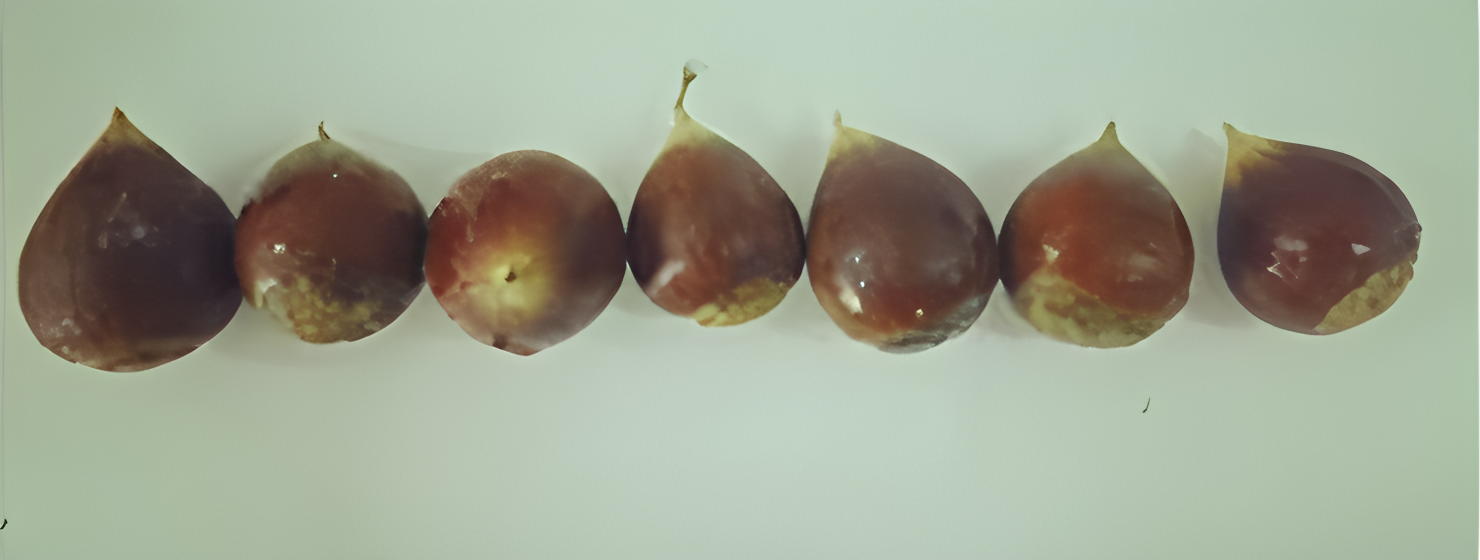

Supplement: Supplementary file 1 [file cimb-48-00173-s001.zip › File S12 Figure/File S10 Figure/Origin Figure/HJO/HJO-16..png]

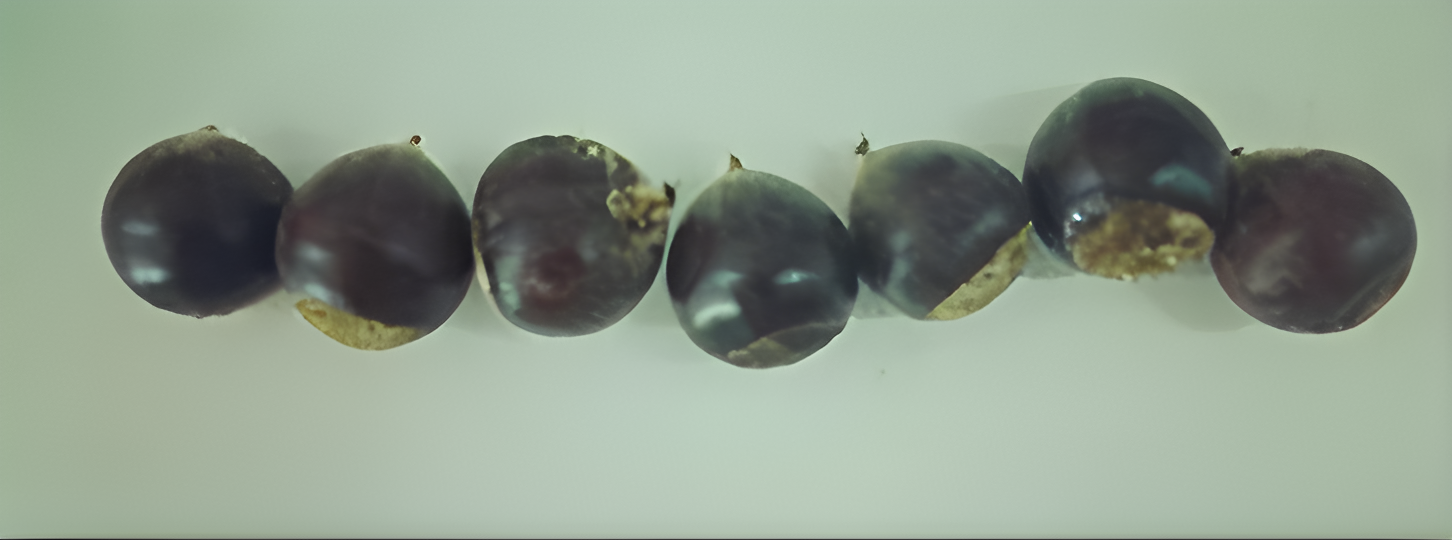

Supplement: Supplementary file 1 [file cimb-48-00173-s001.zip › File S12 Figure/File S10 Figure/Origin Figure/HJO/HJO-17..png]

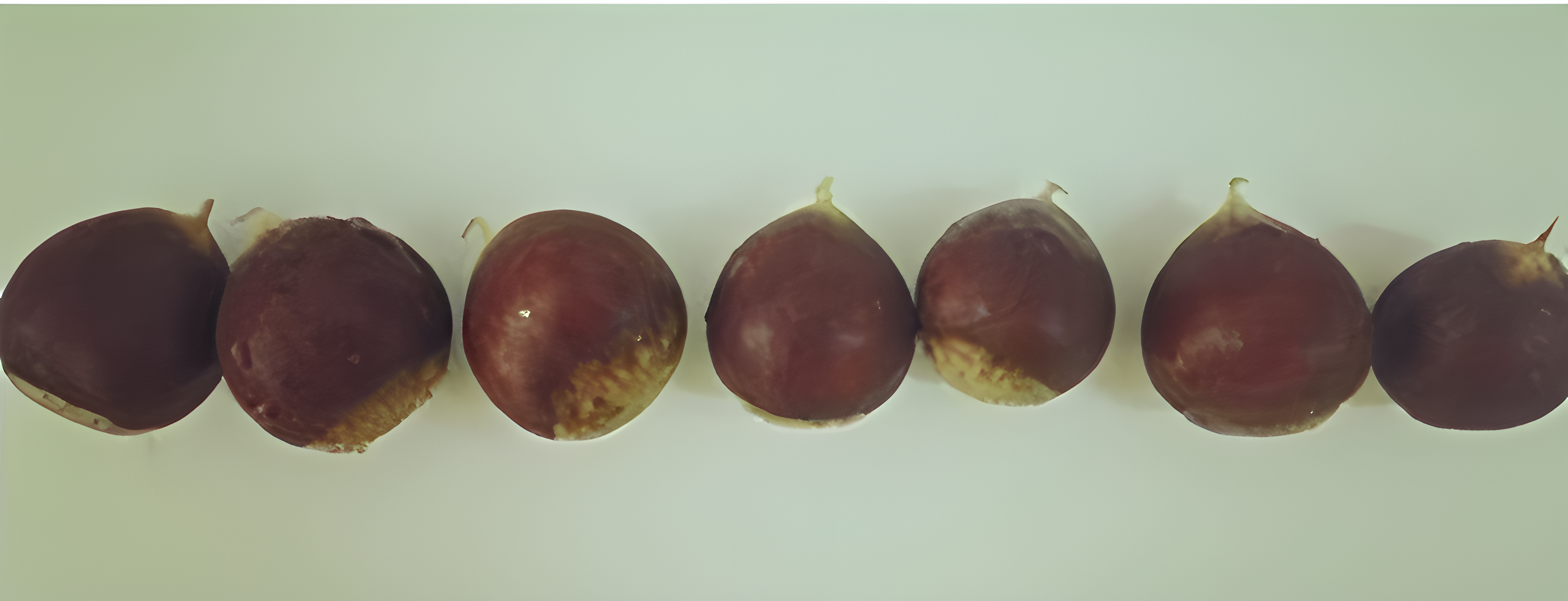

Supplement: Supplementary file 1 [file cimb-48-00173-s001.zip › File S12 Figure/File S10 Figure/Origin Figure/HJO/HJO-2..png]

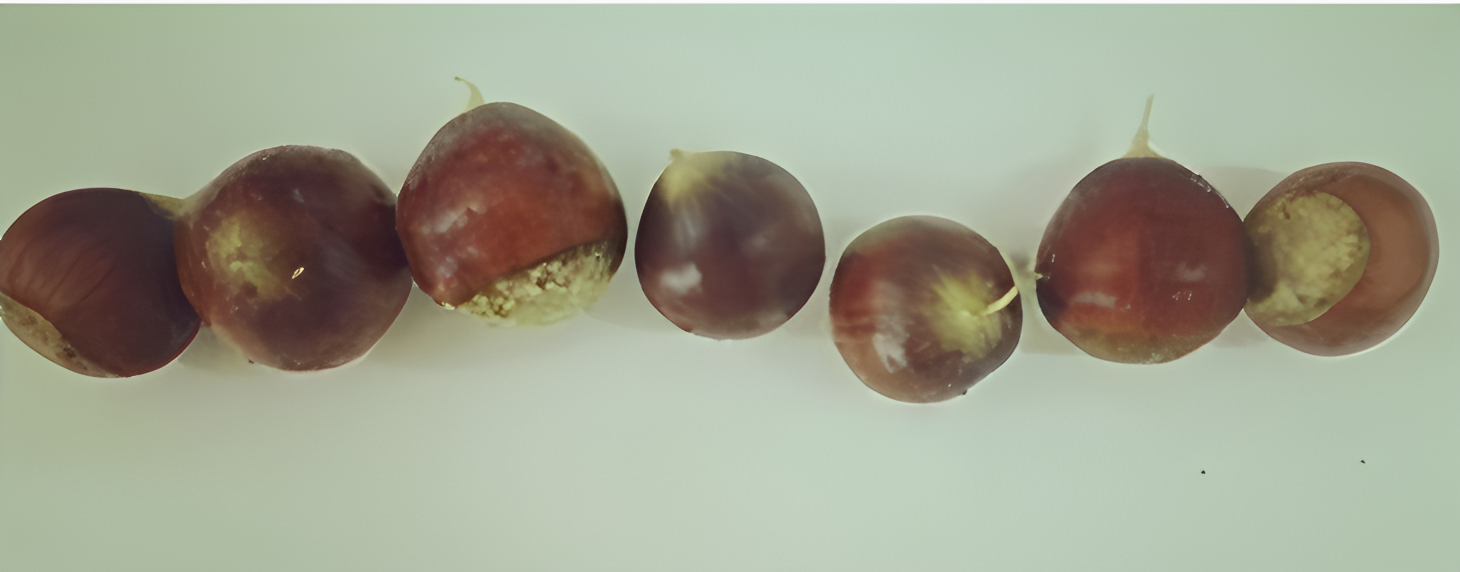

Supplement: Supplementary file 1 [file cimb-48-00173-s001.zip › File S12 Figure/File S10 Figure/Origin Figure/HJO/HJO-3..png]

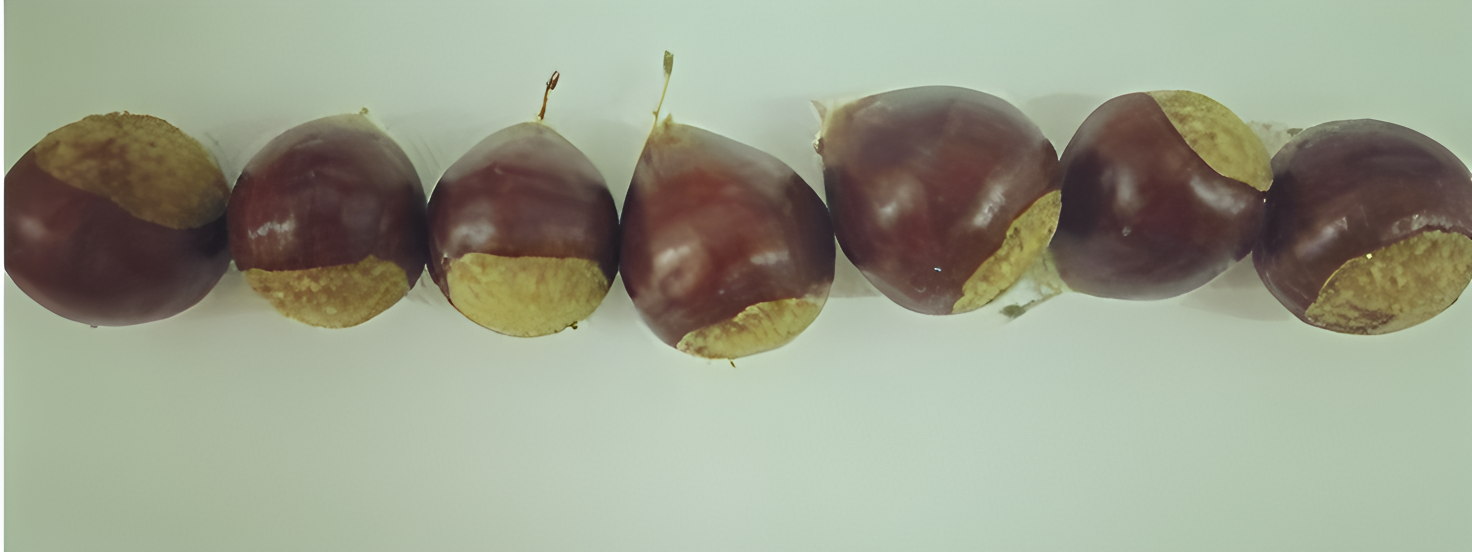

Supplement: Supplementary file 1 [file cimb-48-00173-s001.zip › File S12 Figure/File S10 Figure/Origin Figure/HJO/HJO-4..png]

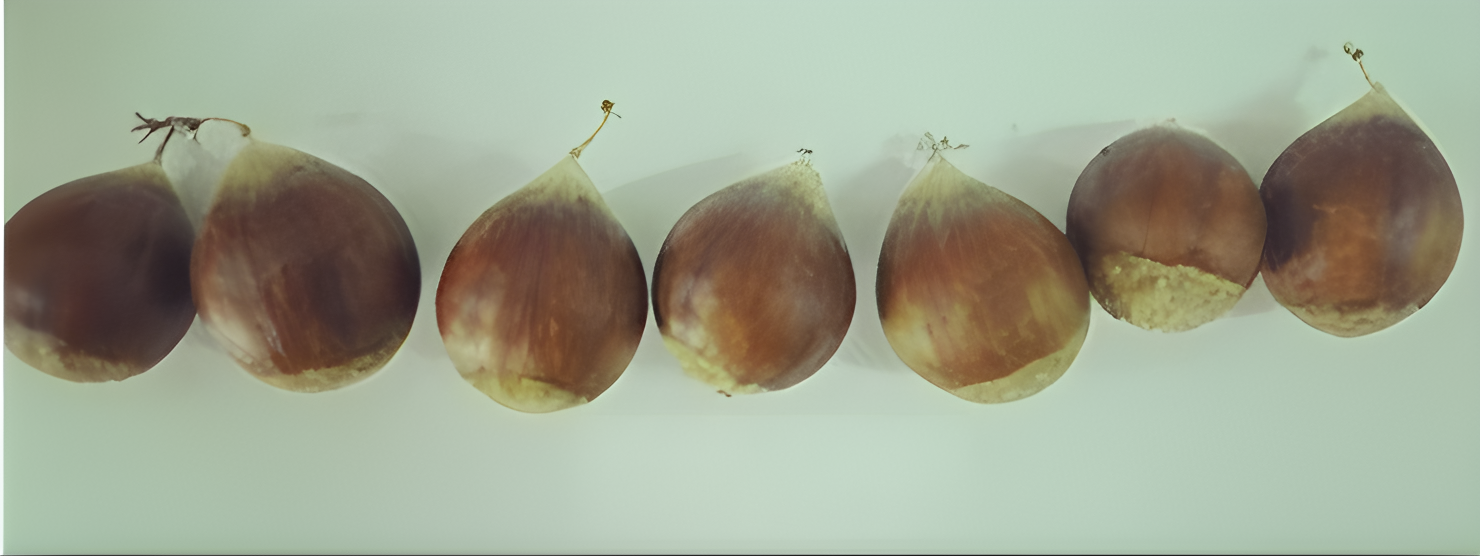

Supplement: Supplementary file 1 [file cimb-48-00173-s001.zip › File S12 Figure/File S10 Figure/Origin Figure/HJO/HJO-5..png]

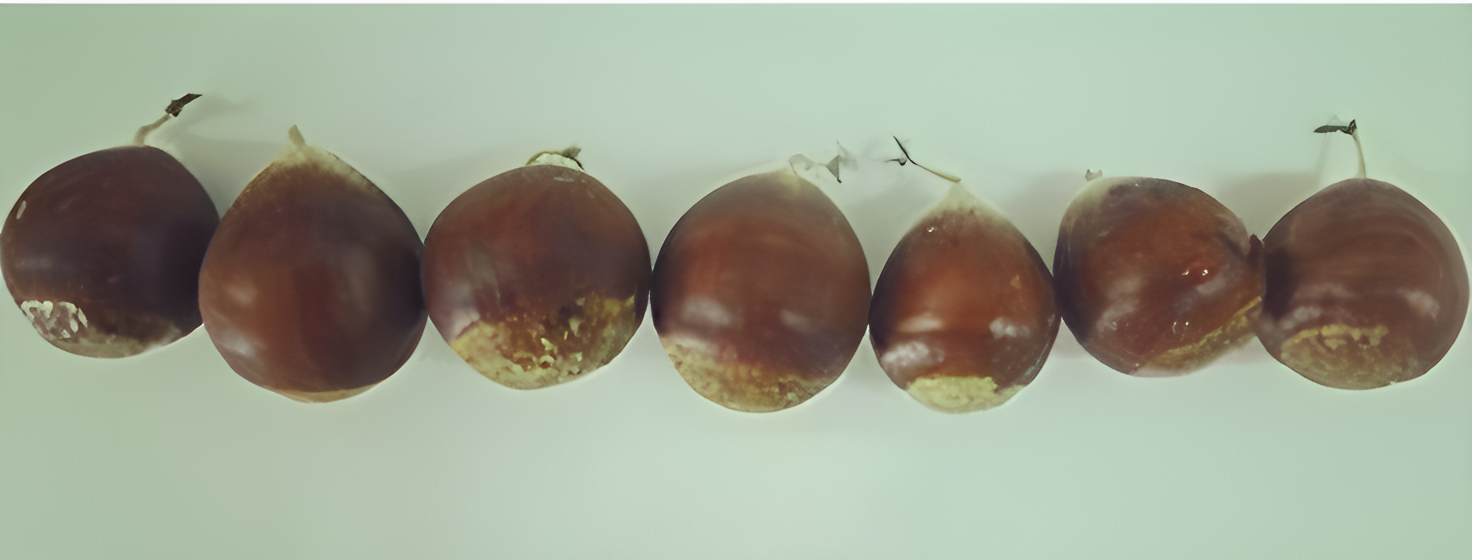

Supplement: Supplementary file 1 [file cimb-48-00173-s001.zip › File S12 Figure/File S10 Figure/Origin Figure/HJO/HJO-6..png]

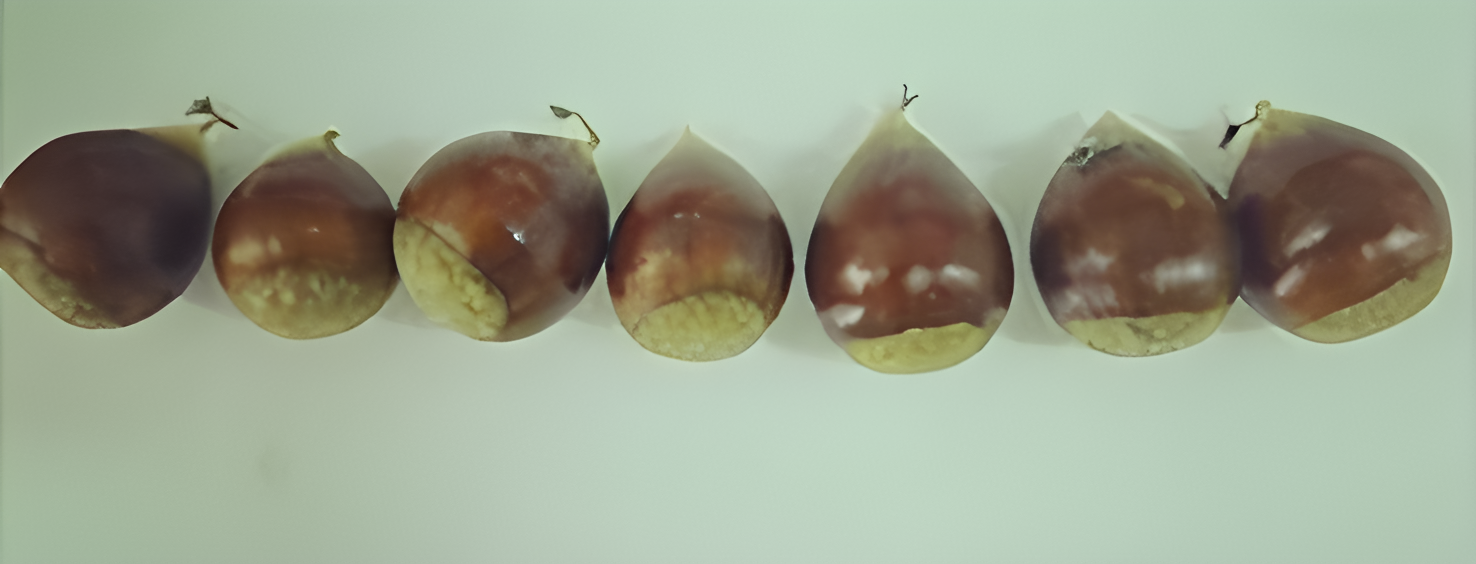

Supplement: Supplementary file 1 [file cimb-48-00173-s001.zip › File S12 Figure/File S10 Figure/Origin Figure/HJO/HJO-7..png]

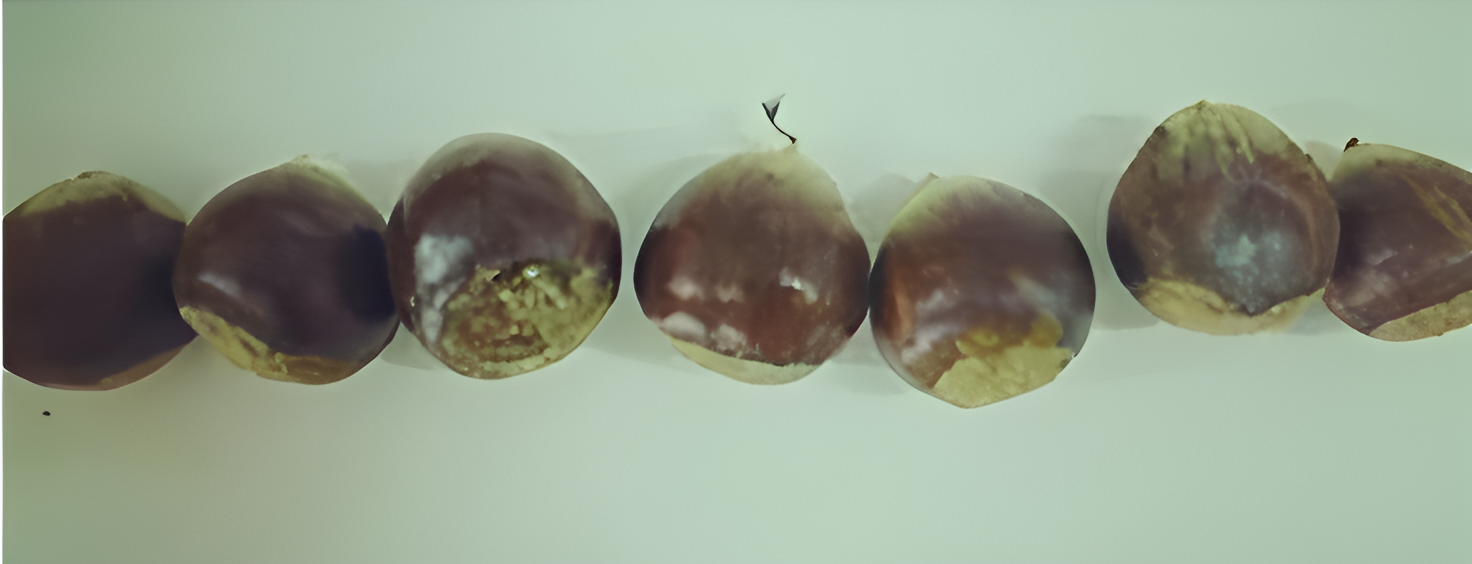

Supplement: Supplementary file 1 [file cimb-48-00173-s001.zip › File S12 Figure/File S10 Figure/Origin Figure/HJO/HJO-8..png]

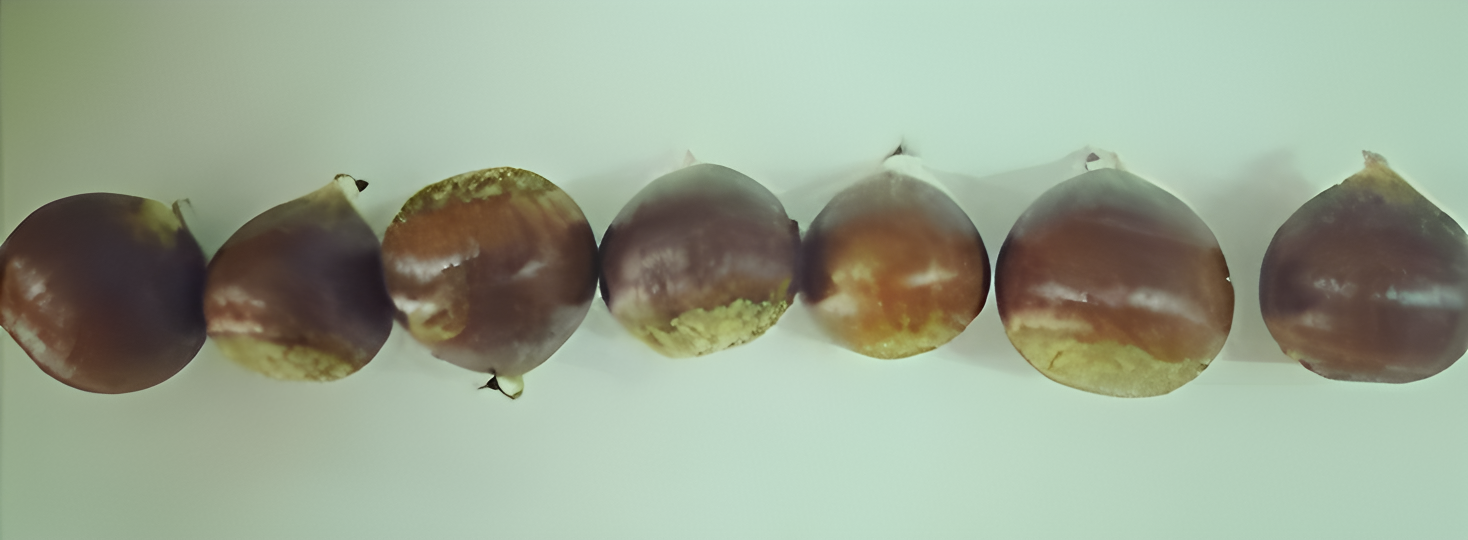

Supplement: Supplementary file 1 [file cimb-48-00173-s001.zip › File S12 Figure/File S10 Figure/Origin Figure/HJO/HJO-9..png]

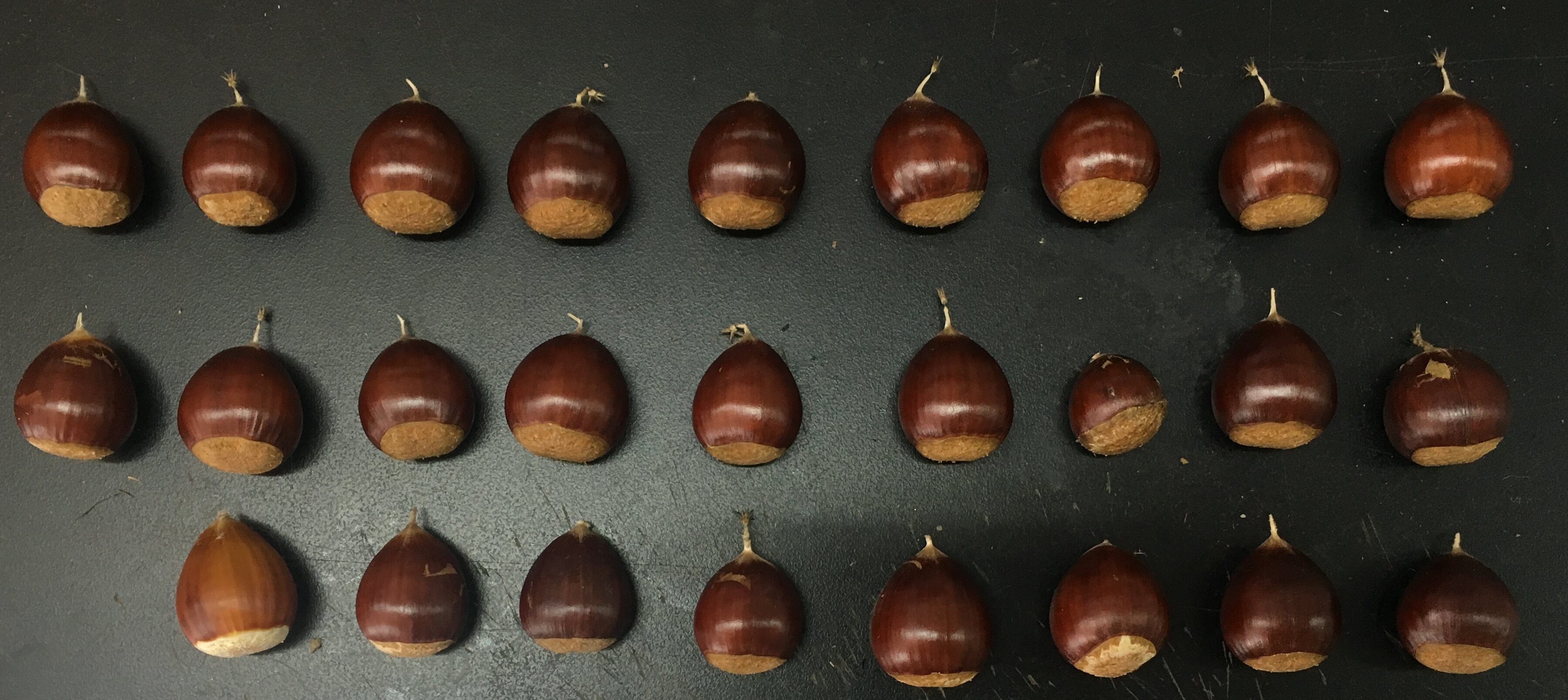

Supplement: Supplementary file 1 [file cimb-48-00173-s001.zip › File S12 Figure/File S10 Figure/Origin Figure/HTNC/HCTN-1.JPG]

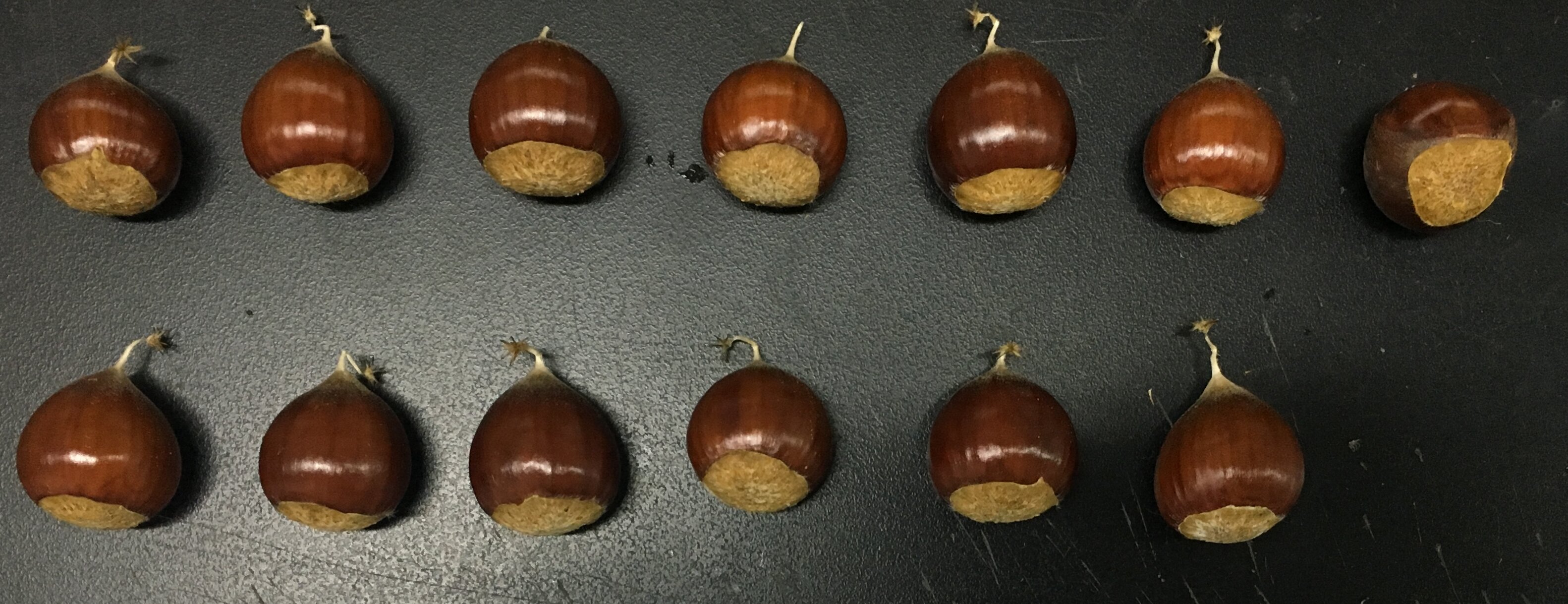

Supplement: Supplementary file 1 [file cimb-48-00173-s001.zip › File S12 Figure/File S10 Figure/Origin Figure/HTNC/HCTN-10.JPG]

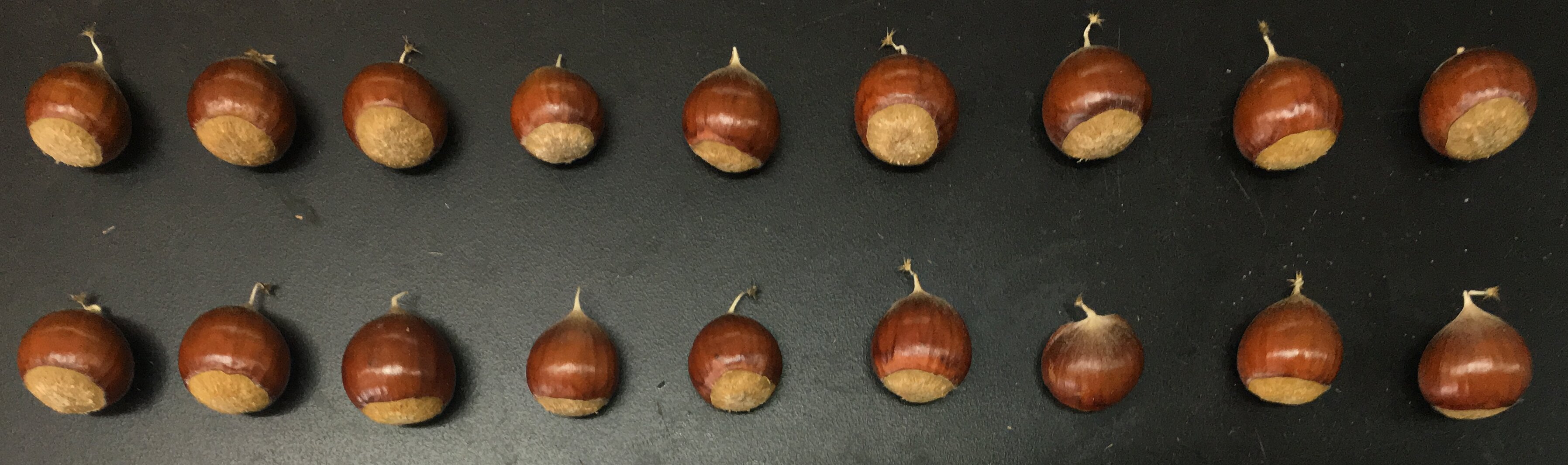

Supplement: Supplementary file 1 [file cimb-48-00173-s001.zip › File S12 Figure/File S10 Figure/Origin Figure/HTNC/HCTN-11.JPG]

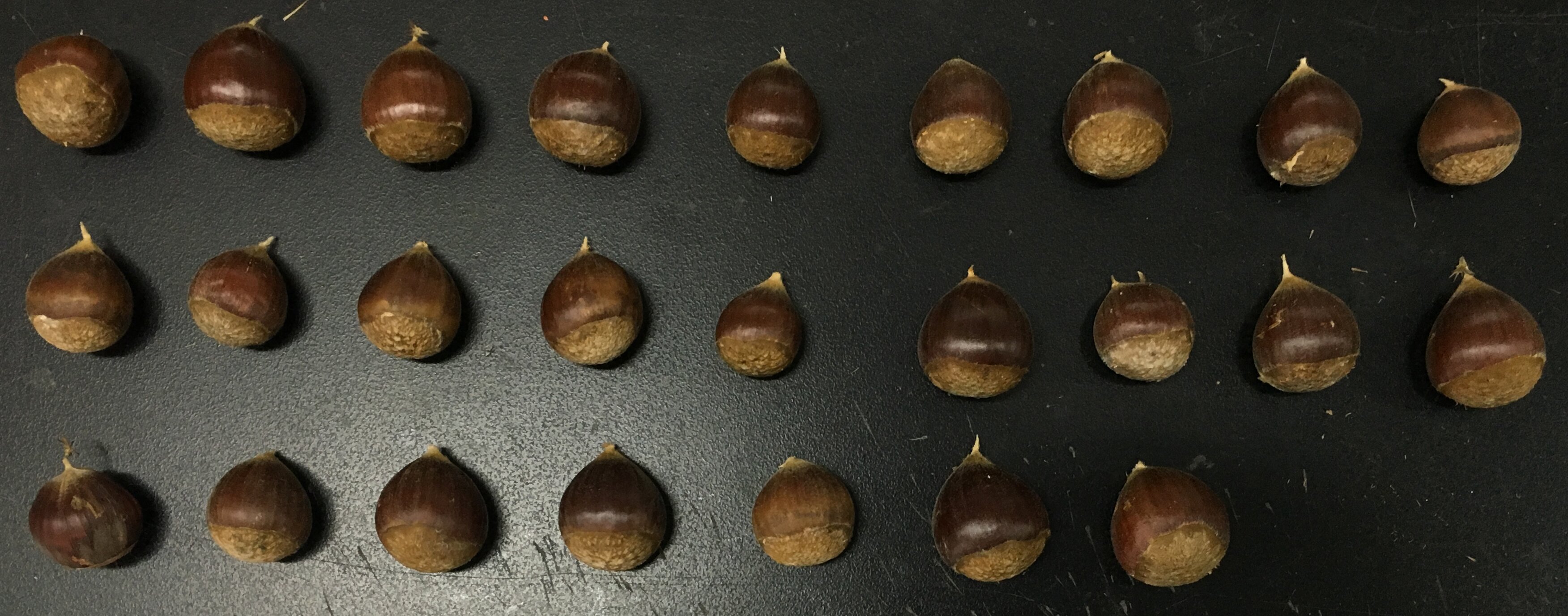

Supplement: Supplementary file 1 [file cimb-48-00173-s001.zip › File S12 Figure/File S10 Figure/Origin Figure/HTNC/HCTN-12.JPG]

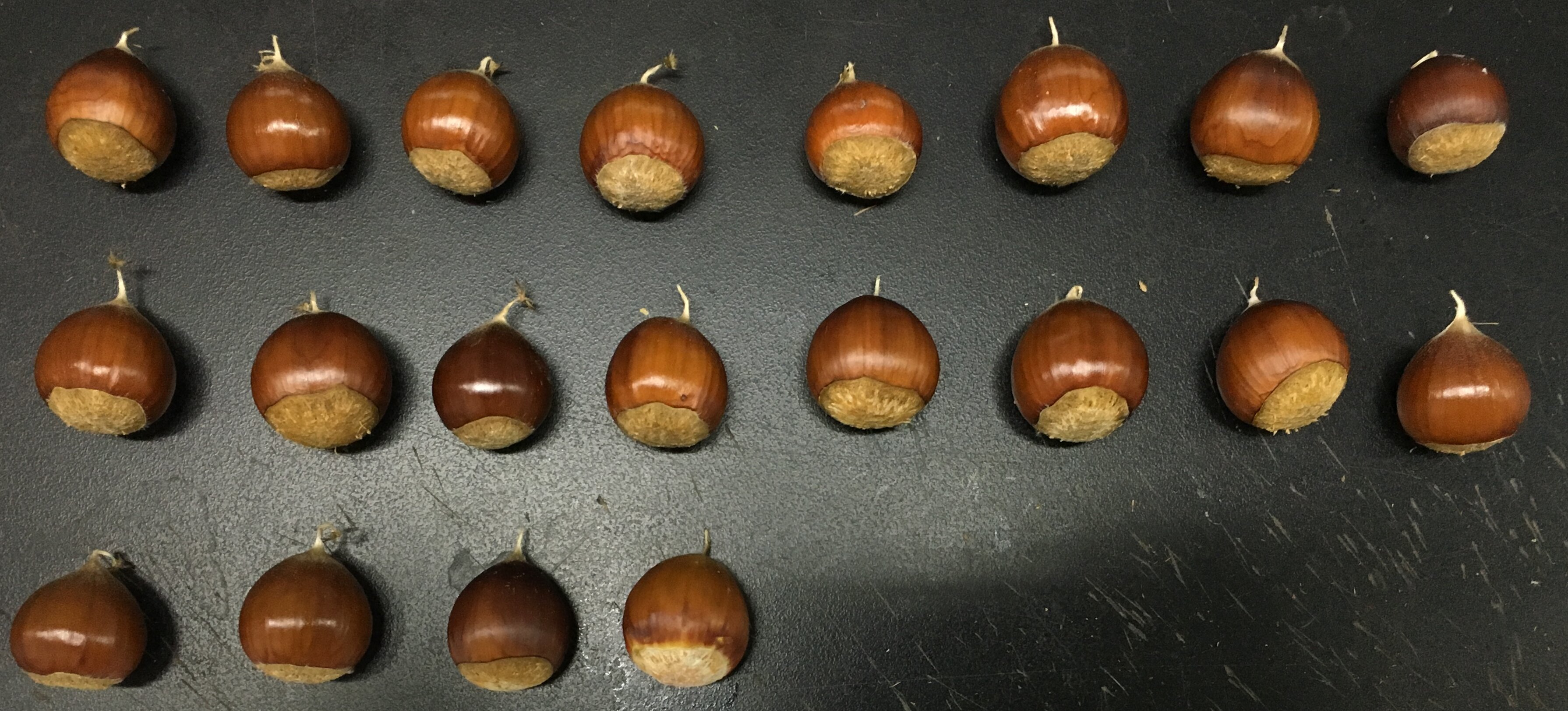

Supplement: Supplementary file 1 [file cimb-48-00173-s001.zip › File S12 Figure/File S10 Figure/Origin Figure/HTNC/HCTN-13.JPG]

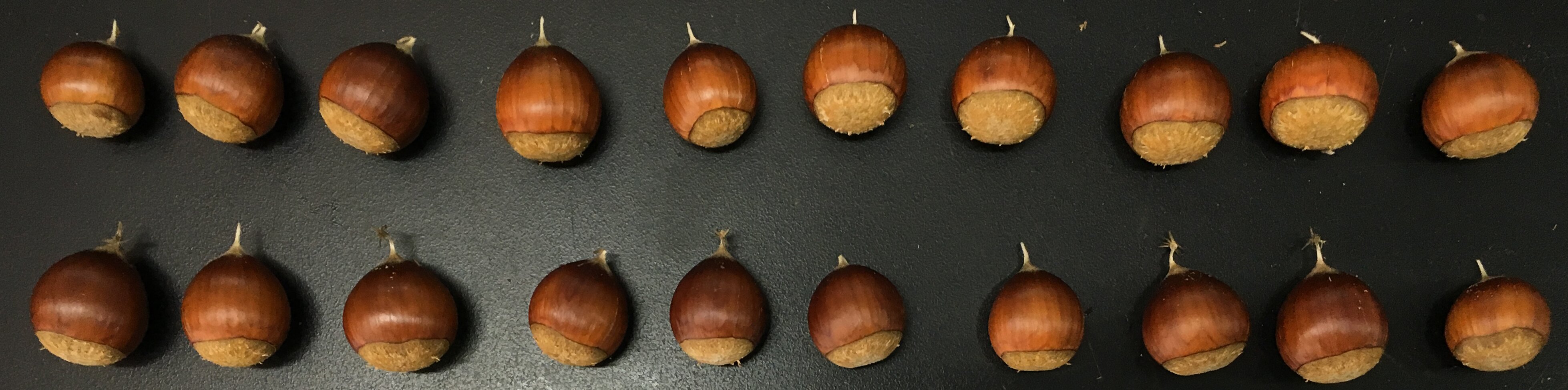

Supplement: Supplementary file 1 [file cimb-48-00173-s001.zip › File S12 Figure/File S10 Figure/Origin Figure/HTNC/HCTN-14.JPG]

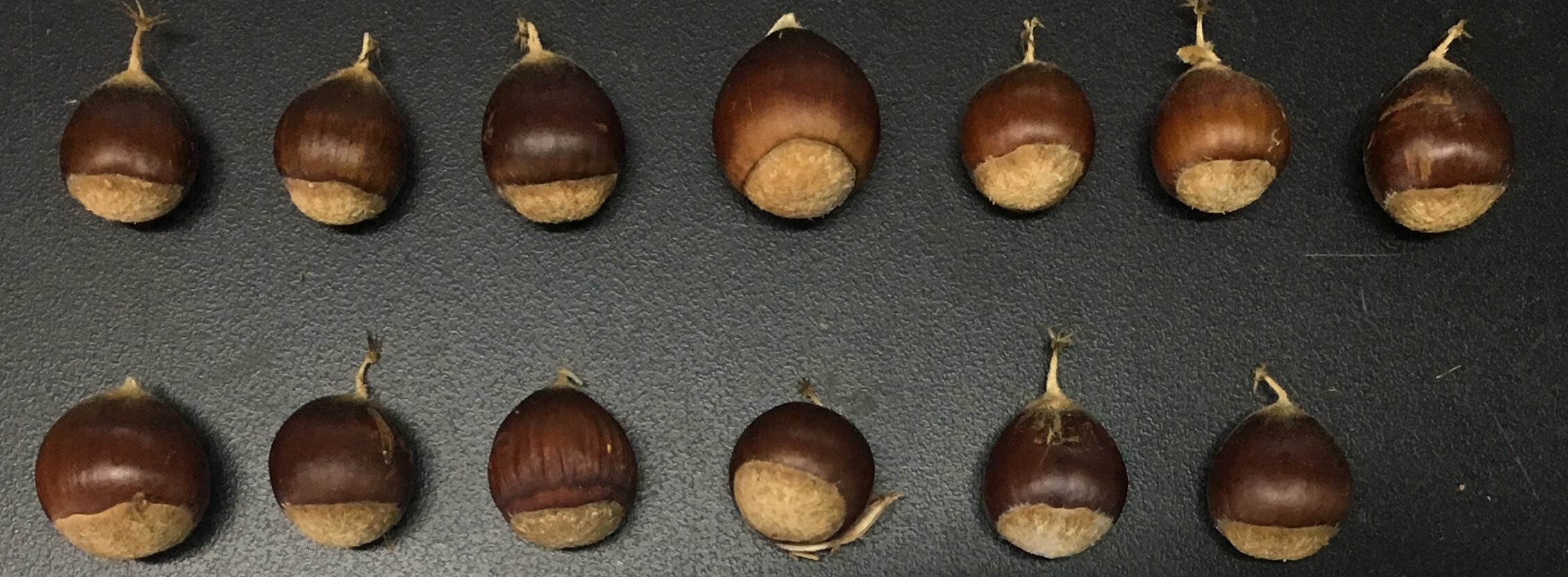

Supplement: Supplementary file 1 [file cimb-48-00173-s001.zip › File S12 Figure/File S10 Figure/Origin Figure/HTNC/HCTN-15.JPG]

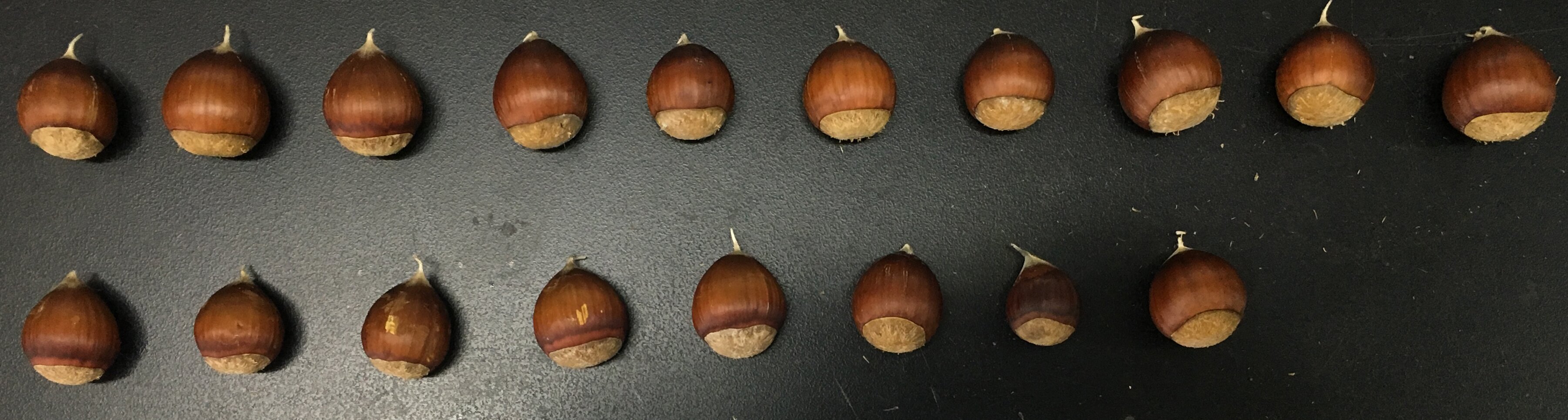

Supplement: Supplementary file 1 [file cimb-48-00173-s001.zip › File S12 Figure/File S10 Figure/Origin Figure/HTNC/HCTN-16.JPG]

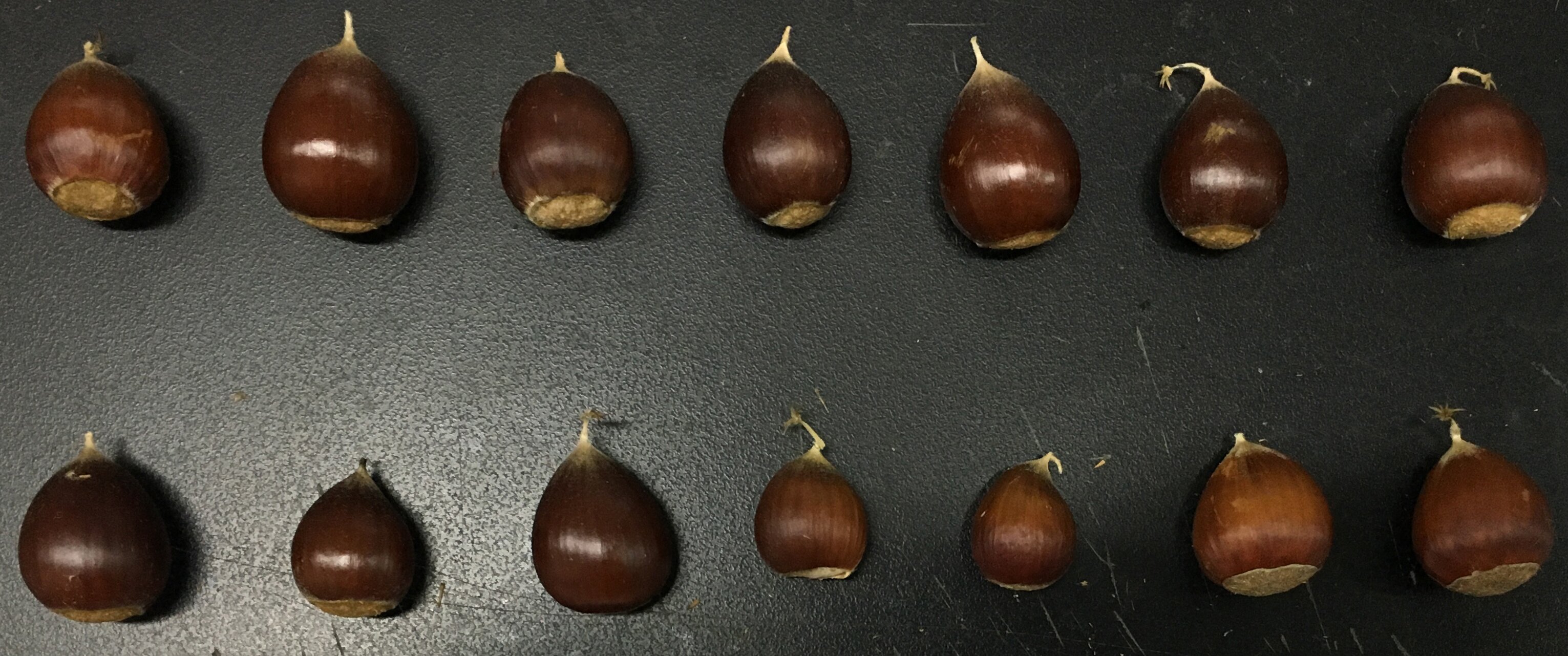

Supplement: Supplementary file 1 [file cimb-48-00173-s001.zip › File S12 Figure/File S10 Figure/Origin Figure/HTNC/HCTN-17.JPG]

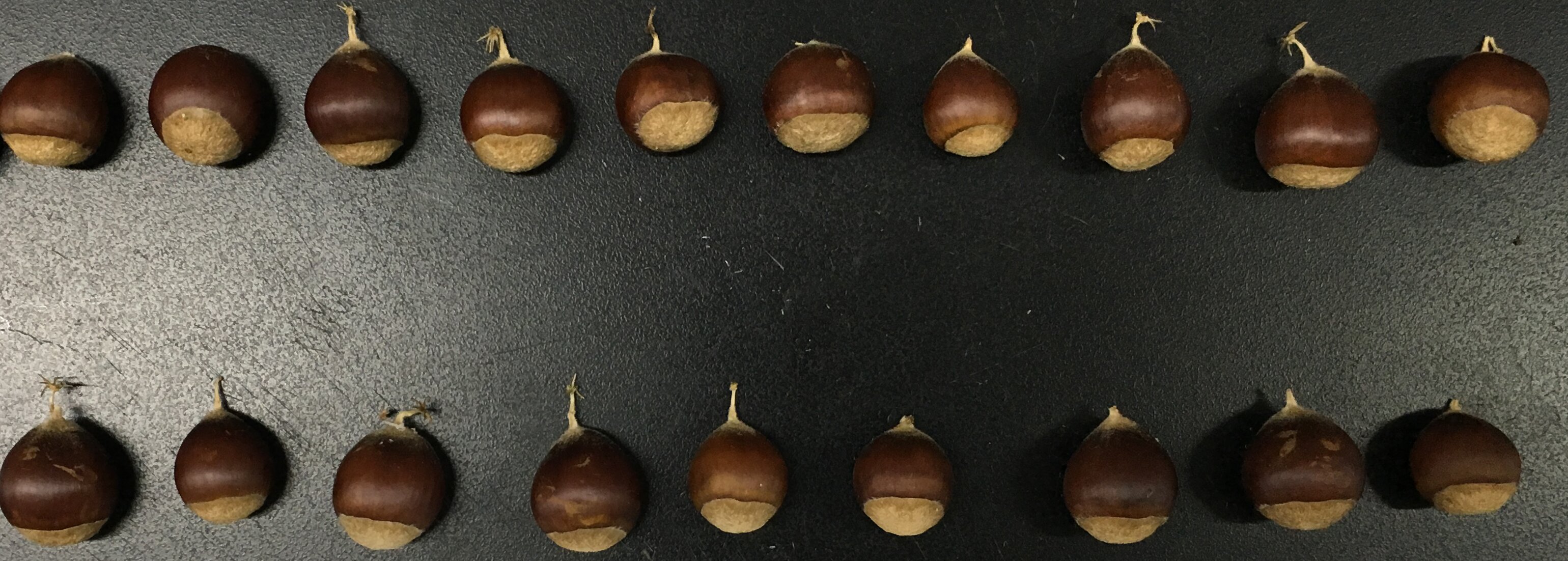

Supplement: Supplementary file 1 [file cimb-48-00173-s001.zip › File S12 Figure/File S10 Figure/Origin Figure/HTNC/HCTN-18.JPG]

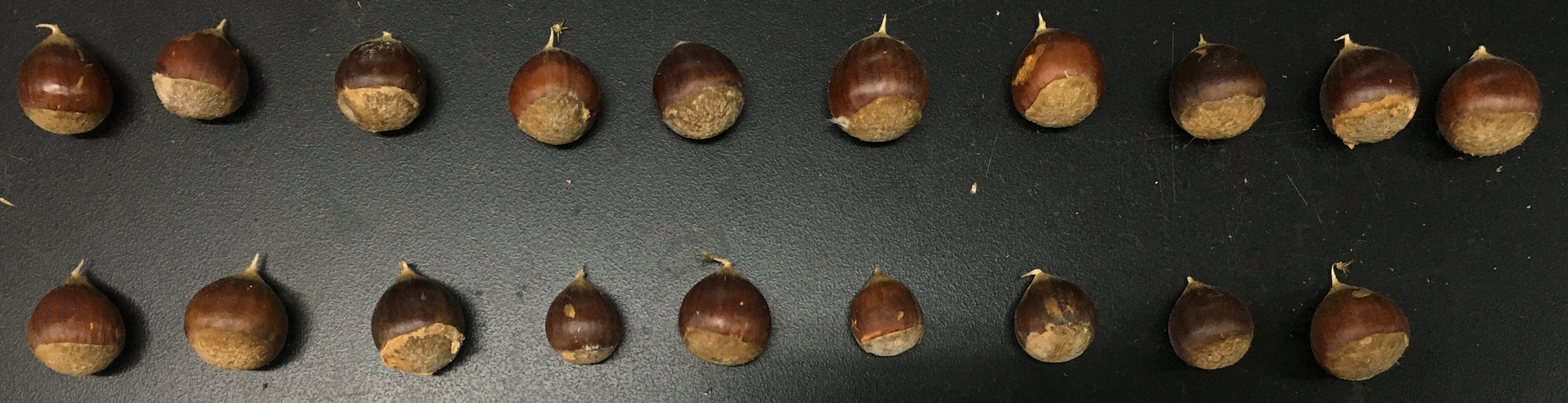

Supplement: Supplementary file 1 [file cimb-48-00173-s001.zip › File S12 Figure/File S10 Figure/Origin Figure/HTNC/HCTN-19.JPG]

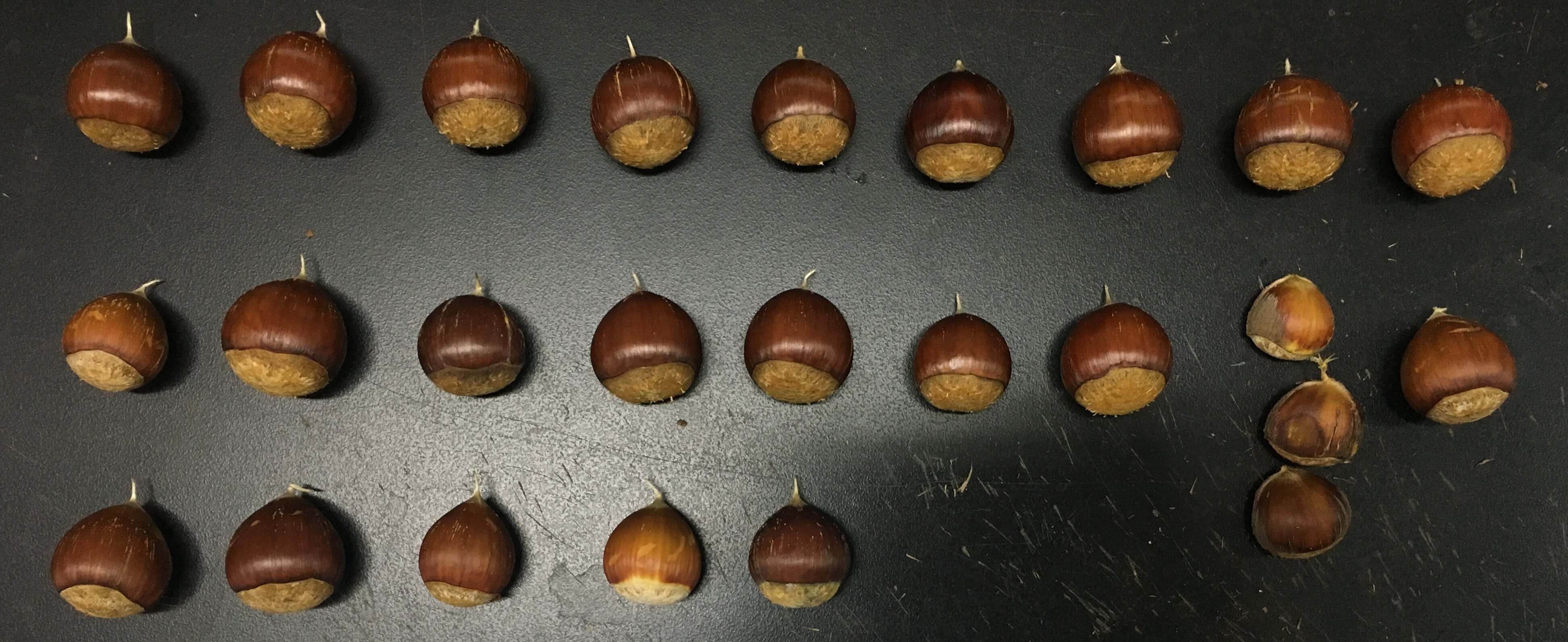

Supplement: Supplementary file 1 [file cimb-48-00173-s001.zip › File S12 Figure/File S10 Figure/Origin Figure/HTNC/HCTN-2.JPG]

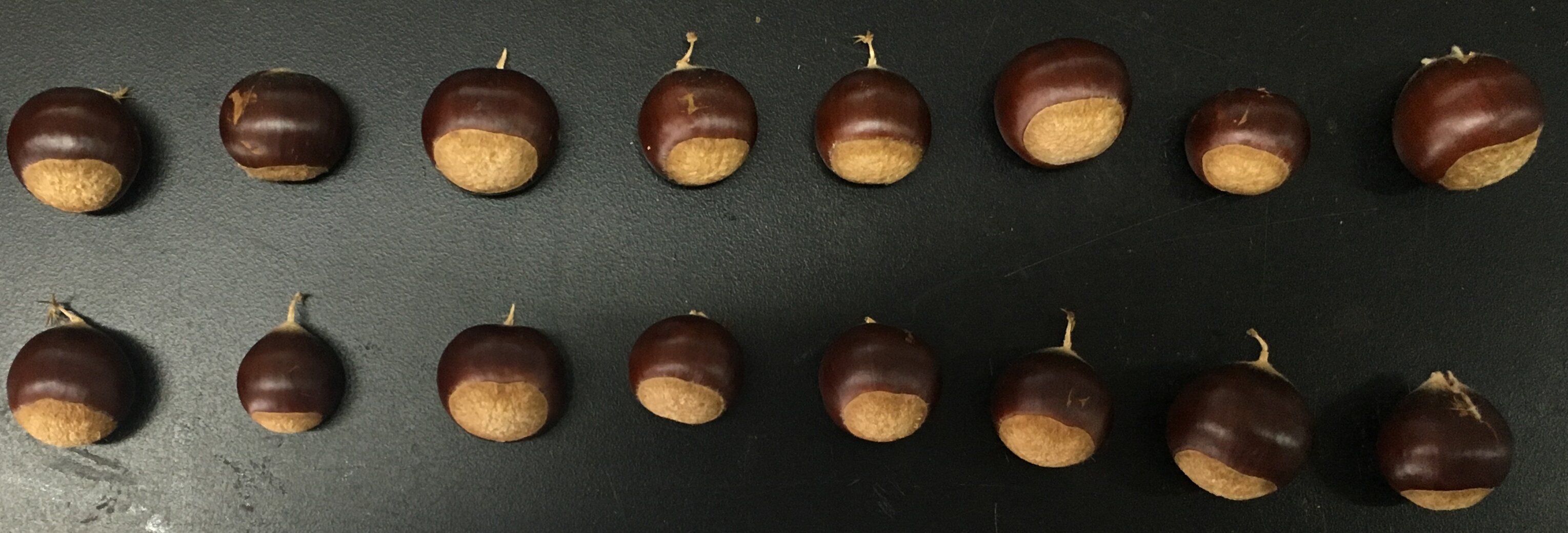

Supplement: Supplementary file 1 [file cimb-48-00173-s001.zip › File S12 Figure/File S10 Figure/Origin Figure/HTNC/HCTN-21.JPG]

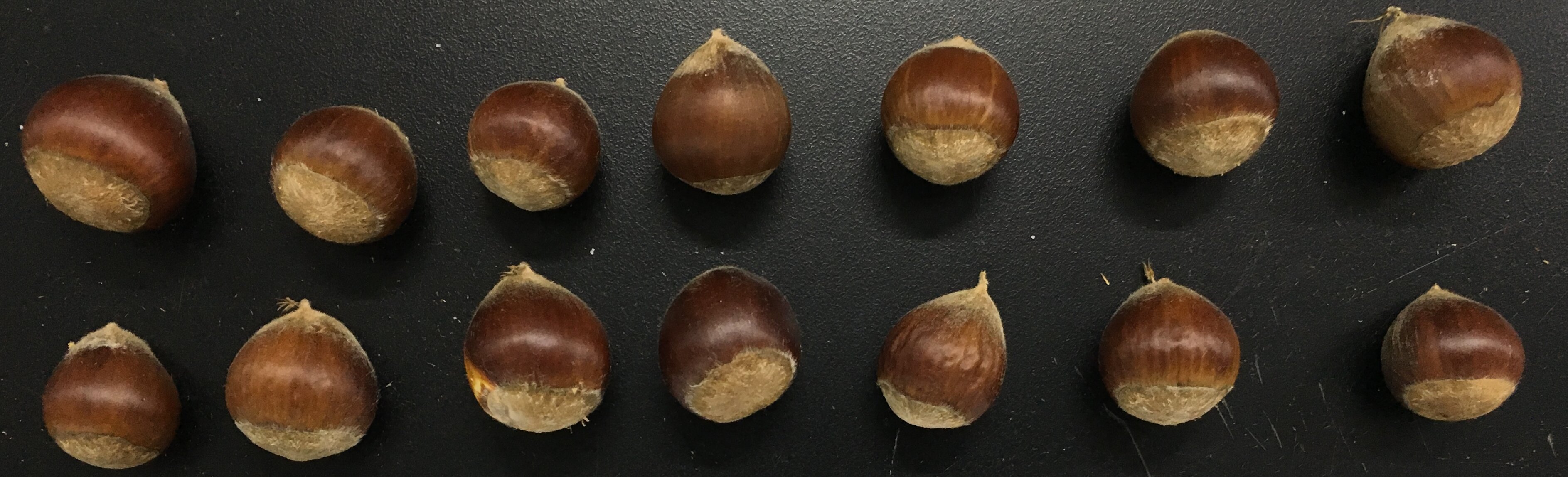

Supplement: Supplementary file 1 [file cimb-48-00173-s001.zip › File S12 Figure/File S10 Figure/Origin Figure/HTNC/HCTN-22.JPG]

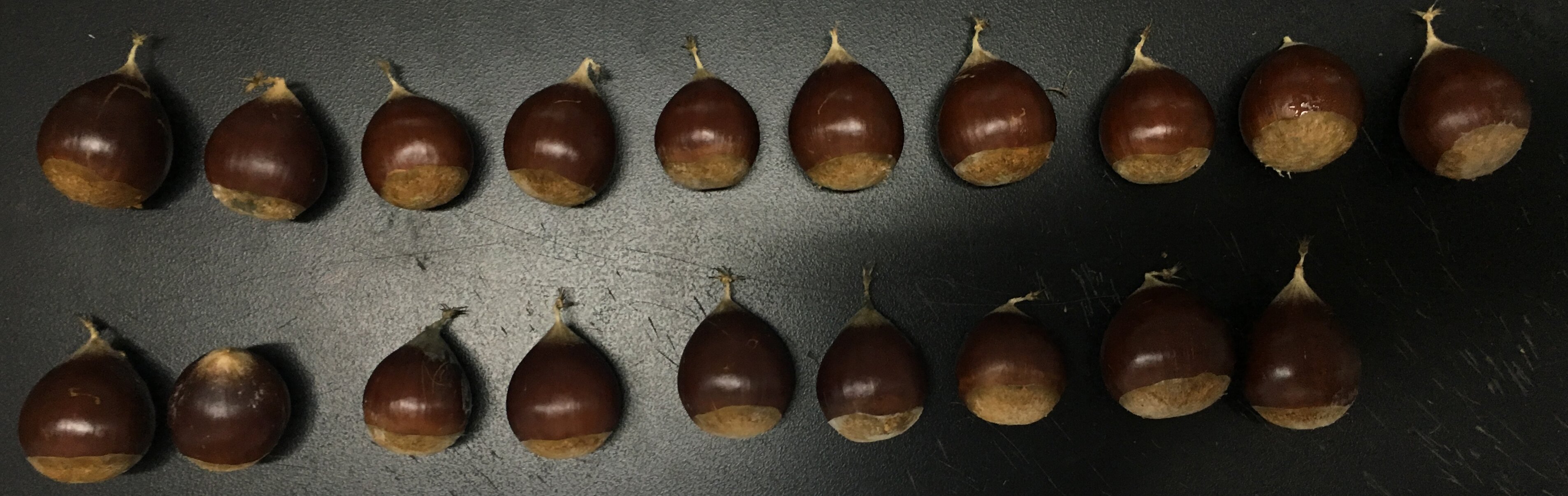

Supplement: Supplementary file 1 [file cimb-48-00173-s001.zip › File S12 Figure/File S10 Figure/Origin Figure/HTNC/HCTN-23.JPG]

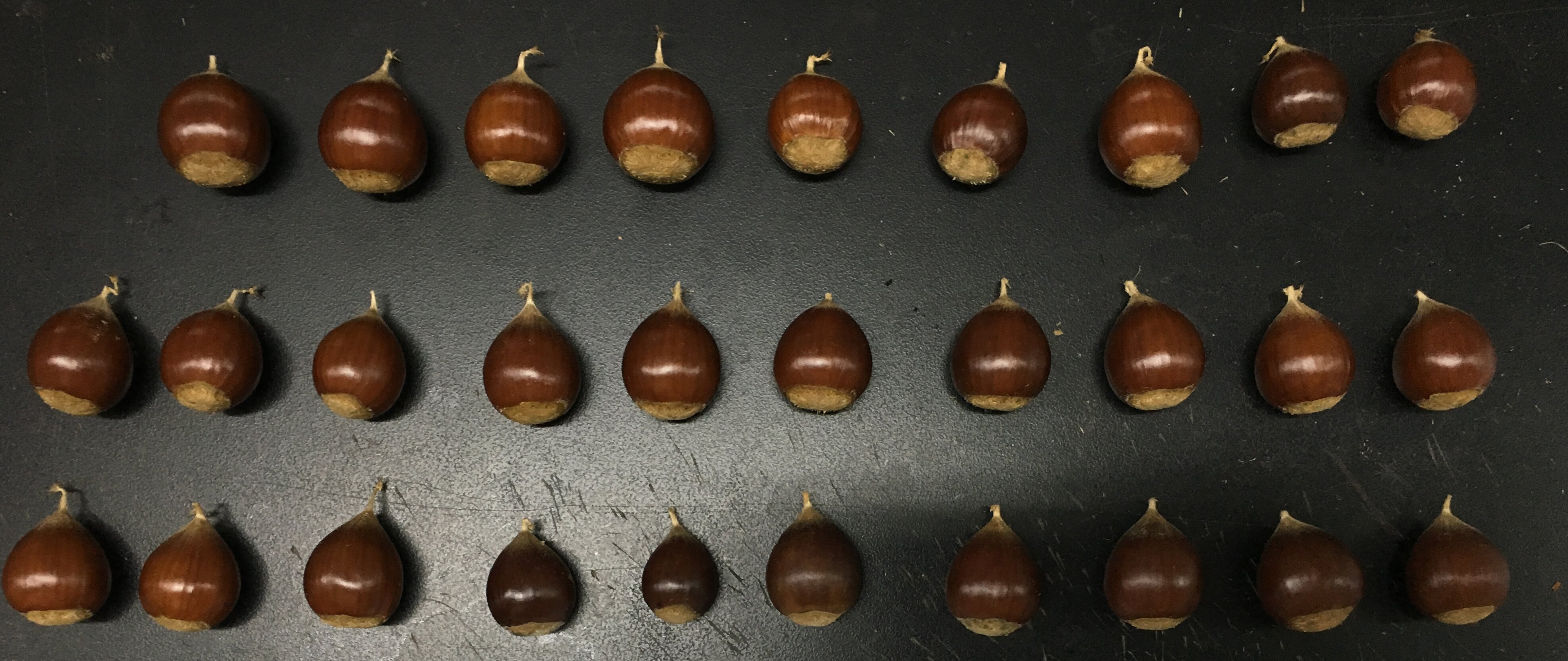

Supplement: Supplementary file 1 [file cimb-48-00173-s001.zip › File S12 Figure/File S10 Figure/Origin Figure/HTNC/HCTN-24.JPG]

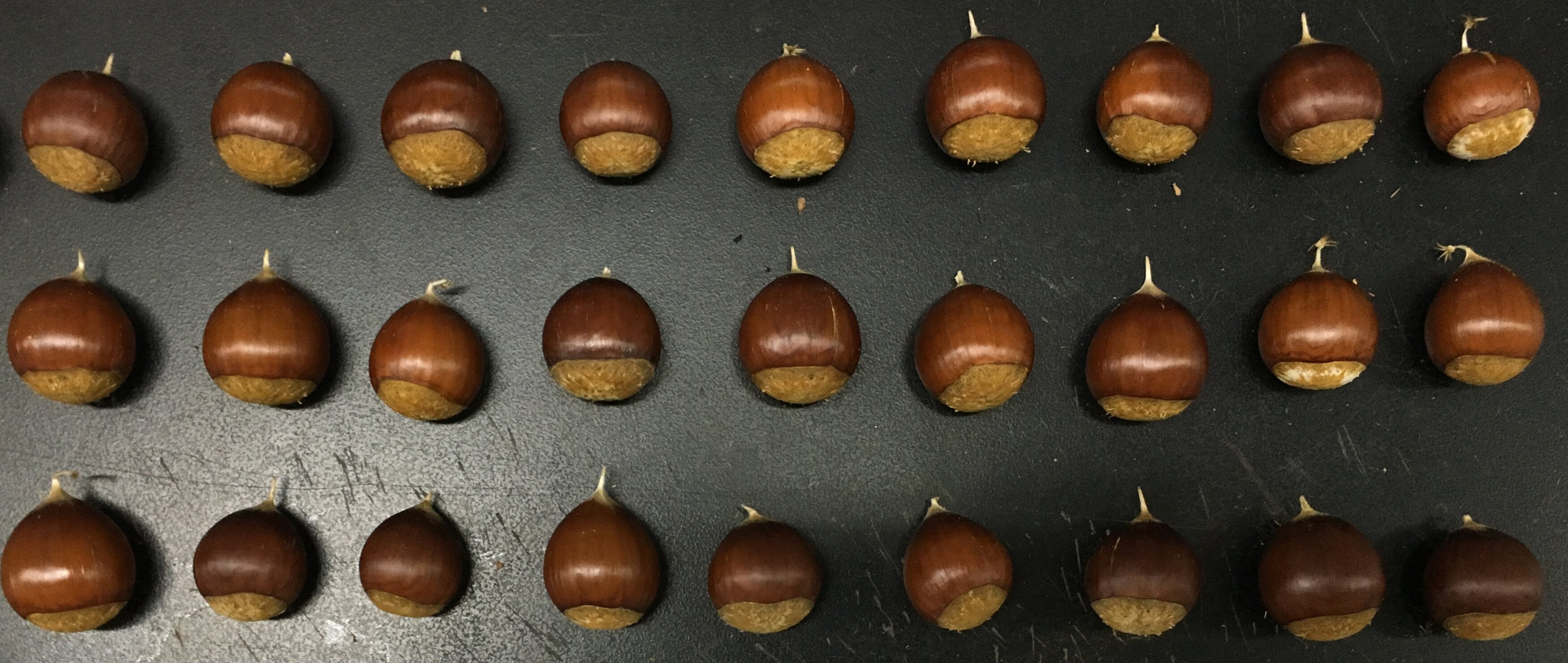

Supplement: Supplementary file 1 [file cimb-48-00173-s001.zip › File S12 Figure/File S10 Figure/Origin Figure/HTNC/HCTN-25.JPG]

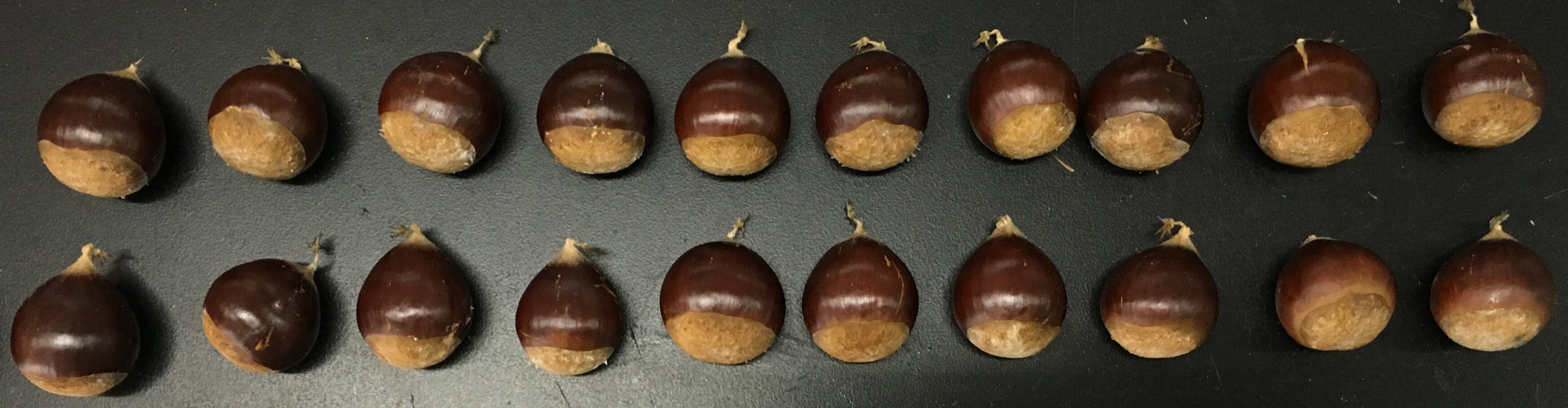

Supplement: Supplementary file 1 [file cimb-48-00173-s001.zip › File S12 Figure/File S10 Figure/Origin Figure/HTNC/HCTN-26.JPG]

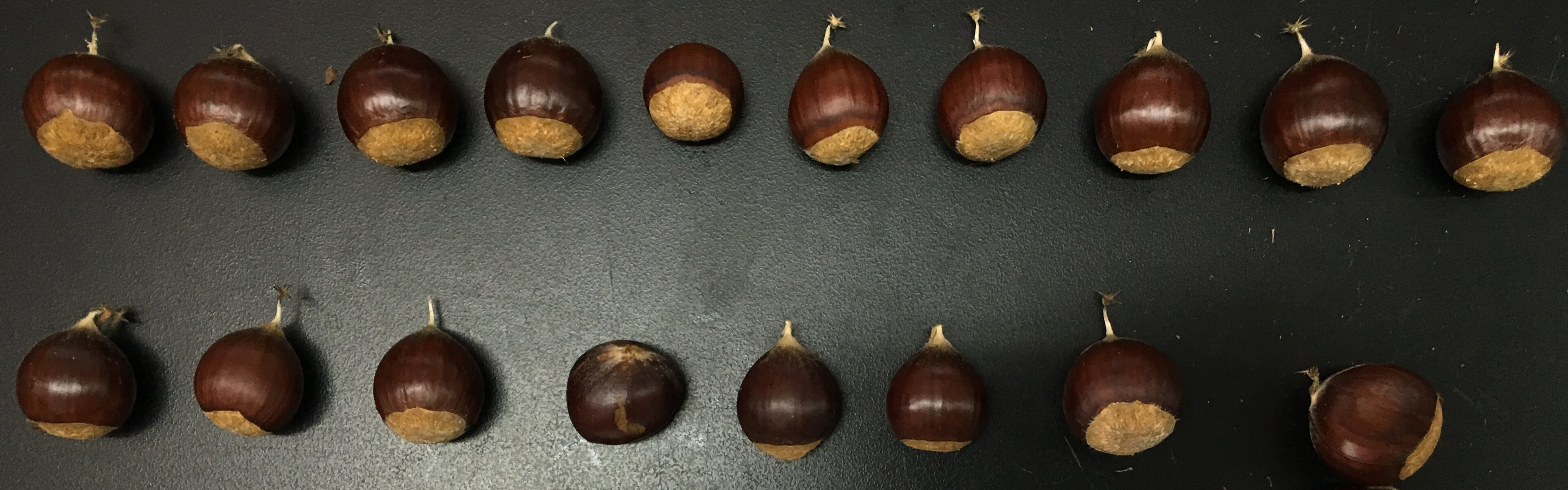

Supplement: Supplementary file 1 [file cimb-48-00173-s001.zip › File S12 Figure/File S10 Figure/Origin Figure/HTNC/HCTN-3.JPG]

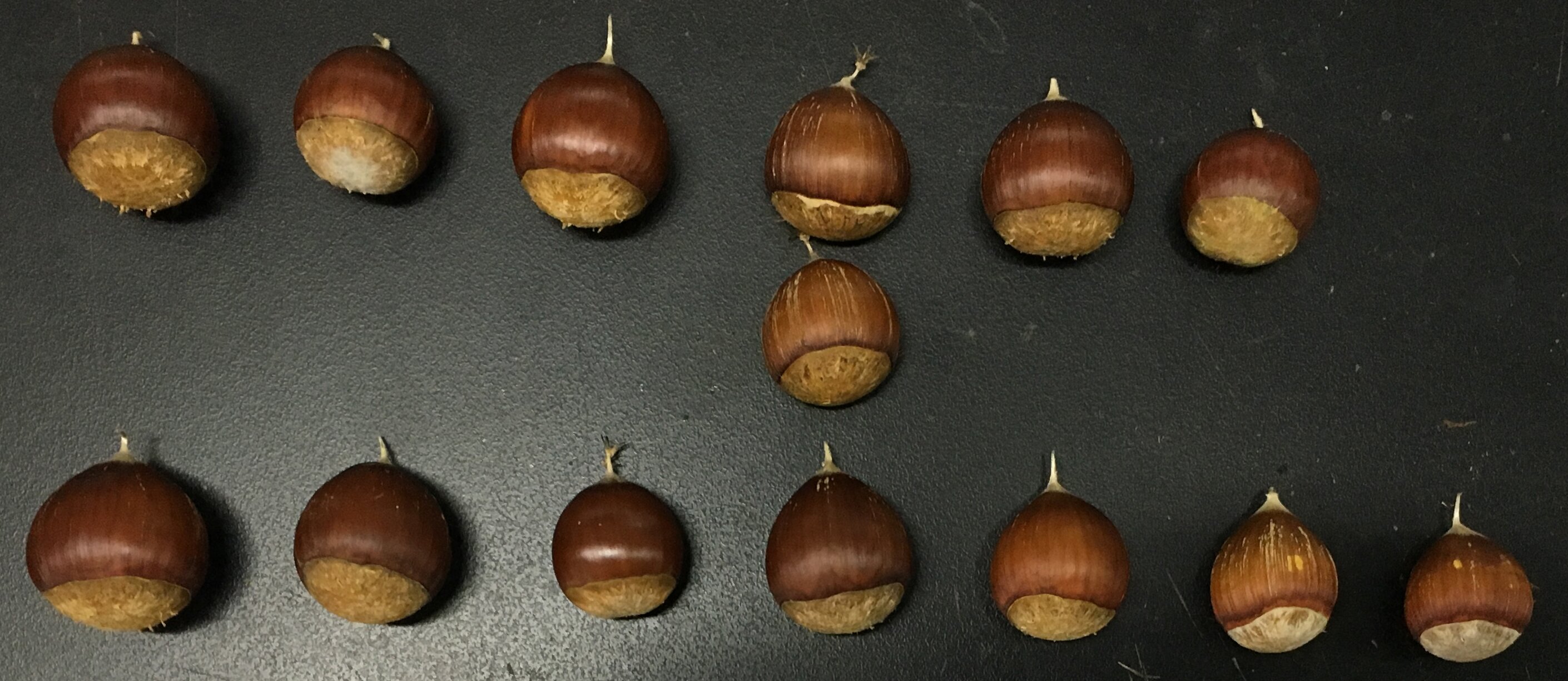

Supplement: Supplementary file 1 [file cimb-48-00173-s001.zip › File S12 Figure/File S10 Figure/Origin Figure/HTNC/HCTN-4.JPG]

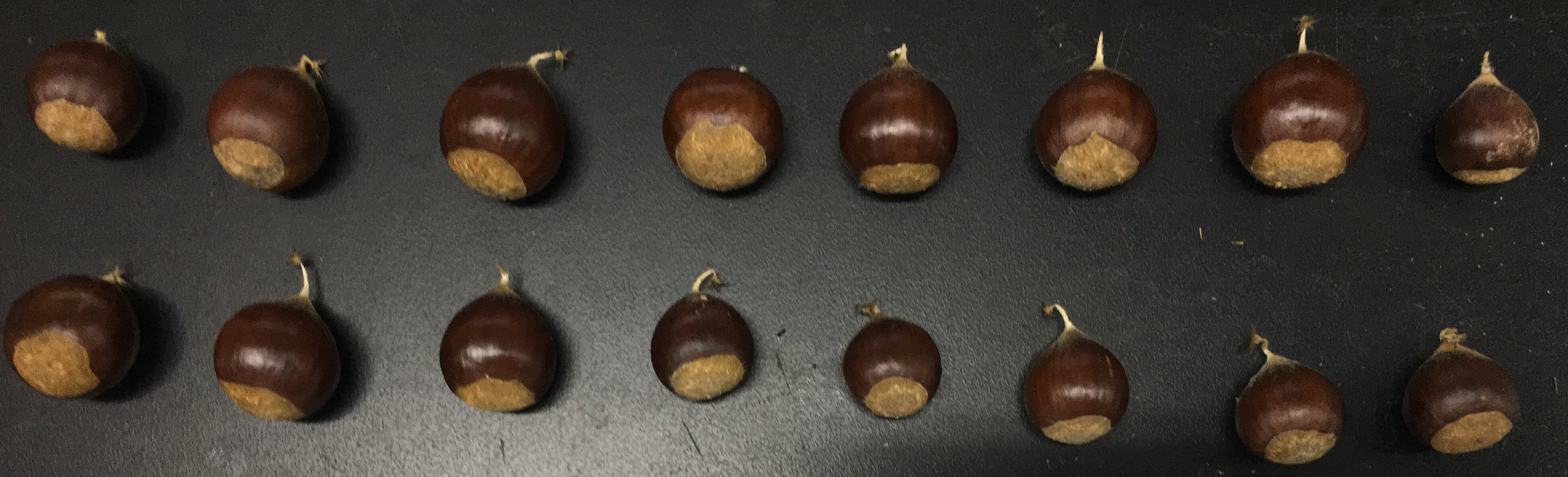

Supplement: Supplementary file 1 [file cimb-48-00173-s001.zip › File S12 Figure/File S10 Figure/Origin Figure/HTNC/HCTN-5.JPG]

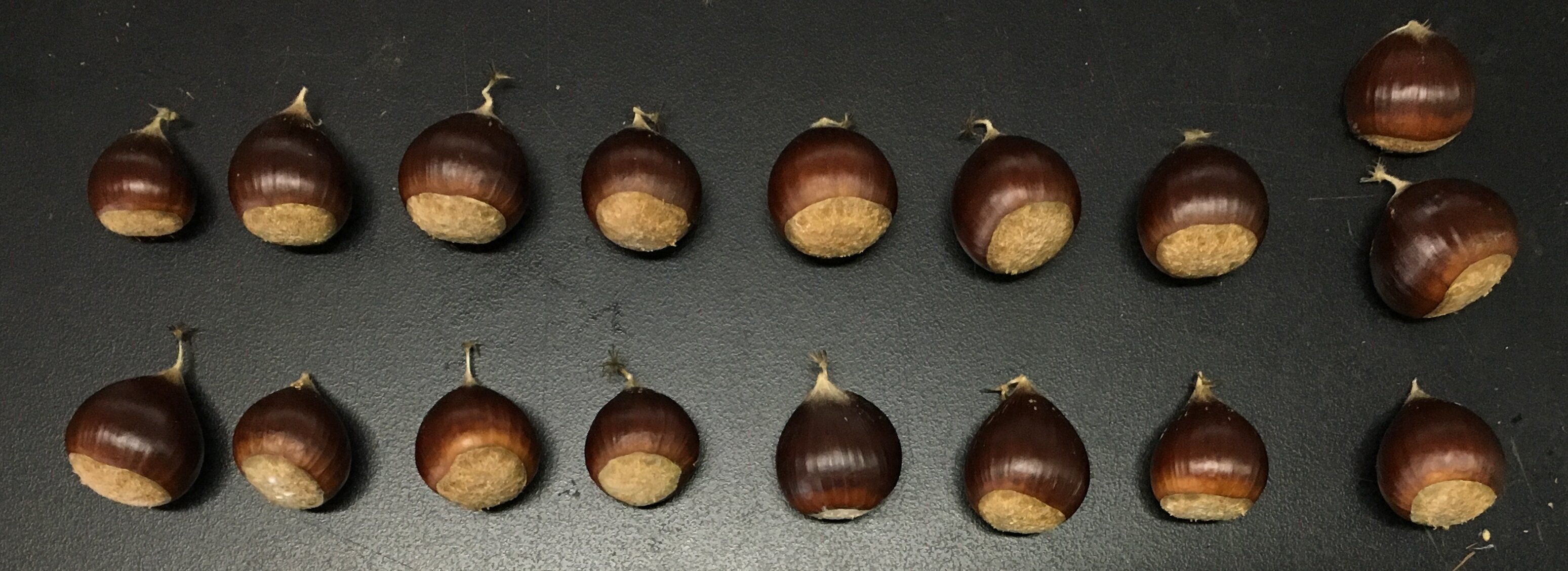

Supplement: Supplementary file 1 [file cimb-48-00173-s001.zip › File S12 Figure/File S10 Figure/Origin Figure/HTNC/HCTN-6.JPG]

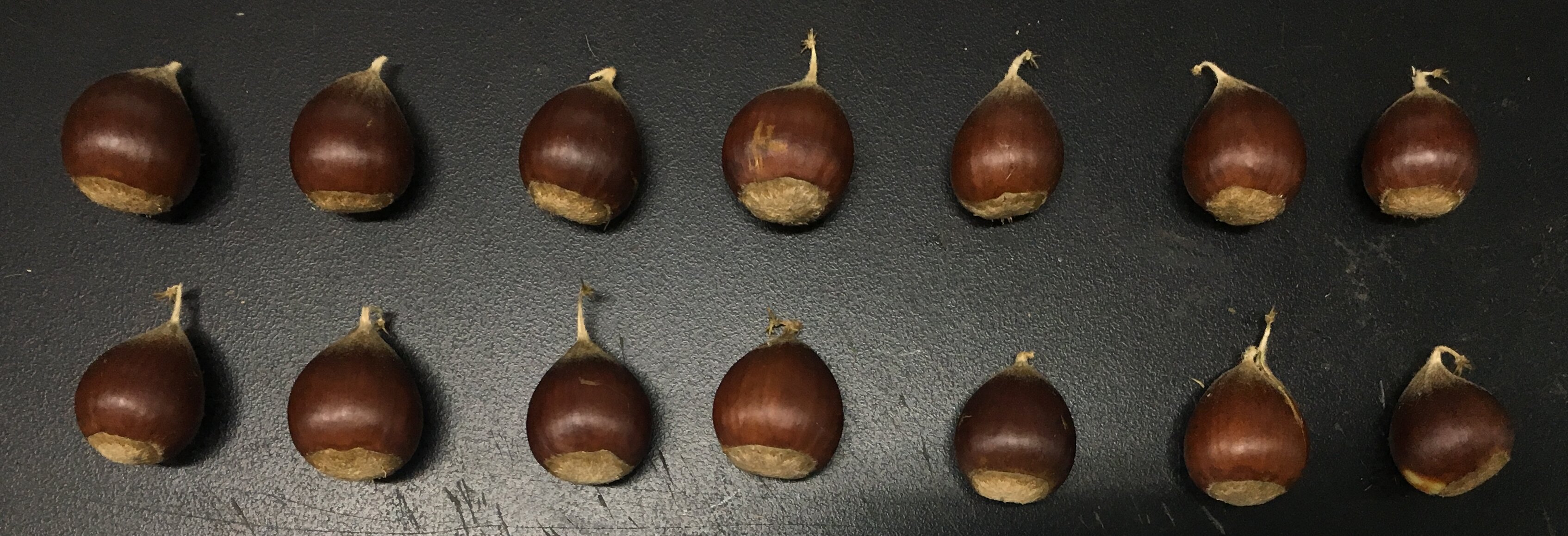

Supplement: Supplementary file 1 [file cimb-48-00173-s001.zip › File S12 Figure/File S10 Figure/Origin Figure/HTNC/HCTN-7.JPG]

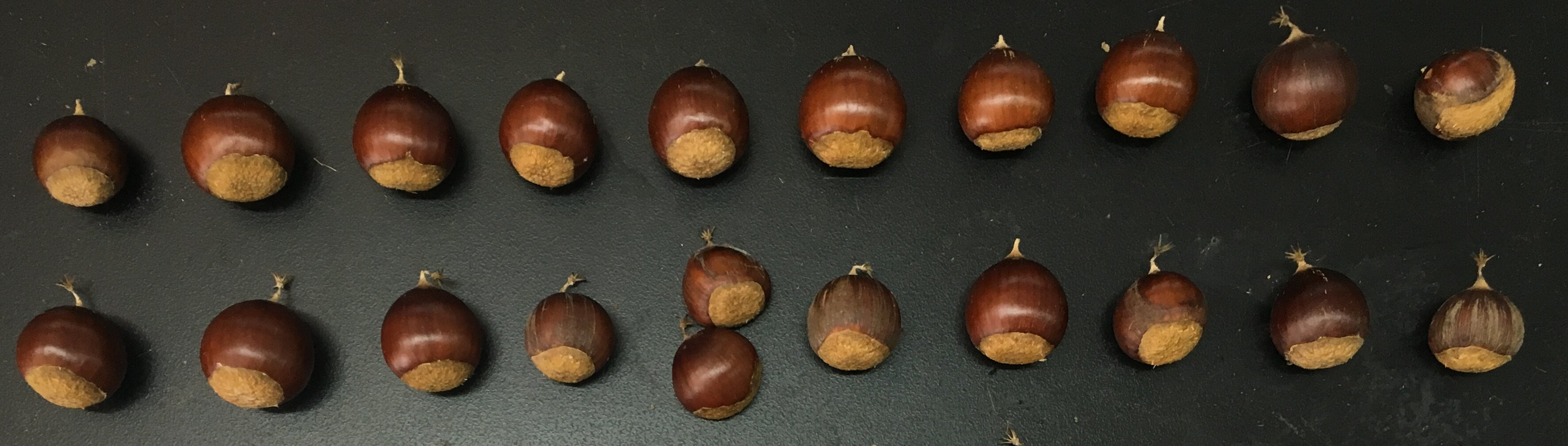

Supplement: Supplementary file 1 [file cimb-48-00173-s001.zip › File S12 Figure/File S10 Figure/Origin Figure/HTNC/HCTN-8.JPG]

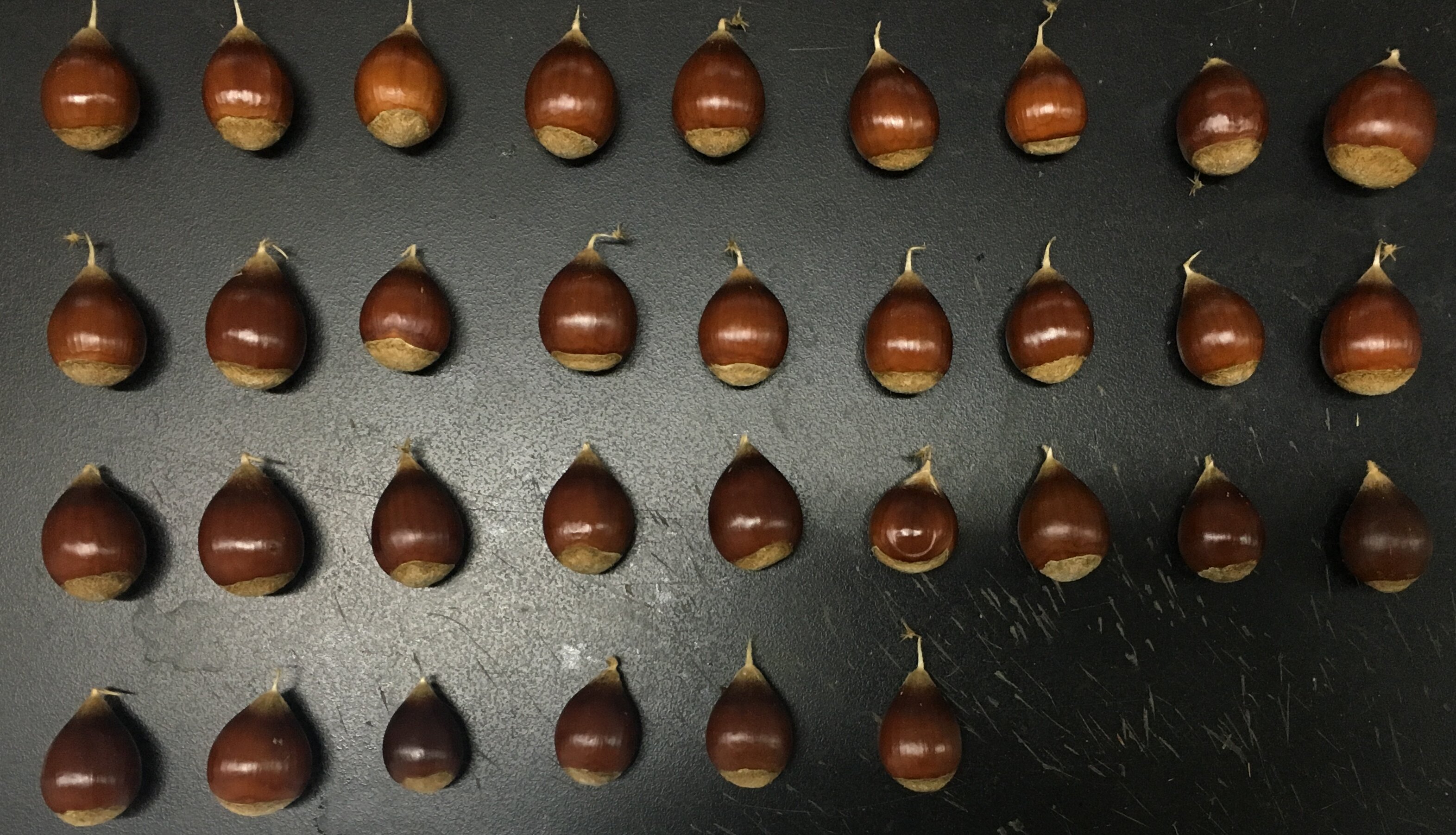

Supplement: Supplementary file 1 [file cimb-48-00173-s001.zip › File S12 Figure/File S10 Figure/Origin Figure/HTNC/HCTN-9.JPG]

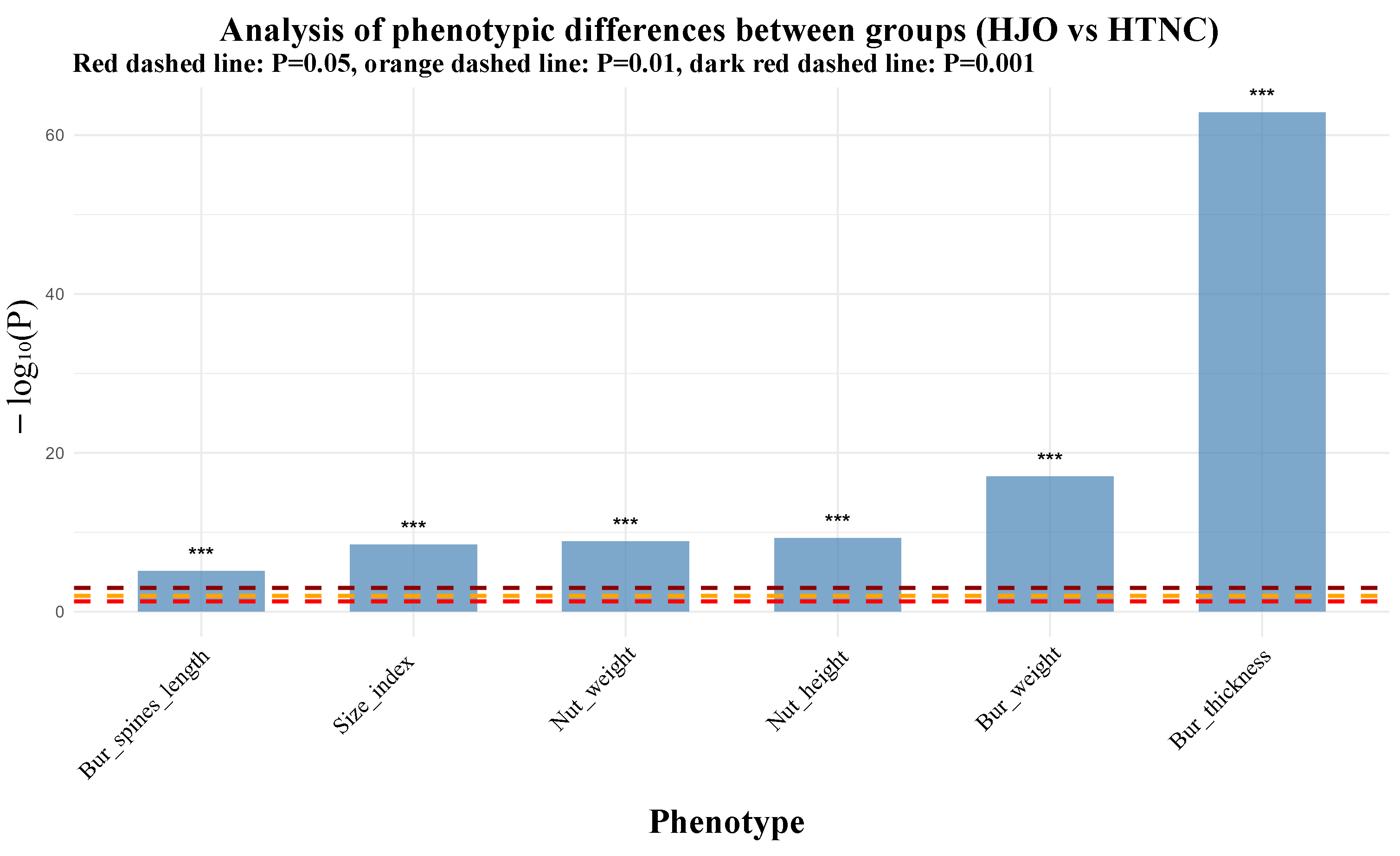

Supplement: Supplementary file 1 [file cimb-48-00173-s001.zip › File S4 Permanova_results/File S3 Permanova_results/Statistical results of PERMANOVA analysis for individual traits/cimb-4087289-supplementary-File S4 Analysis of phenotypic differences.jpg]
